# Supplementary material for: Heterochromatin epimutations impose mitochondrial dysfunction to confer antifungal resistance
Source: EMBO J. 2025 Dec 1;45(2):417–48. doi: 10.1038/s44318-025-00649-0 (PMC12811382; doi:10.1038/s44318-025-00649-0)
Supplement: Supplementary file 5 — Source data Fig. 1 [file 44318_2025_649_MOESM5_ESM.zip › 121174_Source_Data_Fig_1/Fig_1A/README_Fig1A.docx]

Fig. 1A: H3K9me2 ChIP-Seq data plotted from GEO: GSE138436; GSM4107922 (Torres-Garcia et al., 2020b).
