# Supplementary figures and images for: Heterochromatin epimutations impose mitochondrial dysfunction to confer antifungal resistance (part 1 of 2)
Source: EMBO J. 2025 Dec 1;45(2):417–48. doi: 10.1038/s44318-025-00649-0 (PMC12811382; doi:10.1038/s44318-025-00649-0)

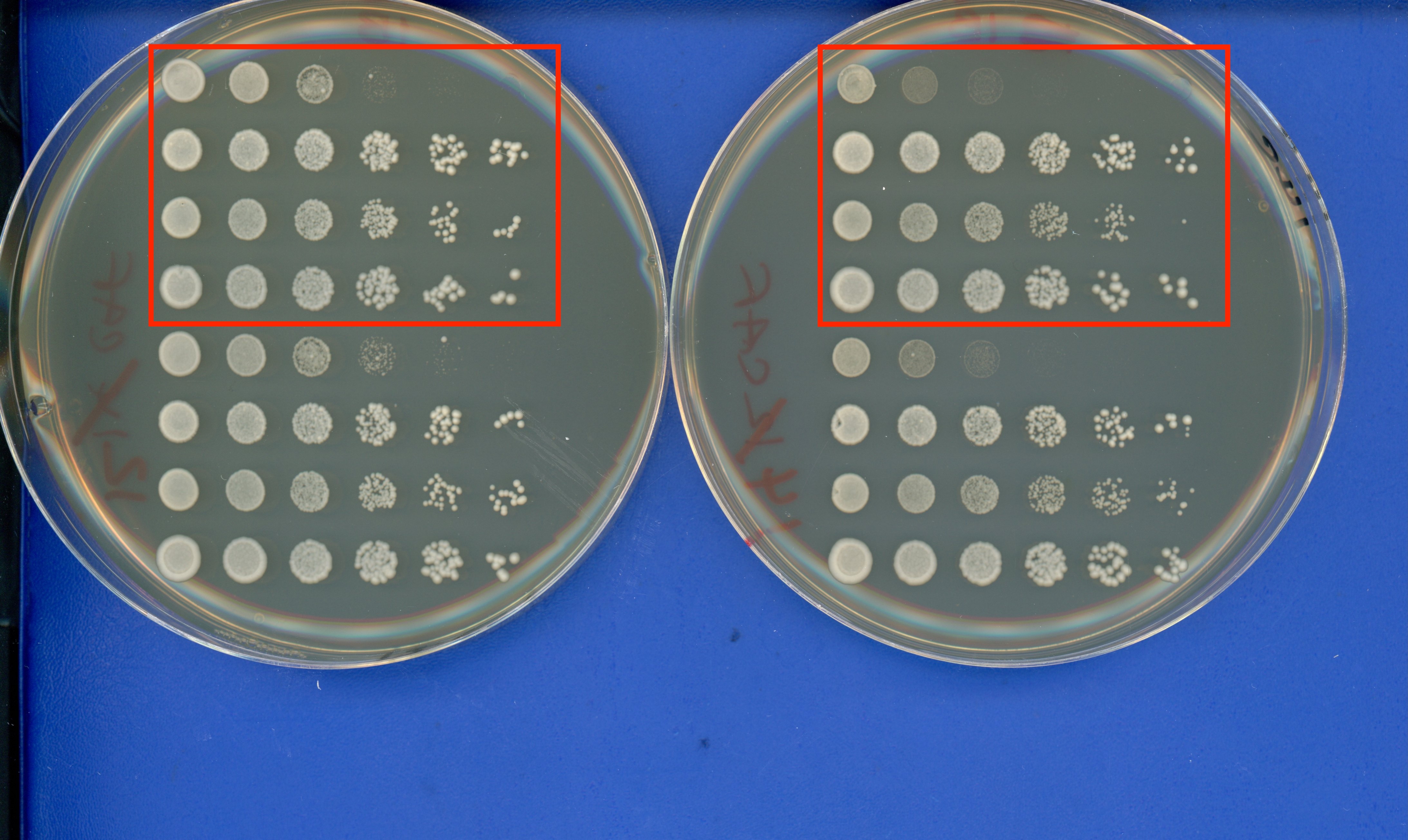

Supplement: Supplementary file 5 — Source data Fig. 1 [file 44318_2025_649_MOESM5_ESM.zip › 121174_Source_Data_Fig_1/Fig_1B/Fig1B_12_14CAF_annotated.jpg]

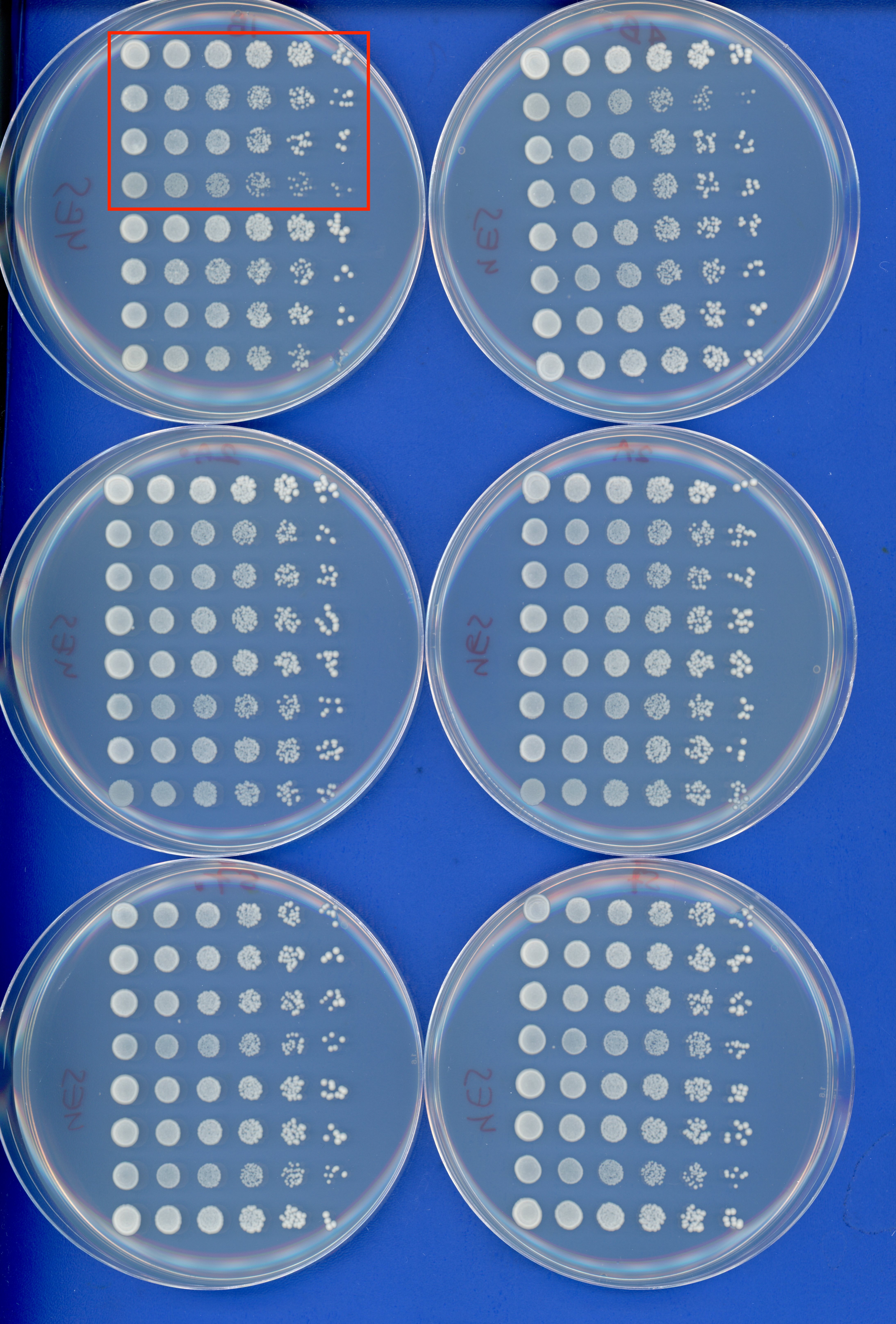

Supplement: Supplementary file 5 — Source data Fig. 1 [file 44318_2025_649_MOESM5_ESM.zip › 121174_Source_Data_Fig_1/Fig_1B/Fig1B_YES_annotated.jpg]

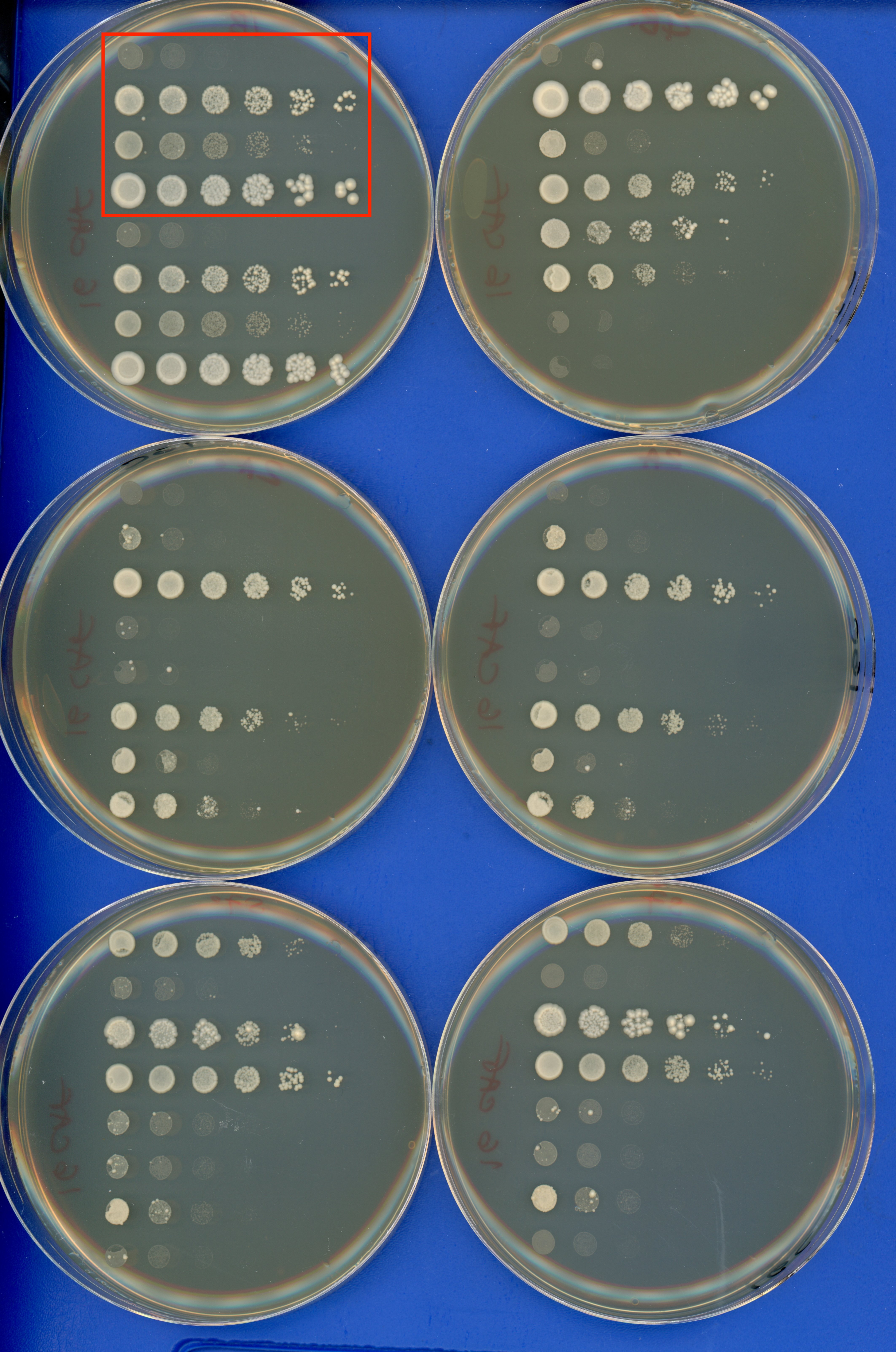

Supplement: Supplementary file 5 — Source data Fig. 1 [file 44318_2025_649_MOESM5_ESM.zip › 121174_Source_Data_Fig_1/Fig_1B/Fig1B_16CAF_annotated.jpg]

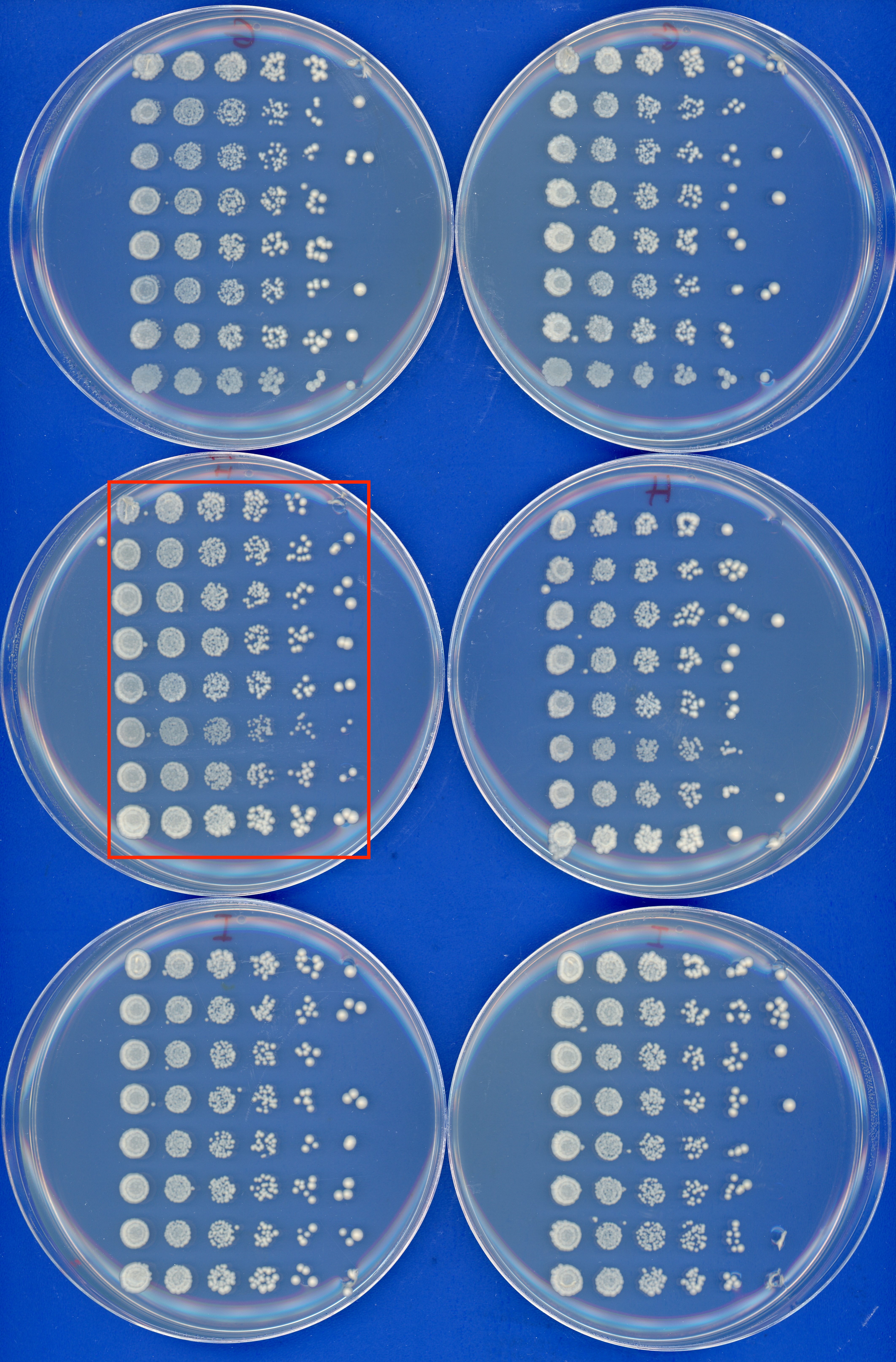

Supplement: Supplementary file 5 — Source data Fig. 1 [file 44318_2025_649_MOESM5_ESM.zip › 121174_Source_Data_Fig_1/Fig_1A/Fig1A_YES_annotated.jpg]

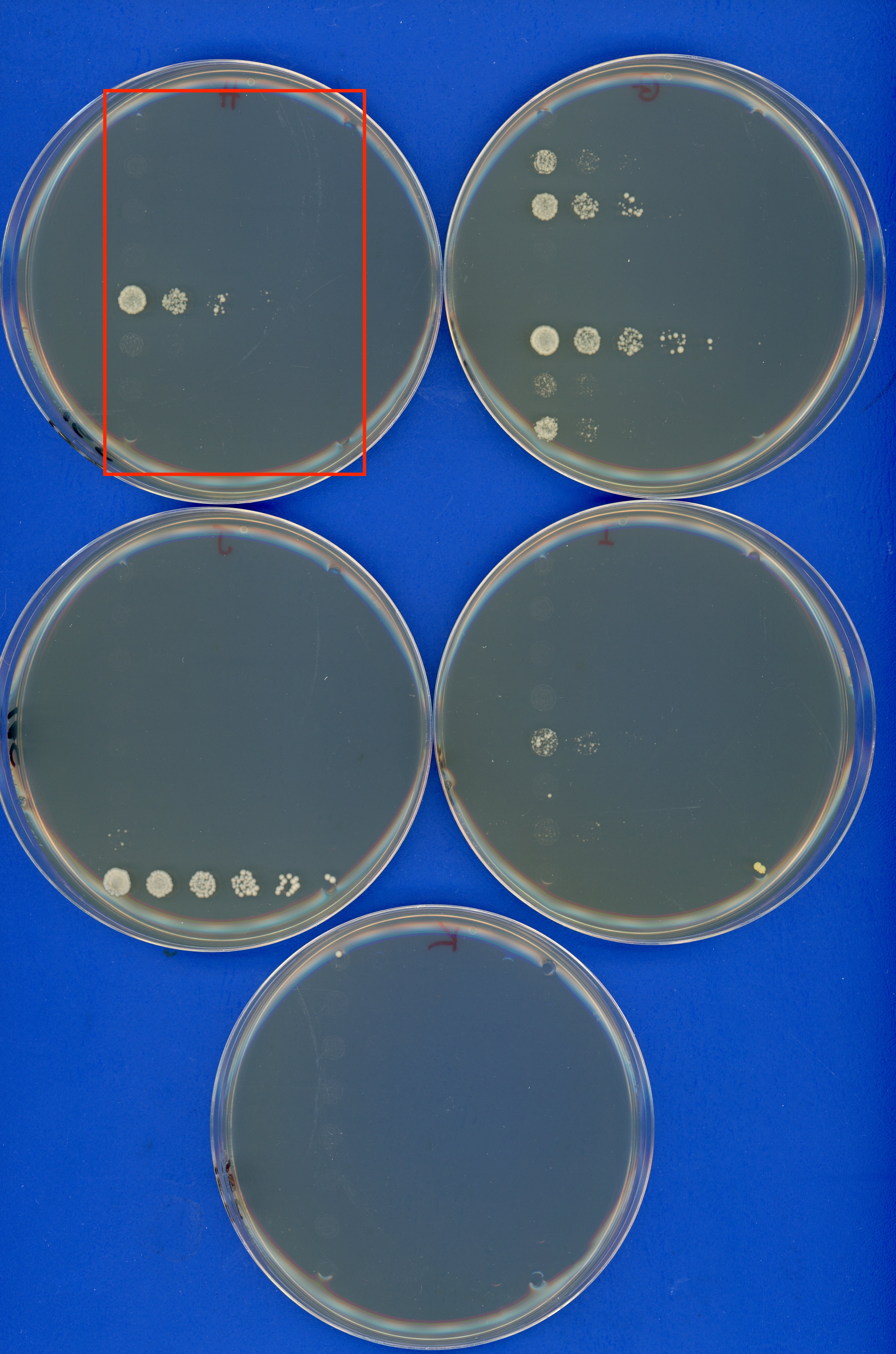

Supplement: Supplementary file 5 — Source data Fig. 1 [file 44318_2025_649_MOESM5_ESM.zip › 121174_Source_Data_Fig_1/Fig_1A/Fig1A_16CAF_annotated.jpg]

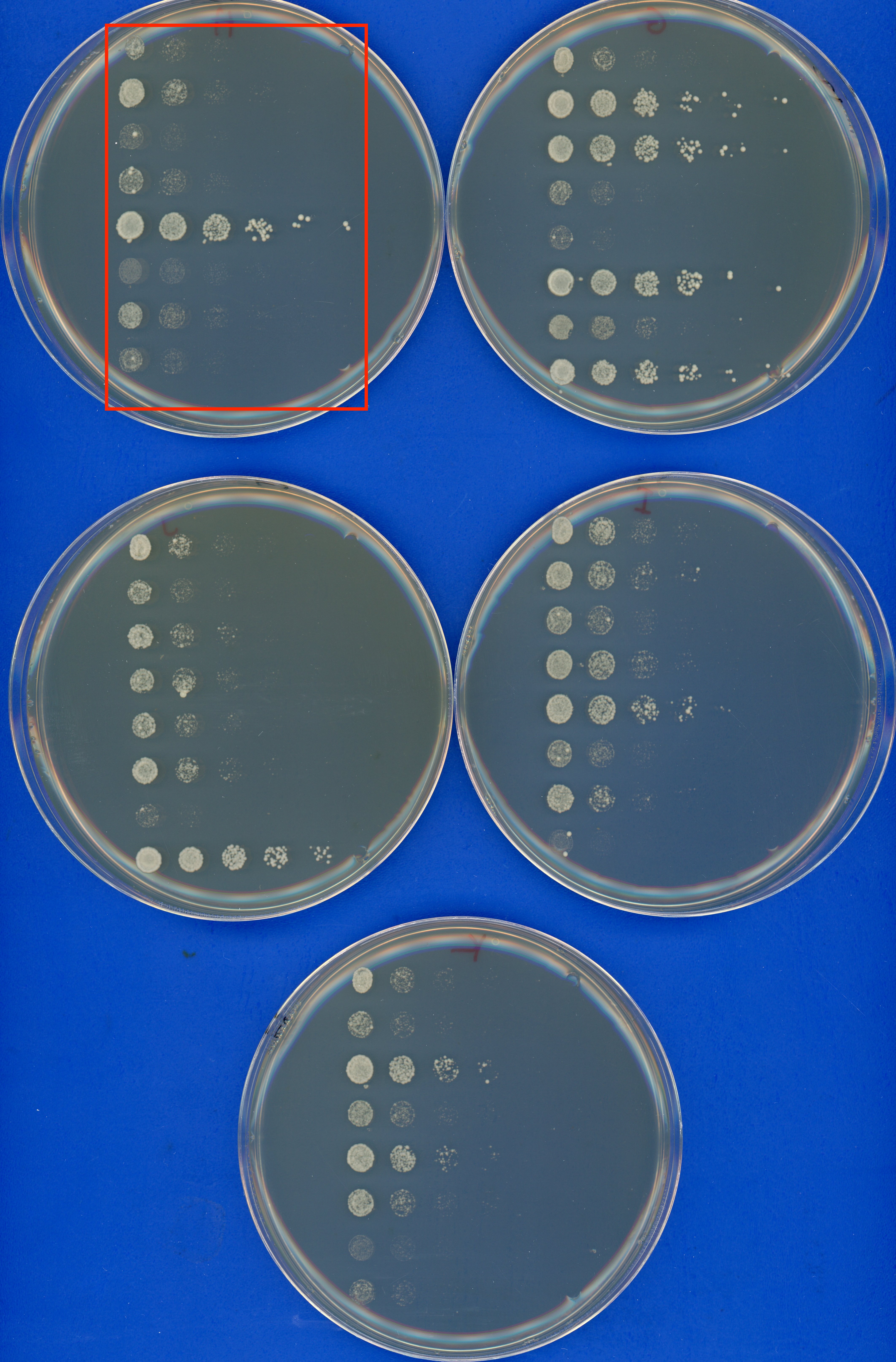

Supplement: Supplementary file 5 — Source data Fig. 1 [file 44318_2025_649_MOESM5_ESM.zip › 121174_Source_Data_Fig_1/Fig_1A/Fig1A_12CAF_annotated.jpg]

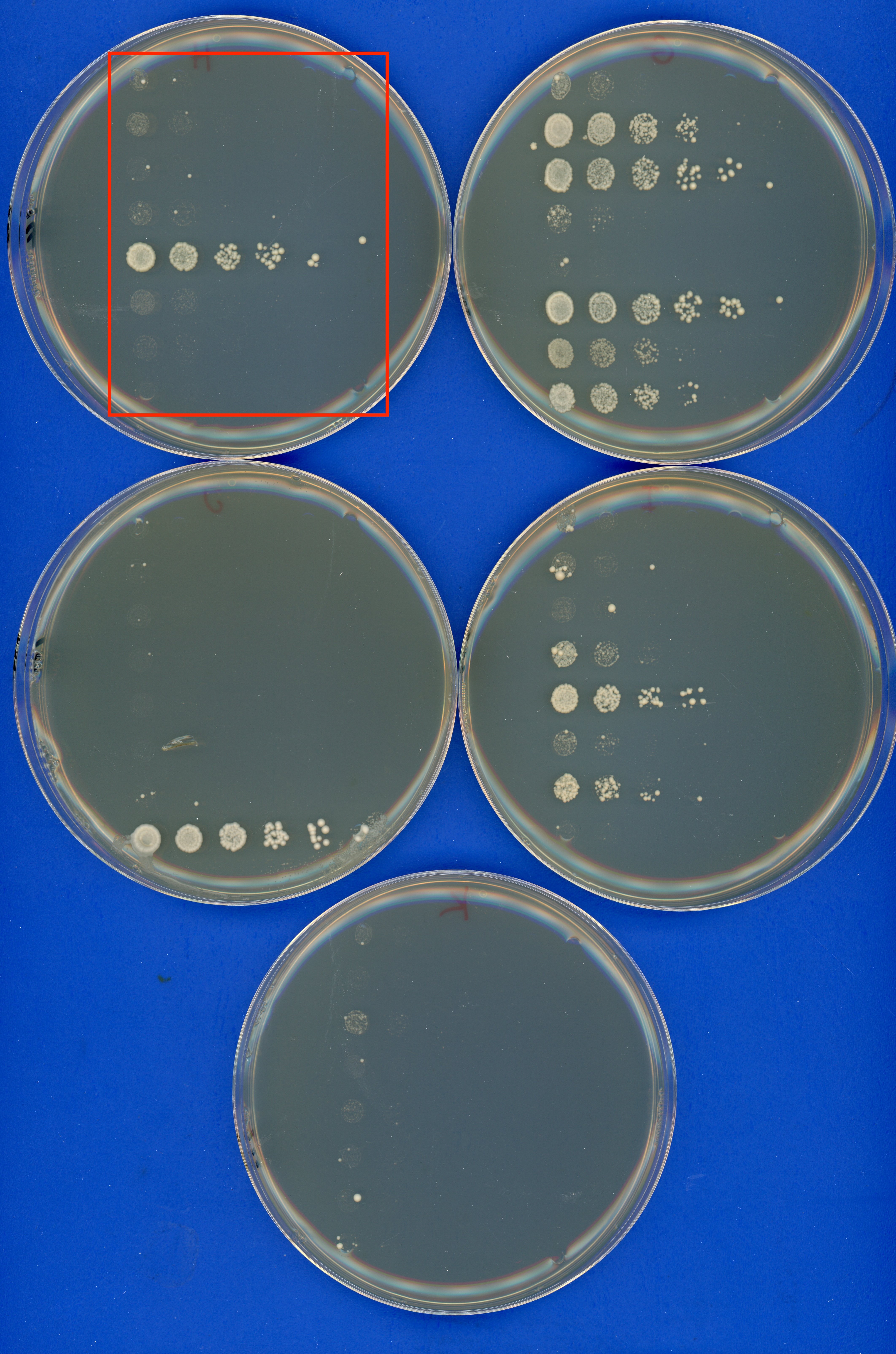

Supplement: Supplementary file 5 — Source data Fig. 1 [file 44318_2025_649_MOESM5_ESM.zip › 121174_Source_Data_Fig_1/Fig_1A/Fig1A_14CAF_annotated.jpg]

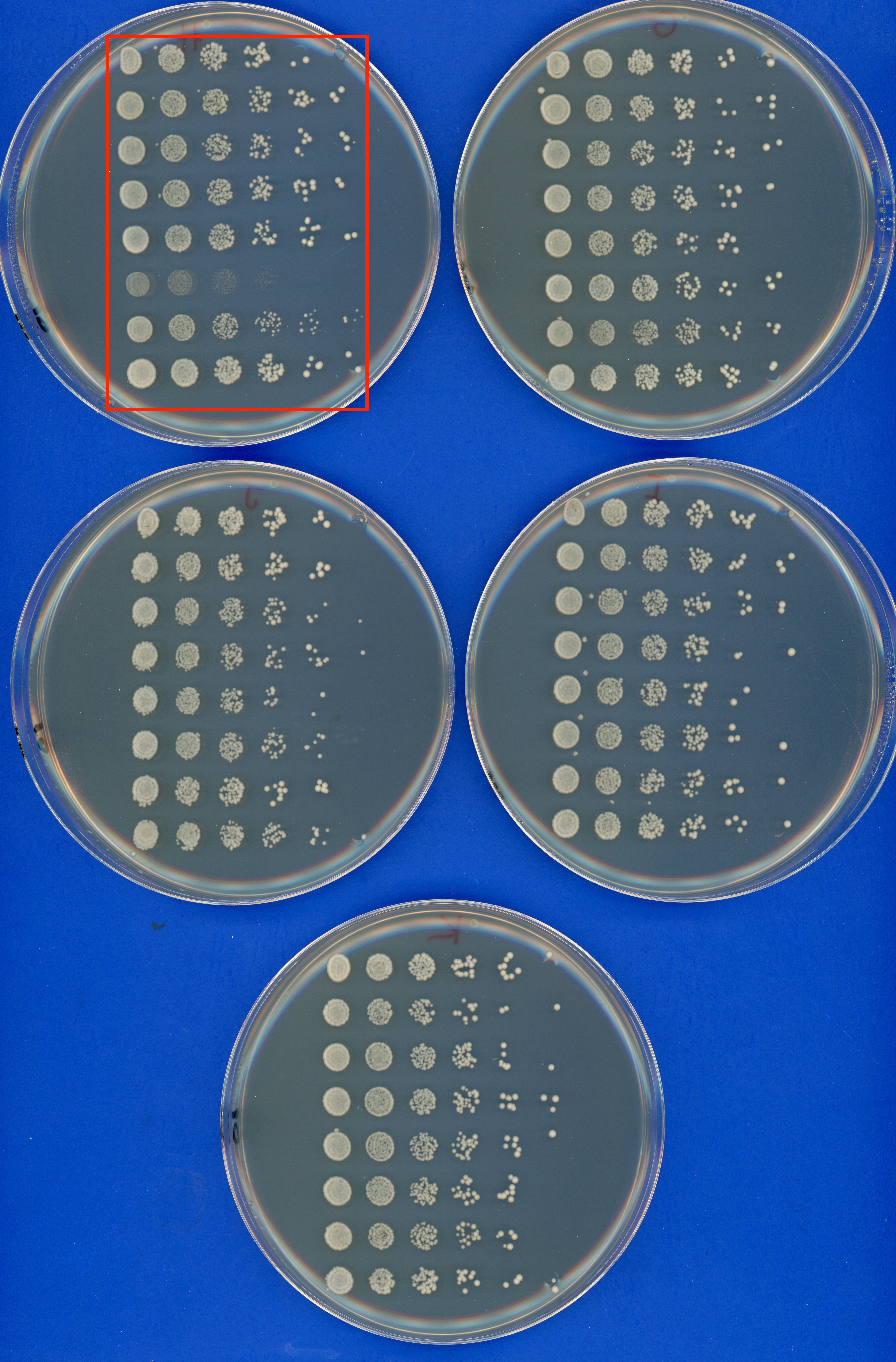

Supplement: Supplementary file 5 — Source data Fig. 1 [file 44318_2025_649_MOESM5_ESM.zip › 121174_Source_Data_Fig_1/Fig_1A/Fig1A_10CAF_annotated.jpg]

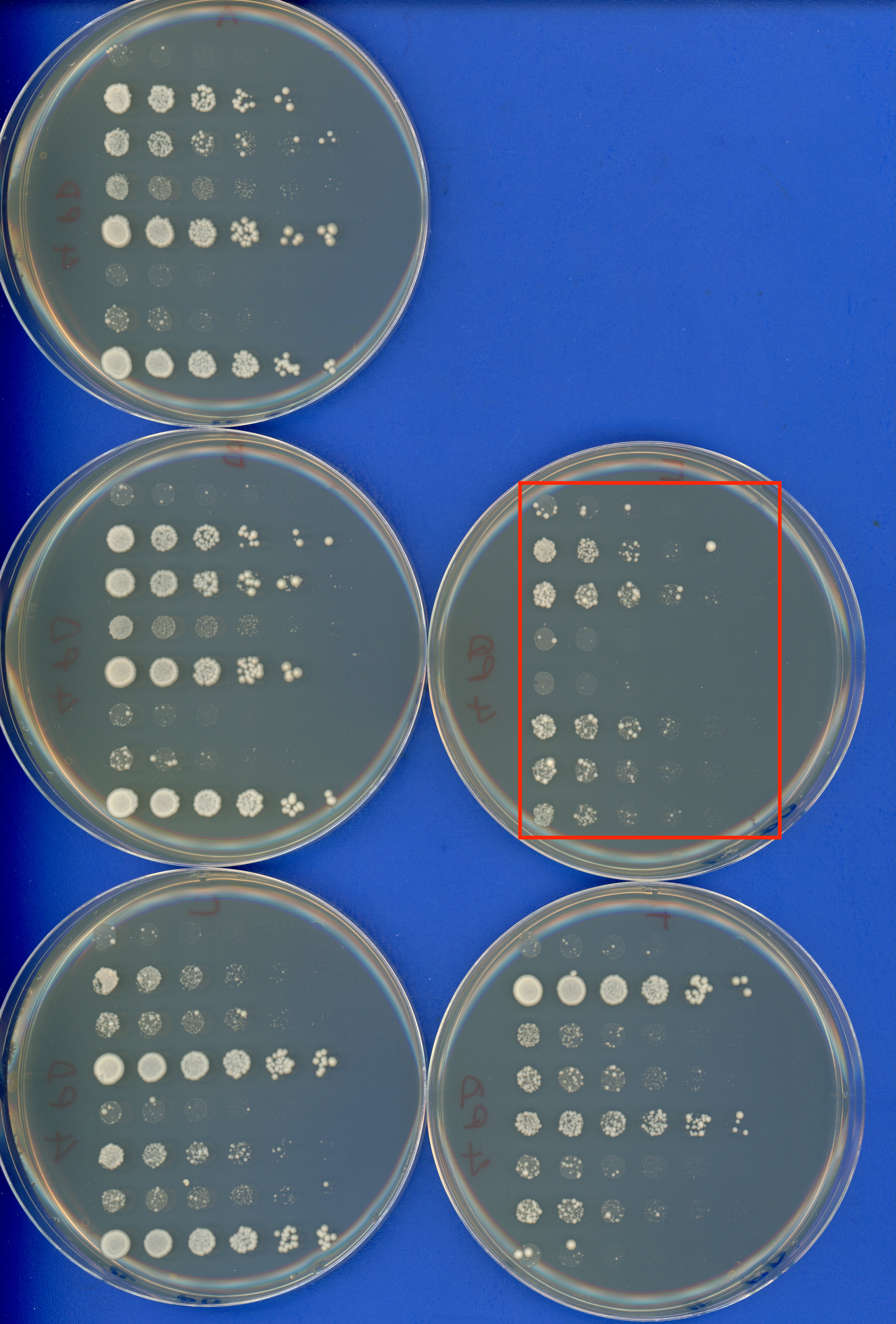

Supplement: Supplementary file 6 — Source data Fig. 2 [file 44318_2025_649_MOESM6_ESM.zip › 121174_Source_Data_Fig_2/Fig_2B/Fig2B_PRD_annotated.jpg]

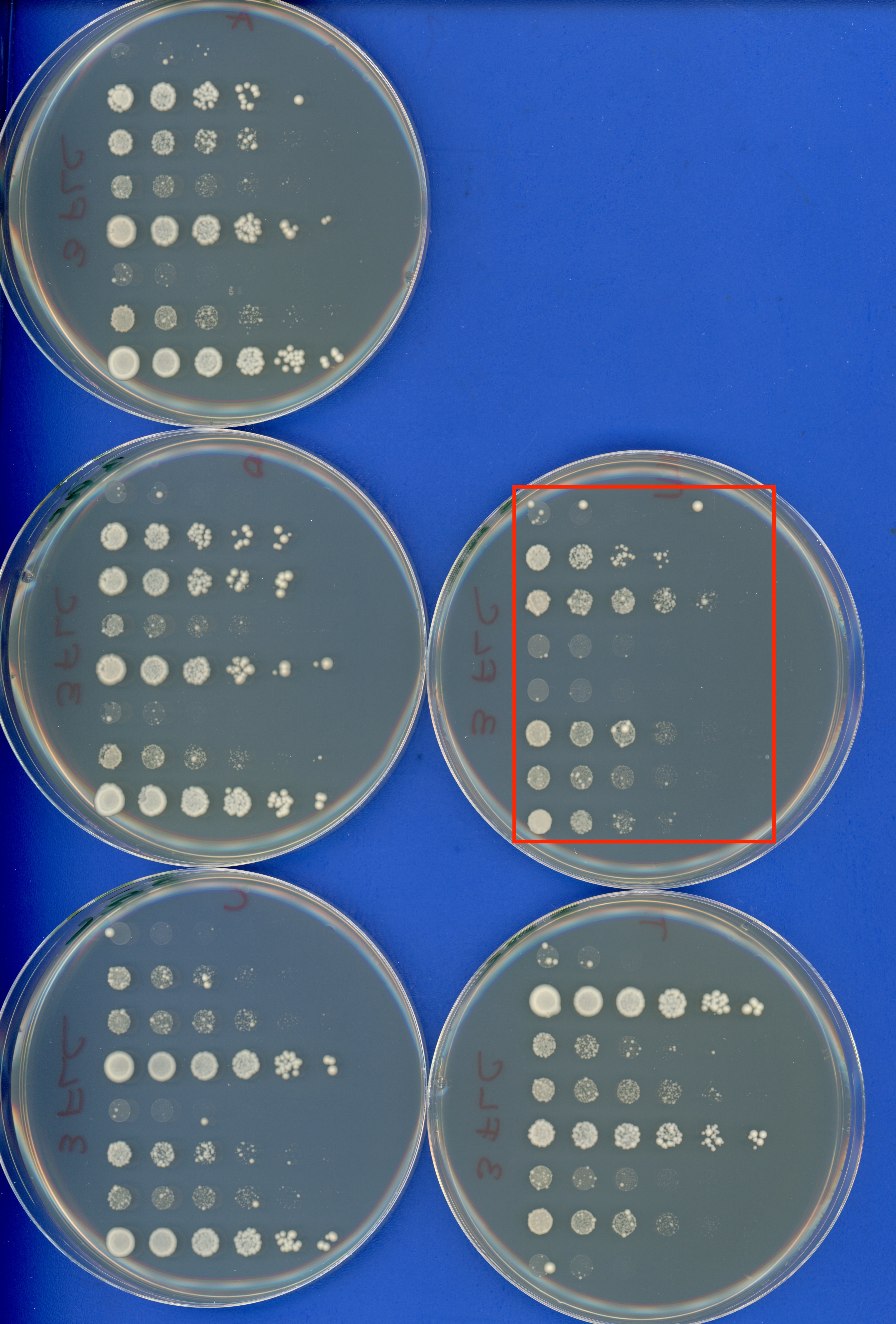

Supplement: Supplementary file 6 — Source data Fig. 2 [file 44318_2025_649_MOESM6_ESM.zip › 121174_Source_Data_Fig_2/Fig_2B/Fig2B_FLC_annotated.jpg]

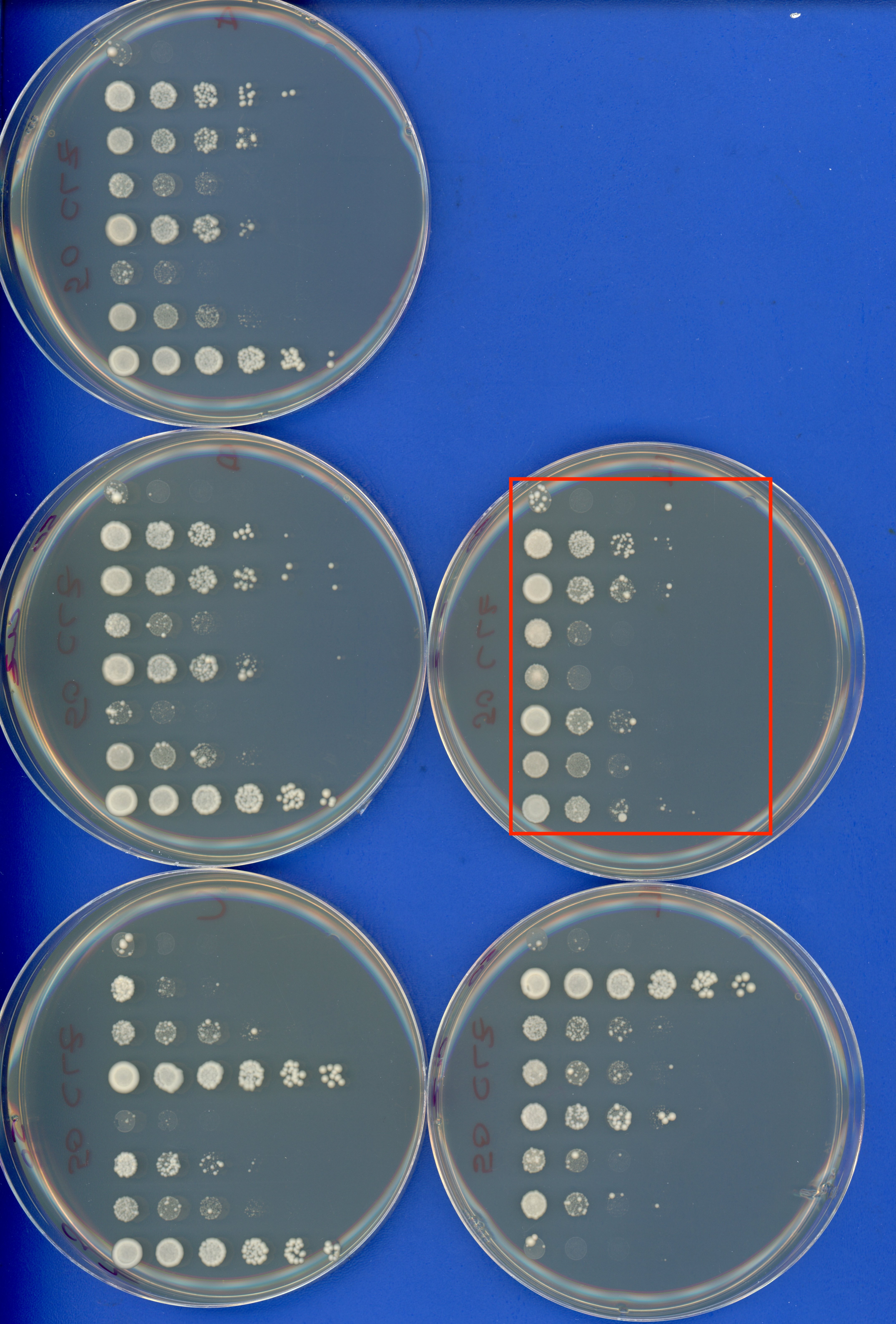

Supplement: Supplementary file 6 — Source data Fig. 2 [file 44318_2025_649_MOESM6_ESM.zip › 121174_Source_Data_Fig_2/Fig_2B/Fig2B_CLT_annotated.jpg]

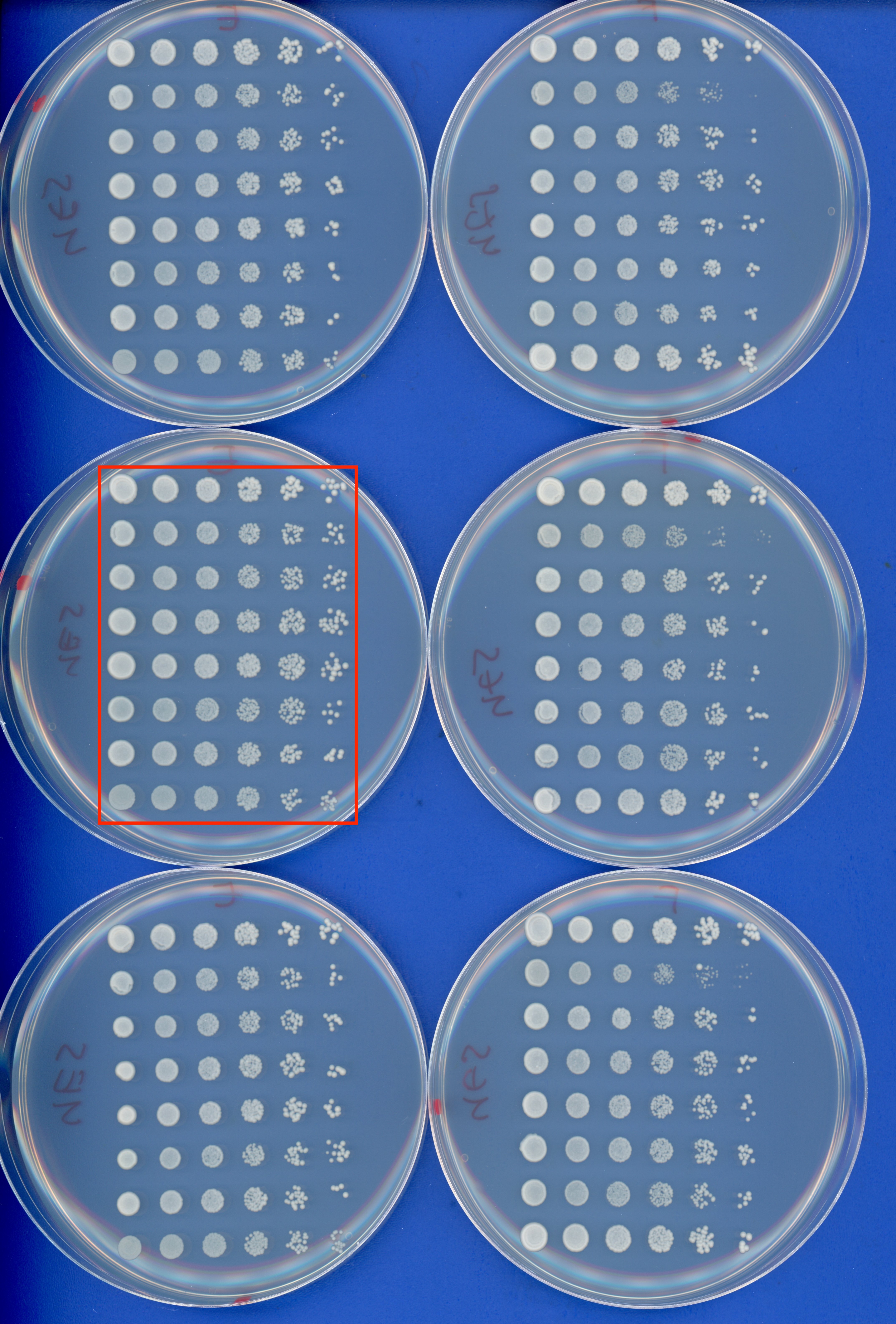

Supplement: Supplementary file 6 — Source data Fig. 2 [file 44318_2025_649_MOESM6_ESM.zip › 121174_Source_Data_Fig_2/Fig_2B/Fig2B_YES_annotated.jpg]

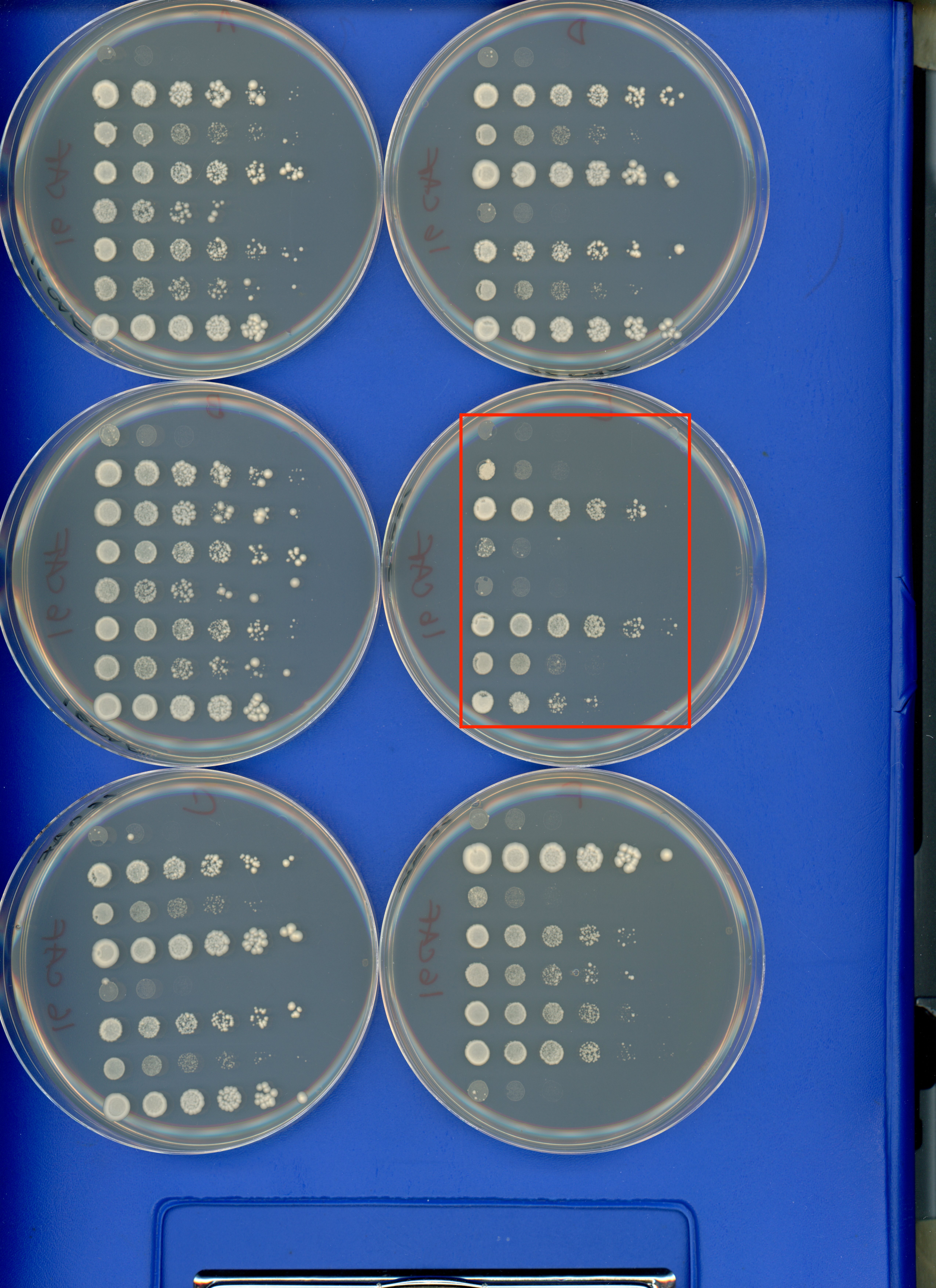

Supplement: Supplementary file 6 — Source data Fig. 2 [file 44318_2025_649_MOESM6_ESM.zip › 121174_Source_Data_Fig_2/Fig_2B/Fig2B_16CAF_annotated.jpg]

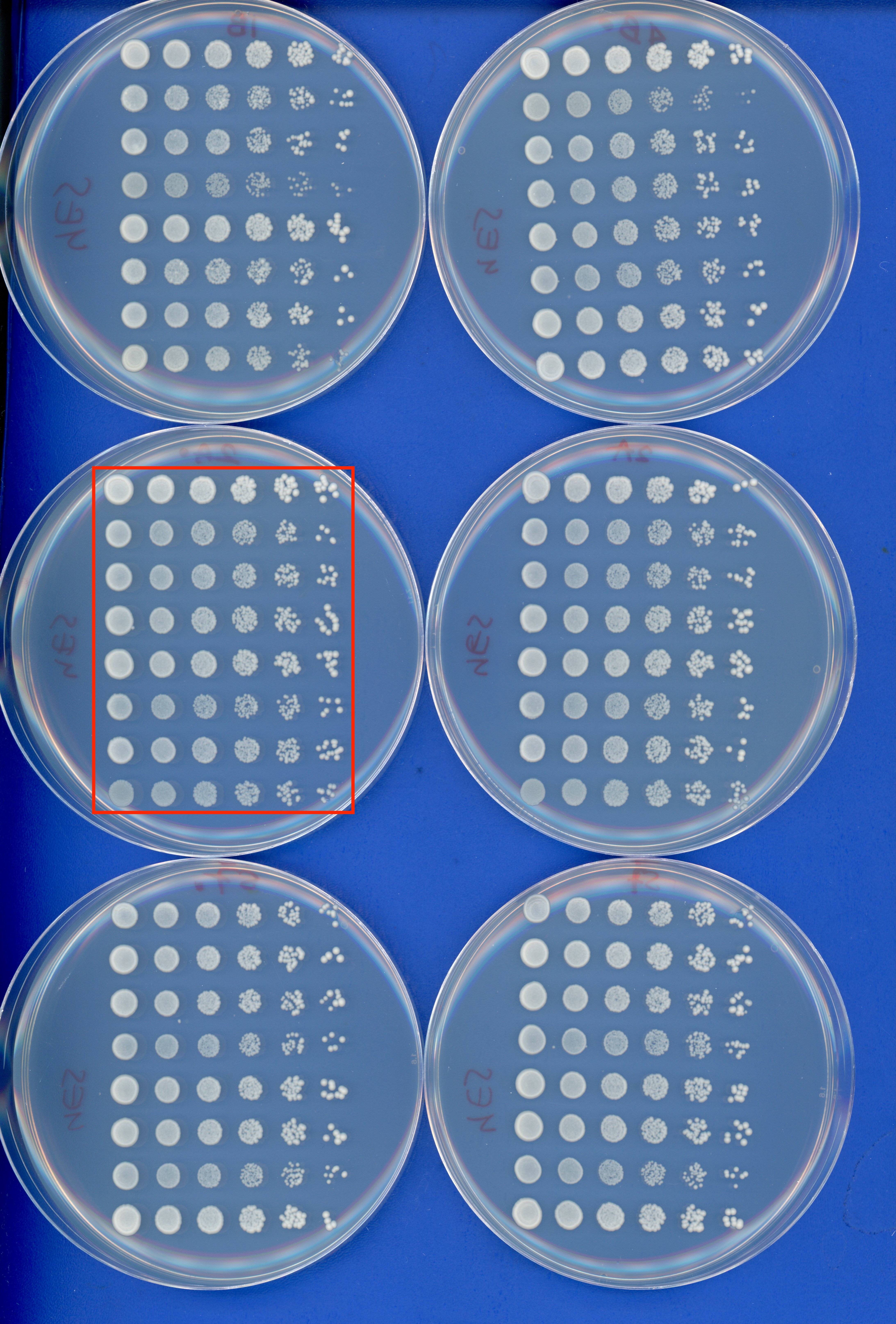

Supplement: Supplementary file 6 — Source data Fig. 2 [file 44318_2025_649_MOESM6_ESM.zip › 121174_Source_Data_Fig_2/Fig_2A/Fig2A_YES_annotated.jpg]

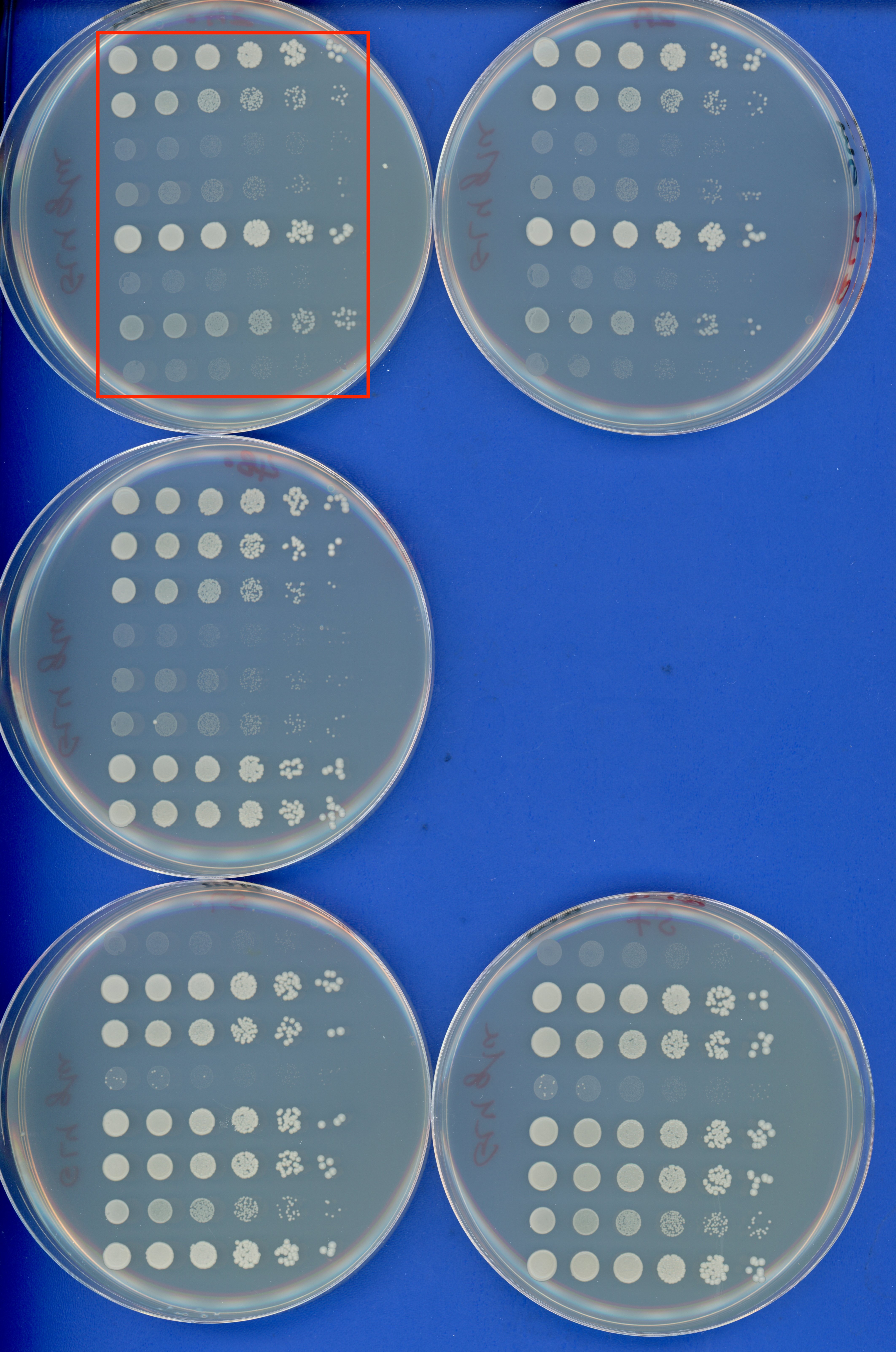

Supplement: Supplementary file 6 — Source data Fig. 2 [file 44318_2025_649_MOESM6_ESM.zip › 121174_Source_Data_Fig_2/Fig_2A/Fig2A_GLYglu_annotated.jpg]

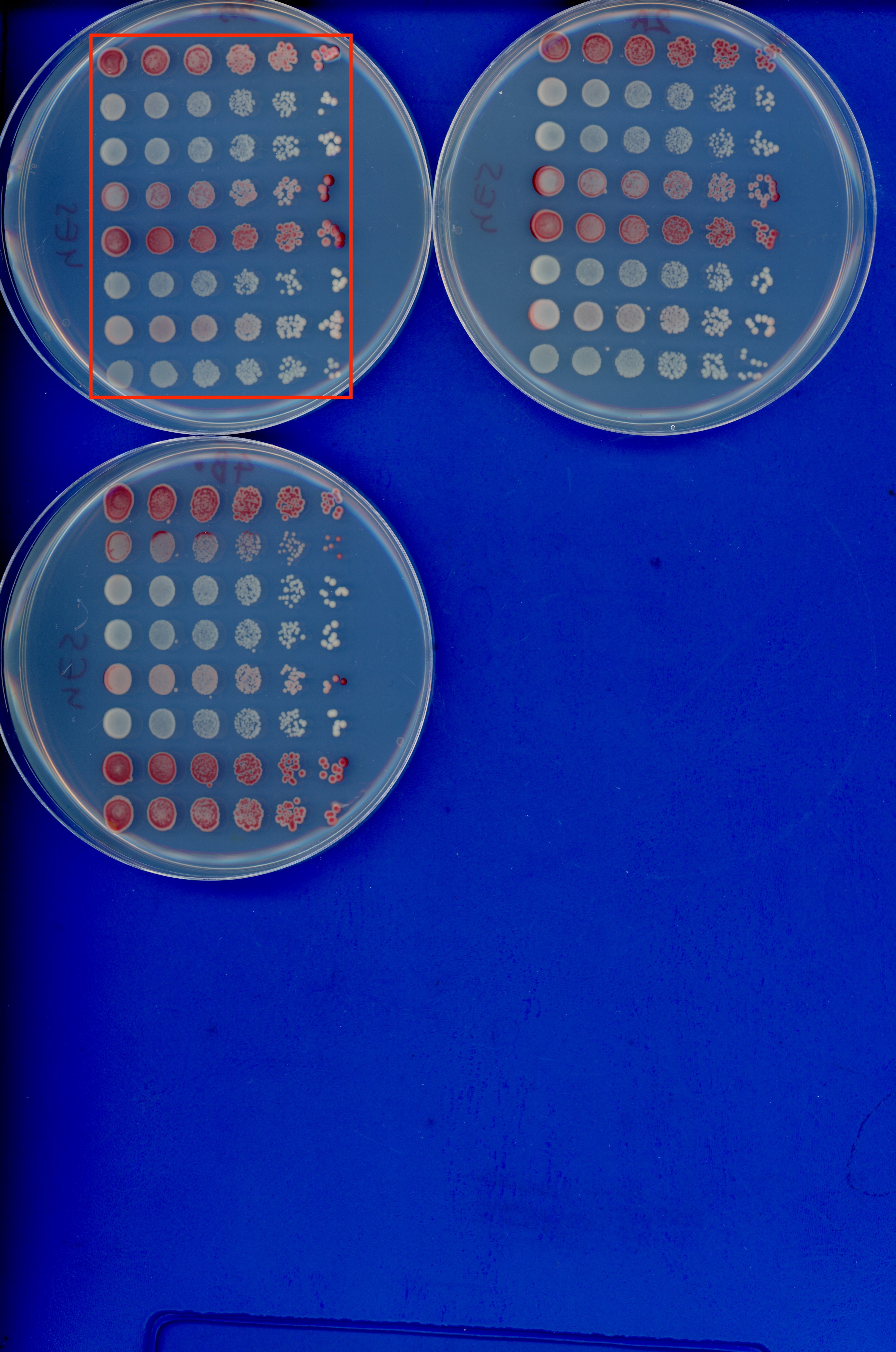

Supplement: Supplementary file 6 — Source data Fig. 2 [file 44318_2025_649_MOESM6_ESM.zip › 121174_Source_Data_Fig_2/Fig_2A/Fig2A_TTC_annotated.jpg]

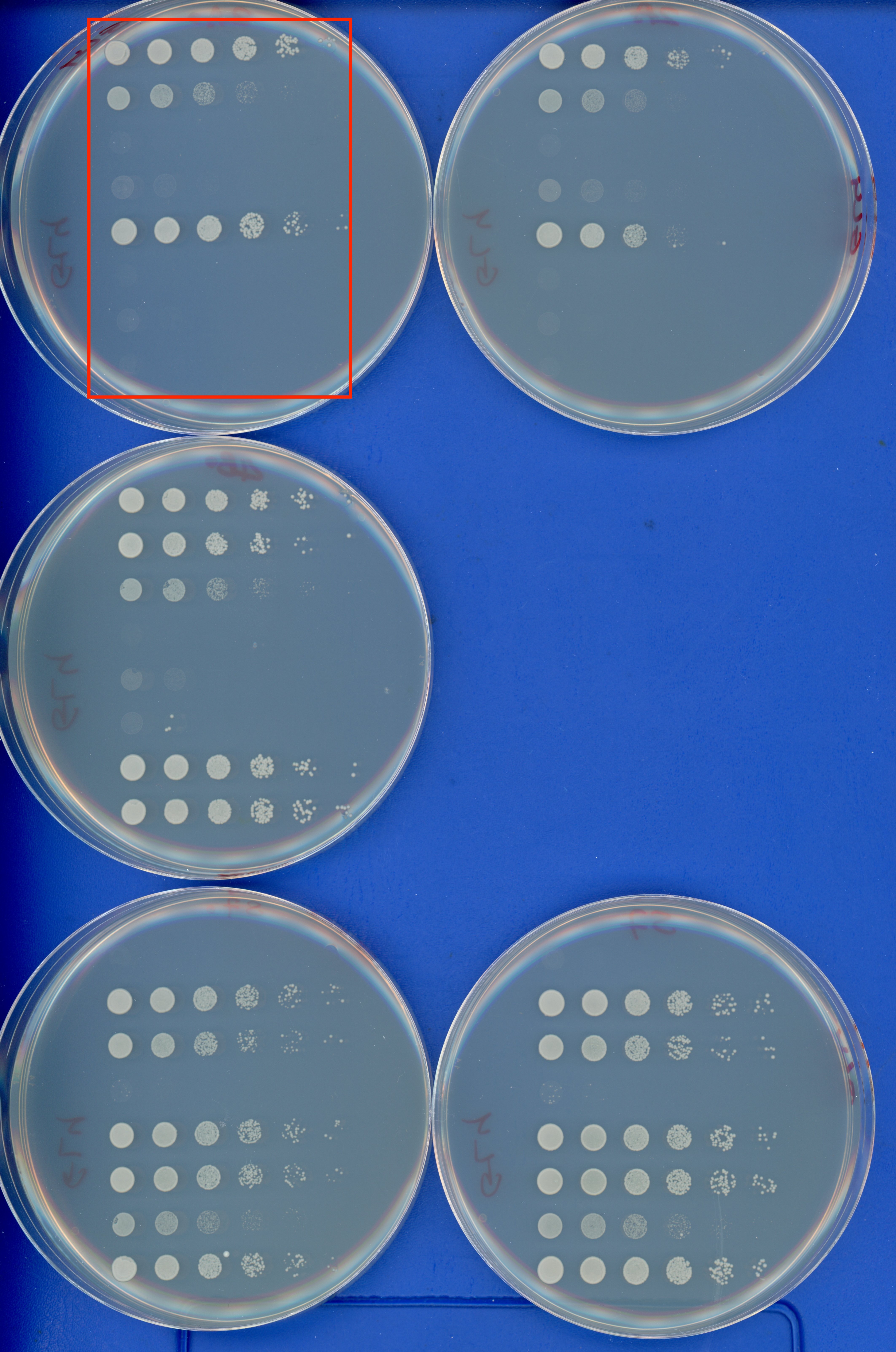

Supplement: Supplementary file 6 — Source data Fig. 2 [file 44318_2025_649_MOESM6_ESM.zip › 121174_Source_Data_Fig_2/Fig_2A/Fig2A_GLY_annotated.jpg]

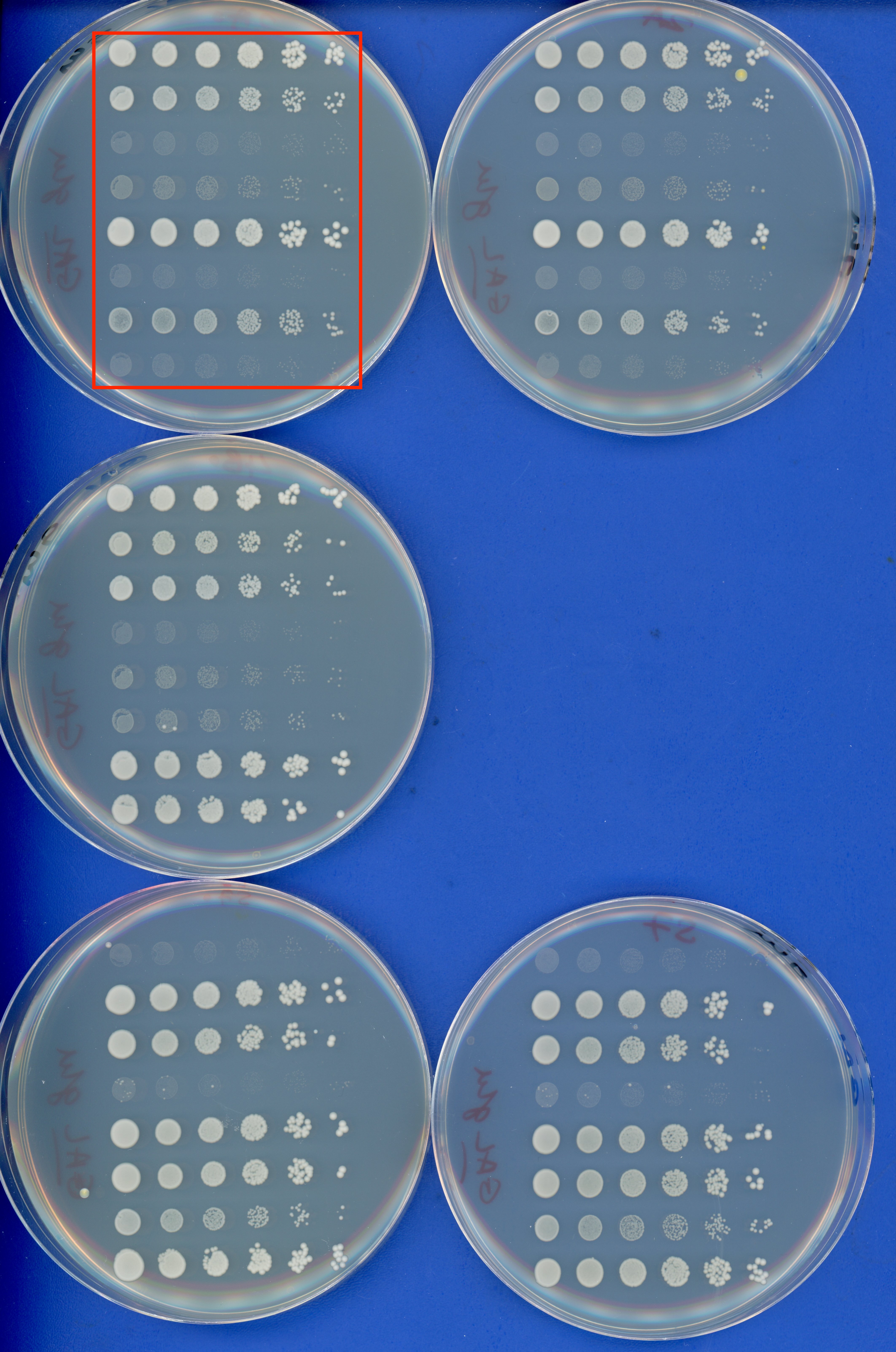

Supplement: Supplementary file 6 — Source data Fig. 2 [file 44318_2025_649_MOESM6_ESM.zip › 121174_Source_Data_Fig_2/Fig_2A/Fig2A_GALglu_annotated.jpg]

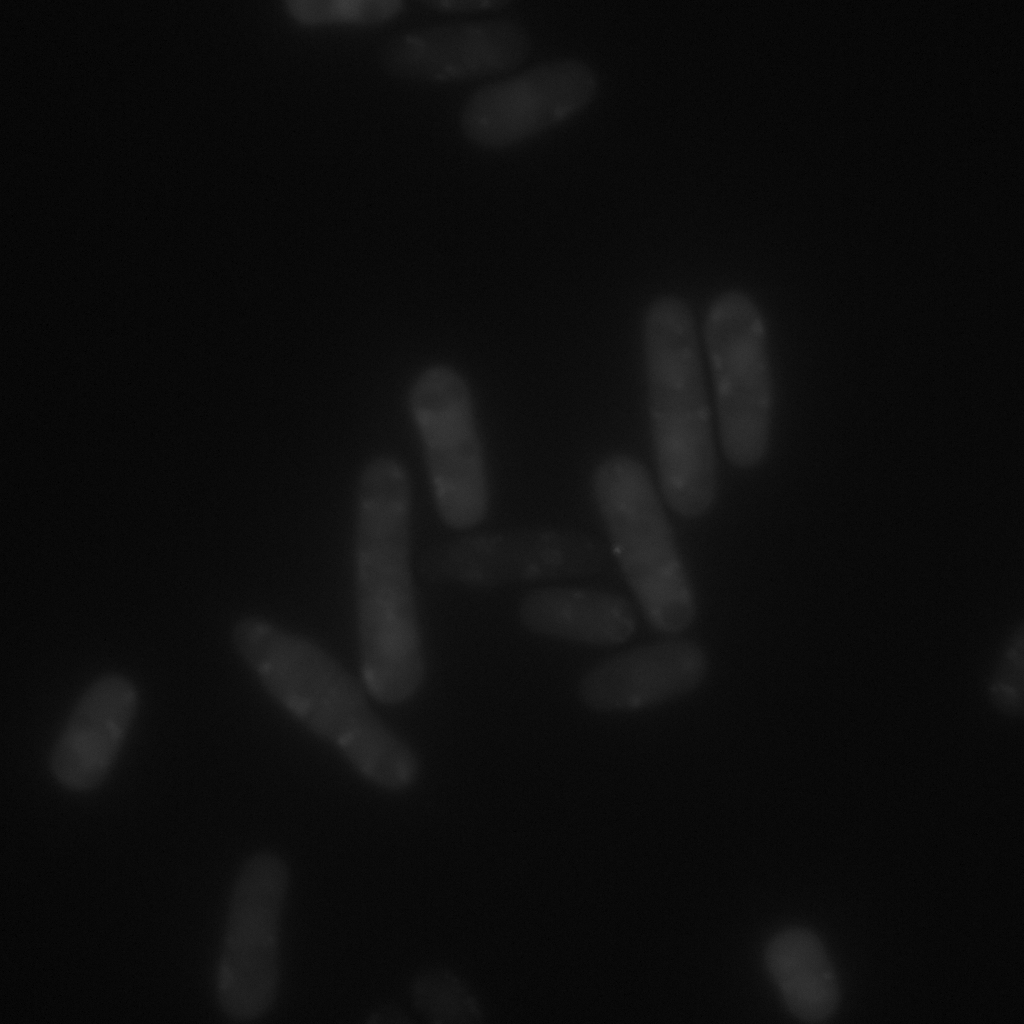

Supplement: Supplementary file 6 — Source data Fig. 2 [file 44318_2025_649_MOESM6_ESM.zip › 121174_Source_Data_Fig_2/Fig_2C/Fig2C_original_TIF_files/DCF_972_wt_002_w2FITC.TIF]

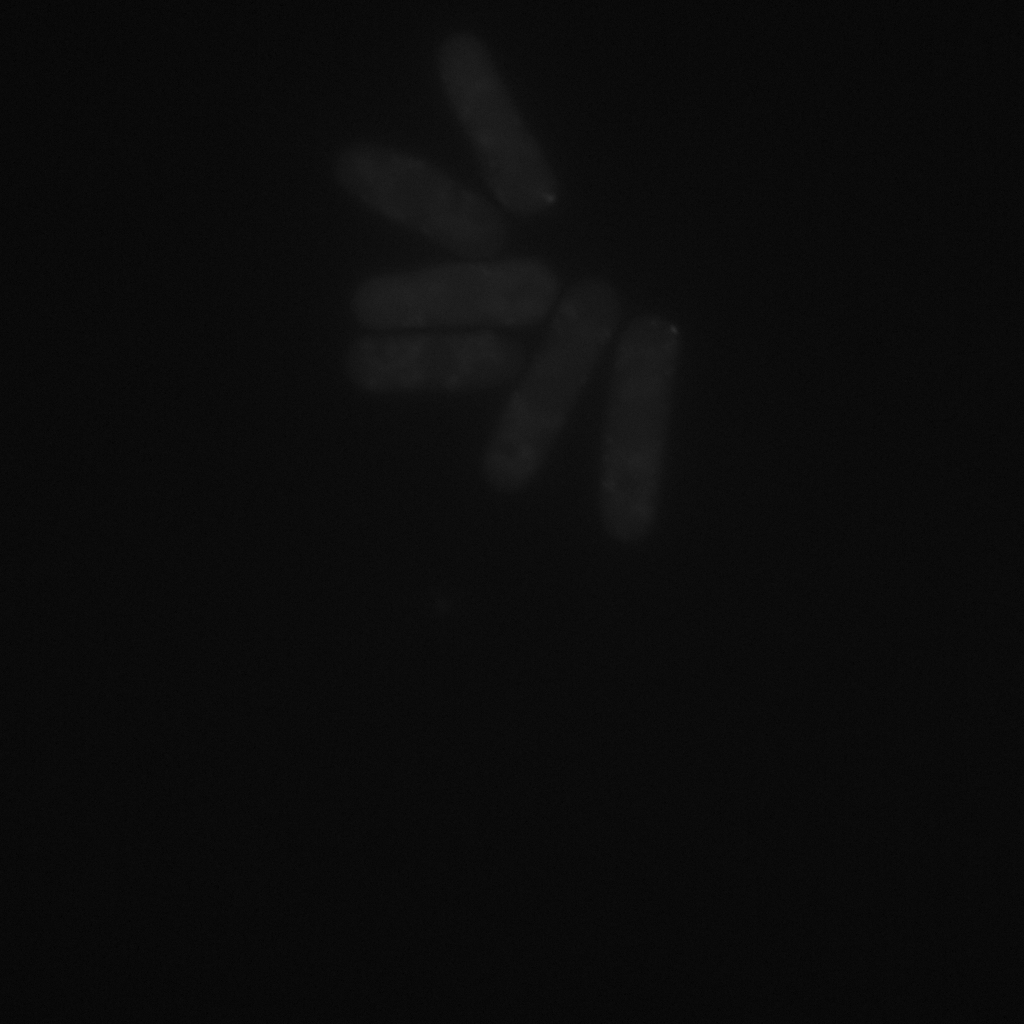

Supplement: Supplementary file 6 — Source data Fig. 2 [file 44318_2025_649_MOESM6_ESM.zip › 121174_Source_Data_Fig_2/Fig_2C/Fig2C_original_TIF_files/DCF_B6831_hba1_004_w2FITC.TIF]

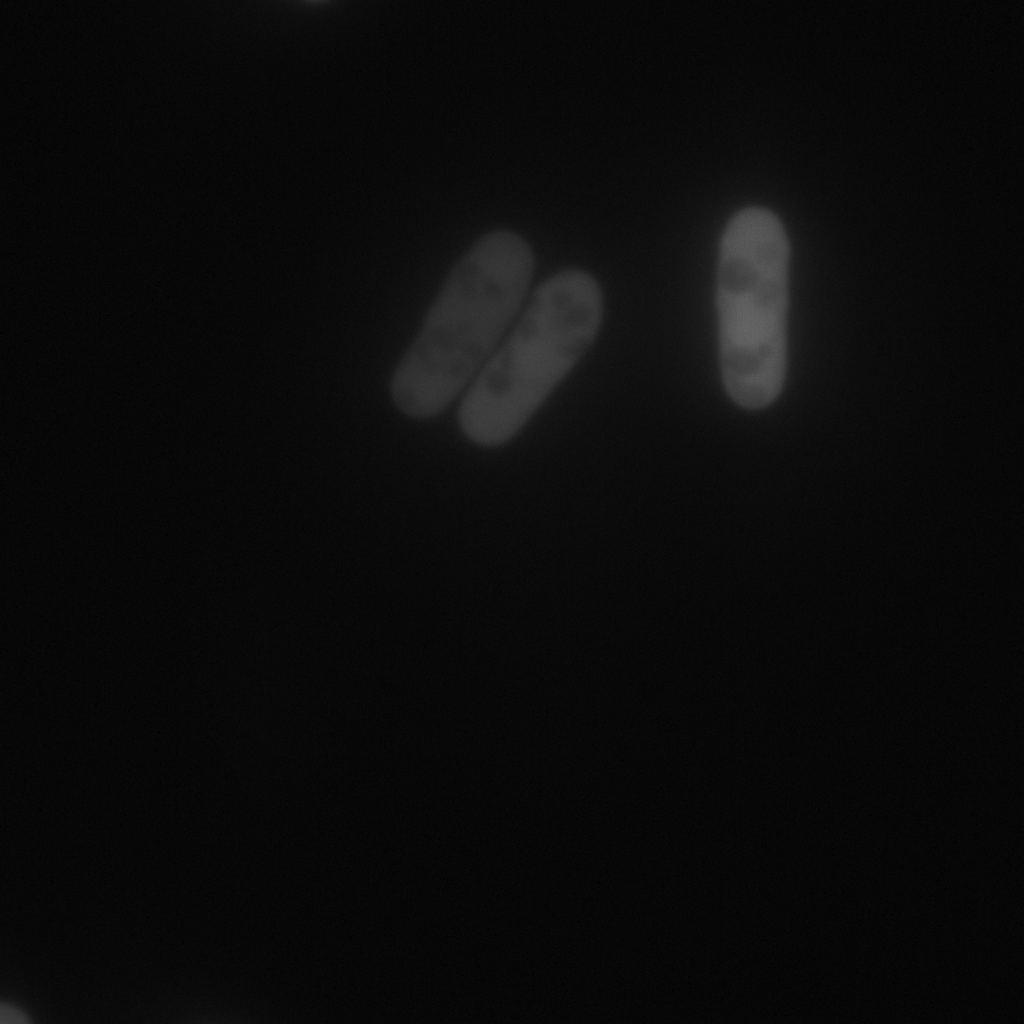

Supplement: Supplementary file 6 — Source data Fig. 2 [file 44318_2025_649_MOESM6_ESM.zip › 121174_Source_Data_Fig_2/Fig_2C/Fig2C_original_TIF_files/DCF_B7604_ndi1_001_w2FITC.TIF]

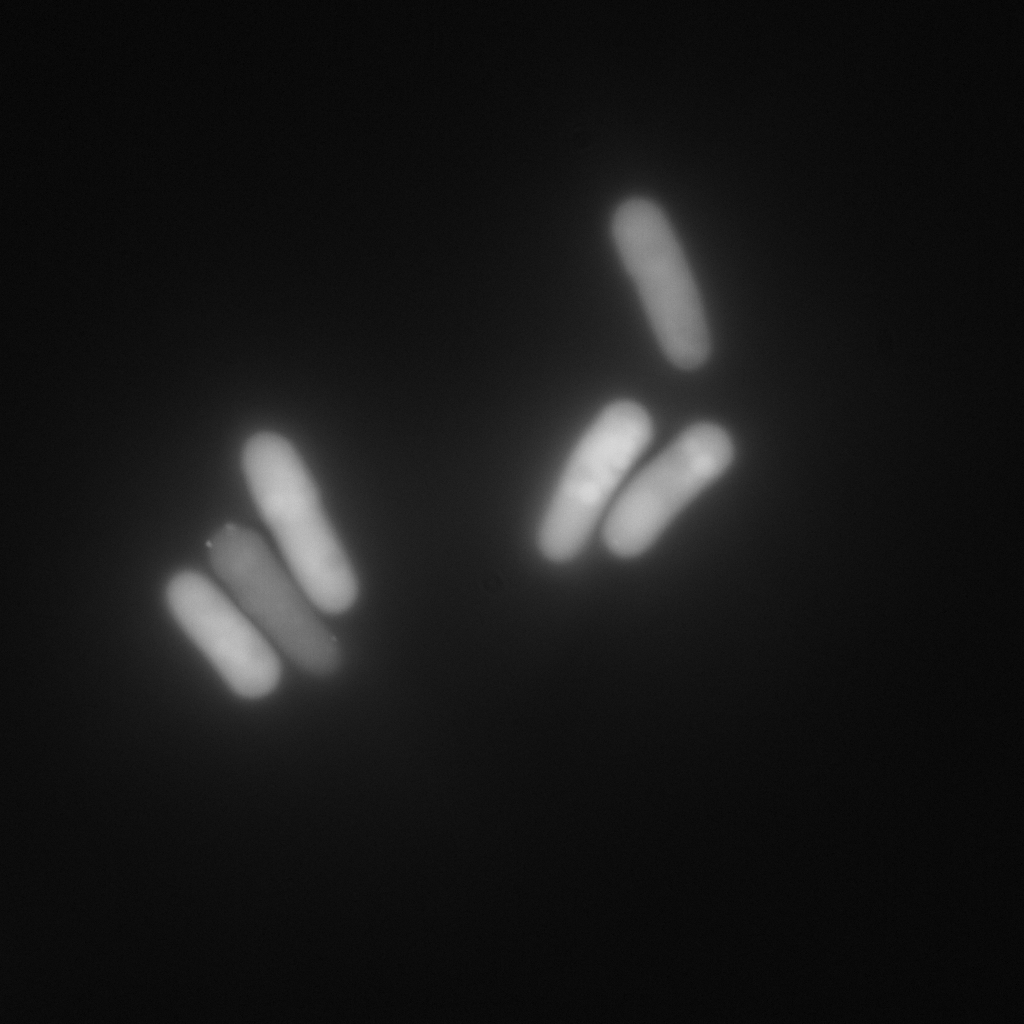

Supplement: Supplementary file 6 — Source data Fig. 2 [file 44318_2025_649_MOESM6_ESM.zip › 121174_Source_Data_Fig_2/Fig_2C/Fig2C_original_TIF_files/DCF_B6800_ppr4_001_w2FITC.TIF]

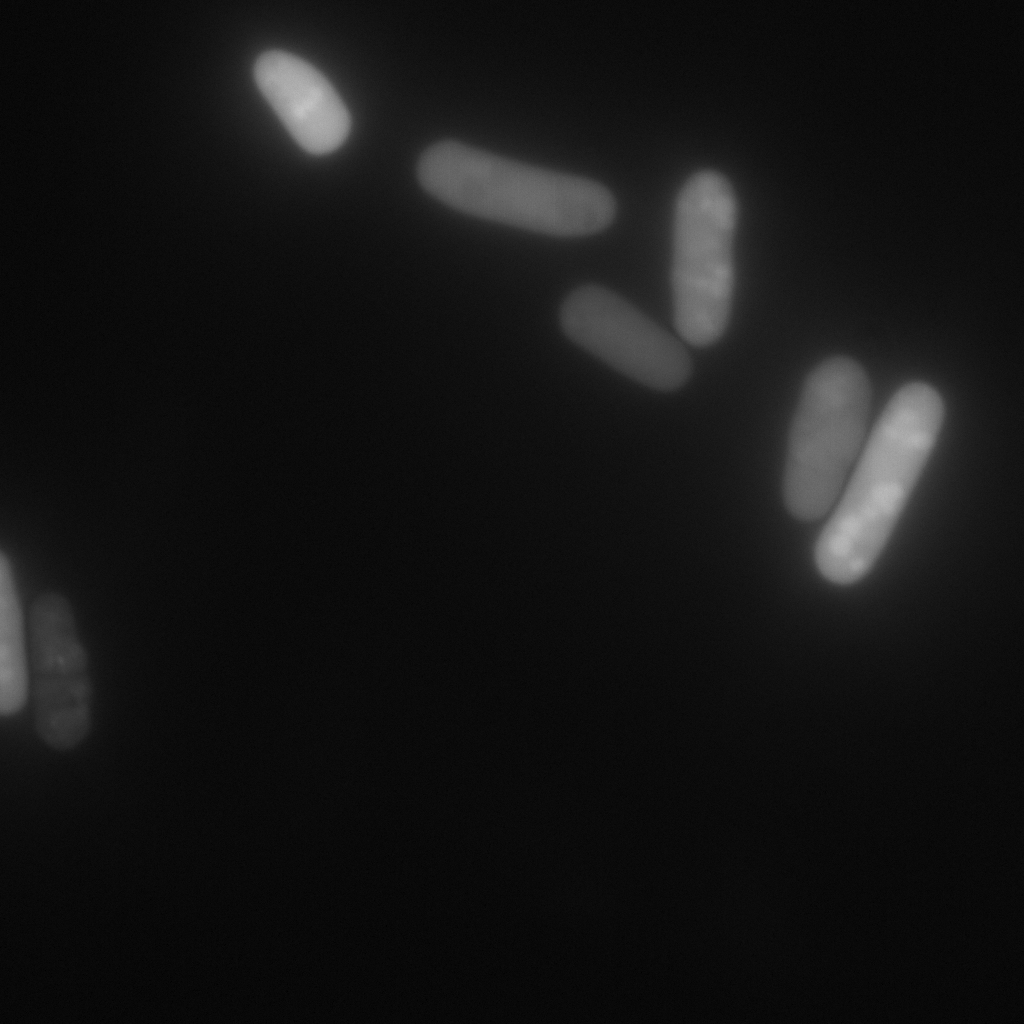

Supplement: Supplementary file 6 — Source data Fig. 2 [file 44318_2025_649_MOESM6_ESM.zip › 121174_Source_Data_Fig_2/Fig_2C/Fig2C_original_TIF_files/DCF_B6766_cup1_003_w2FITC.TIF]

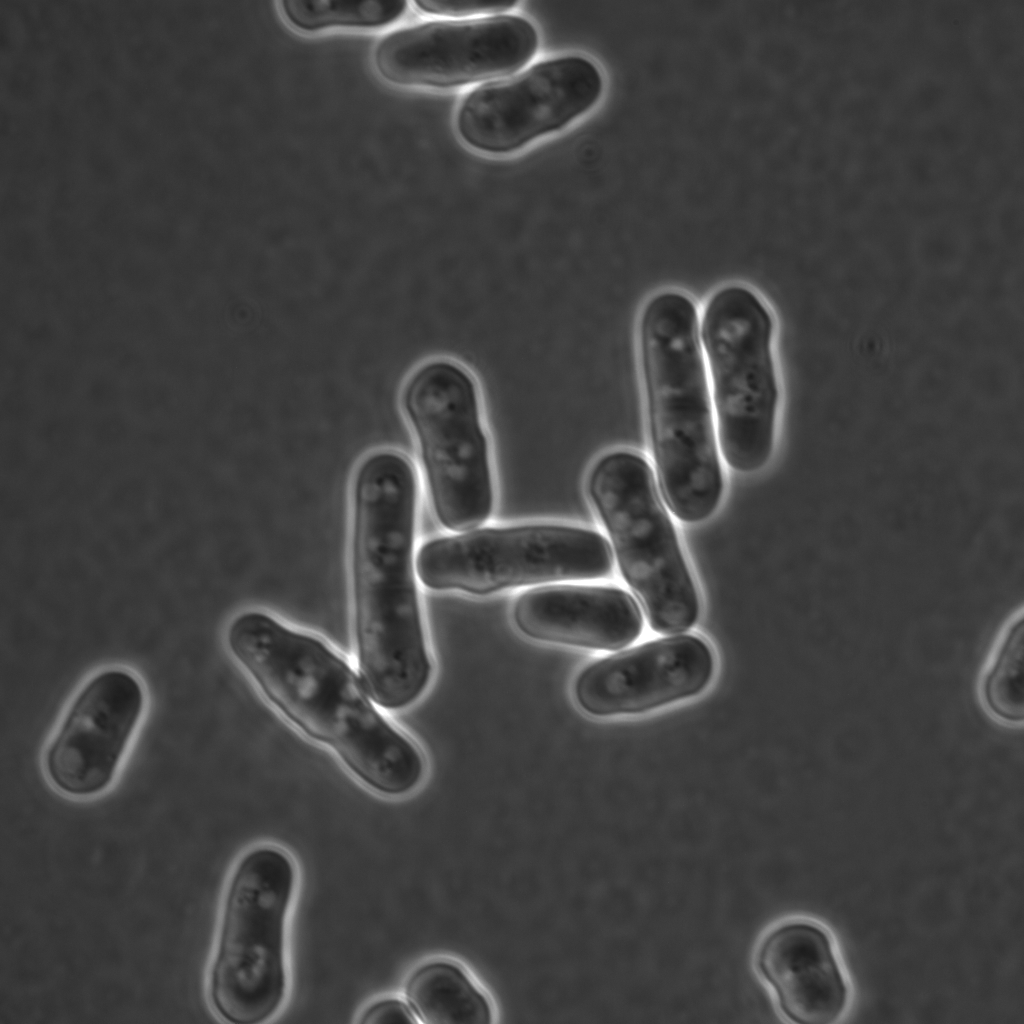

Supplement: Supplementary file 6 — Source data Fig. 2 [file 44318_2025_649_MOESM6_ESM.zip › 121174_Source_Data_Fig_2/Fig_2C/Fig2C_original_TIF_files/DCF_972_wt_002_w1DIC.TIF]

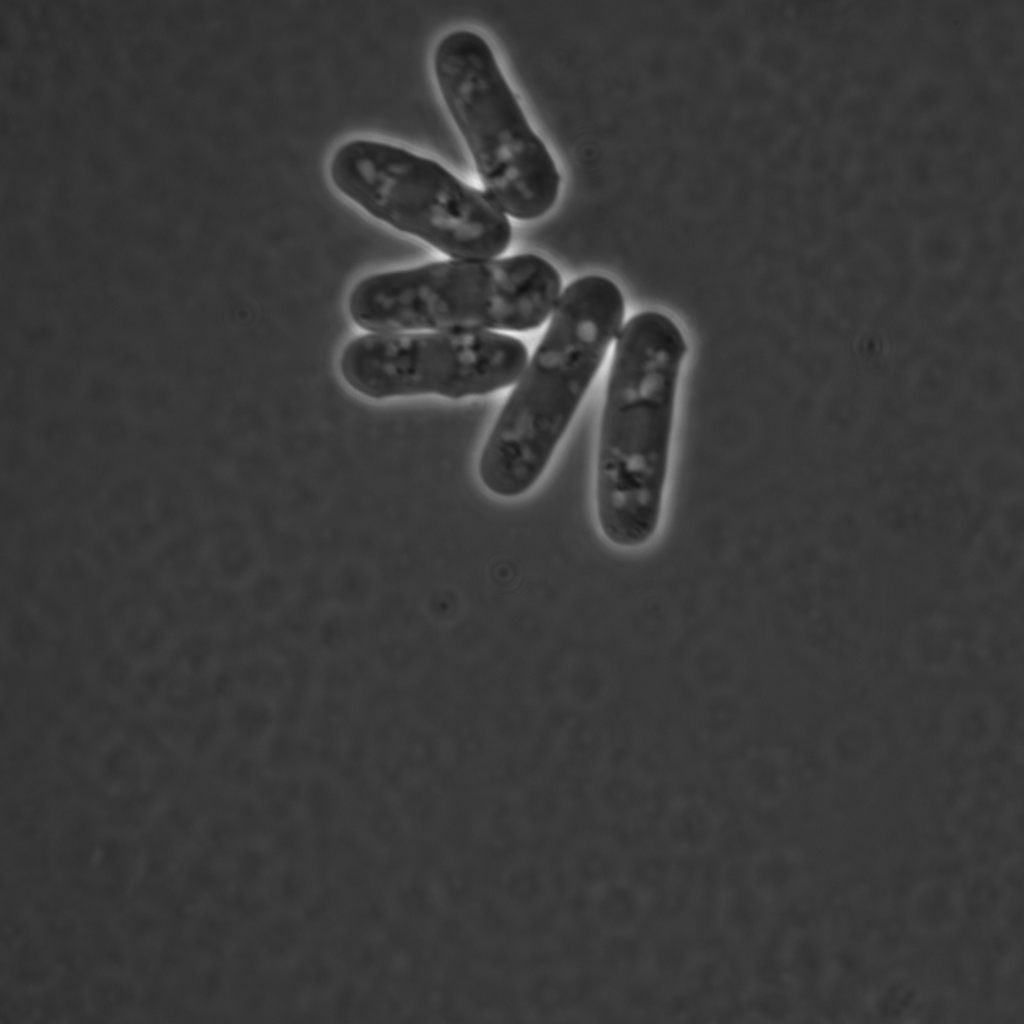

Supplement: Supplementary file 6 — Source data Fig. 2 [file 44318_2025_649_MOESM6_ESM.zip › 121174_Source_Data_Fig_2/Fig_2C/Fig2C_original_TIF_files/DCF_B6831_hba1_004_w1DIC.TIF]

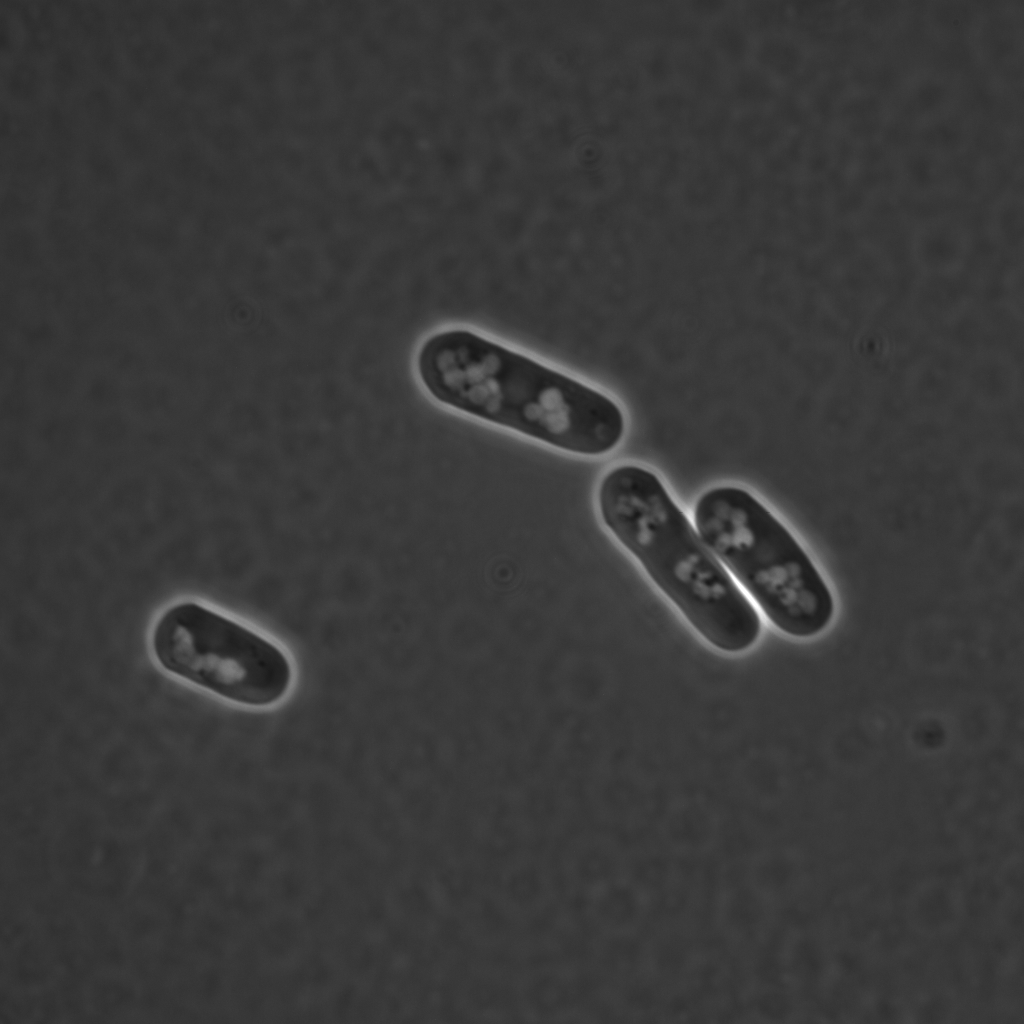

Supplement: Supplementary file 6 — Source data Fig. 2 [file 44318_2025_649_MOESM6_ESM.zip › 121174_Source_Data_Fig_2/Fig_2C/Fig2C_original_TIF_files/DCF_B7614_cox4_002_w1DIC.TIF]

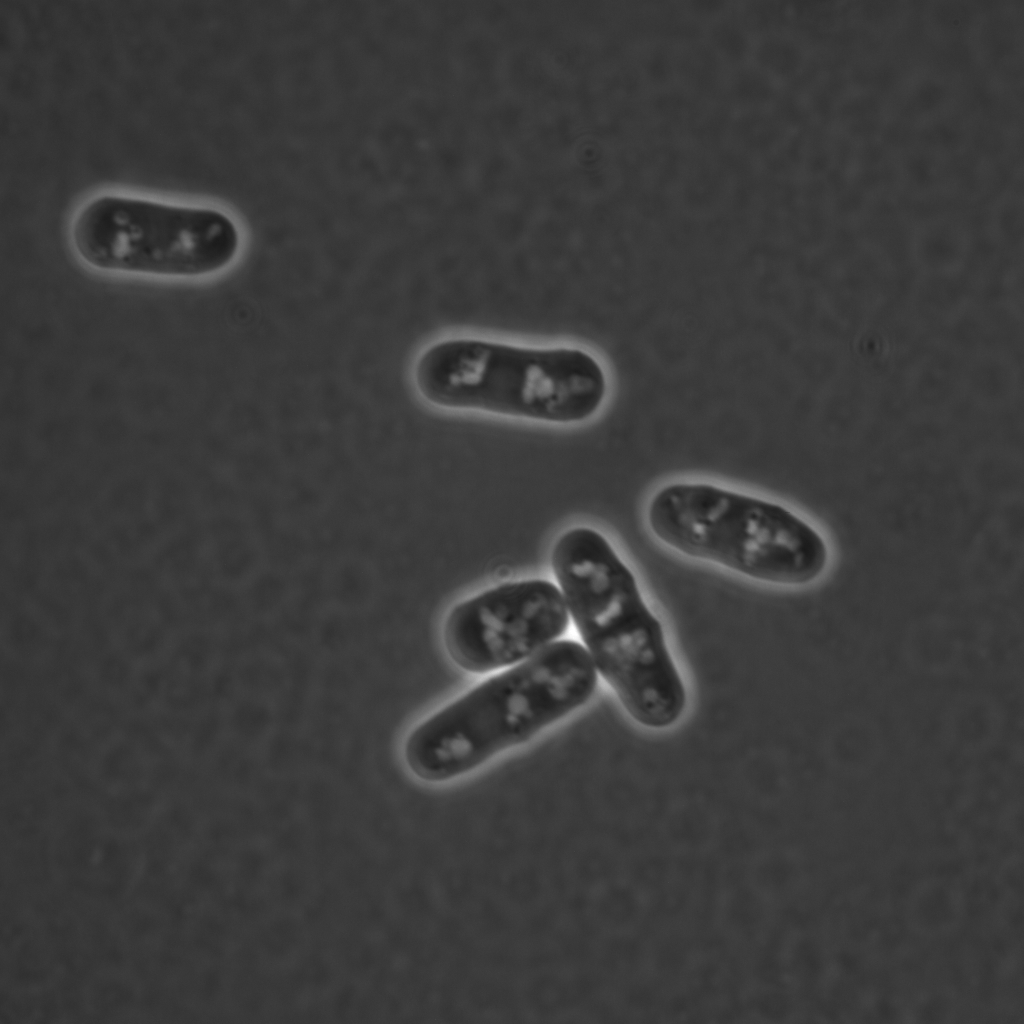

Supplement: Supplementary file 6 — Source data Fig. 2 [file 44318_2025_649_MOESM6_ESM.zip › 121174_Source_Data_Fig_2/Fig_2C/Fig2C_original_TIF_files/DCF_B7641_qcr7_001_w1DIC.TIF]

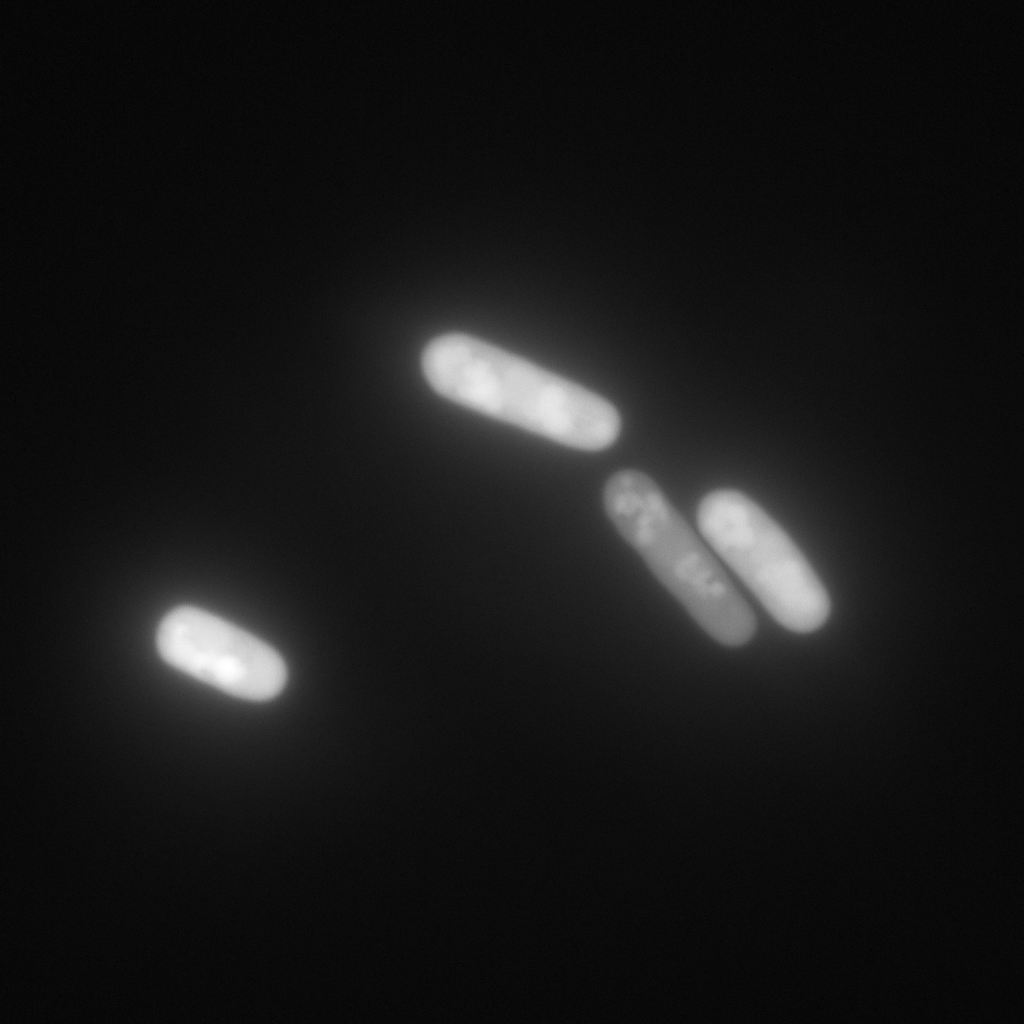

Supplement: Supplementary file 6 — Source data Fig. 2 [file 44318_2025_649_MOESM6_ESM.zip › 121174_Source_Data_Fig_2/Fig_2C/Fig2C_original_TIF_files/DCF_B7614_cox4_002_w2FITC.TIF]

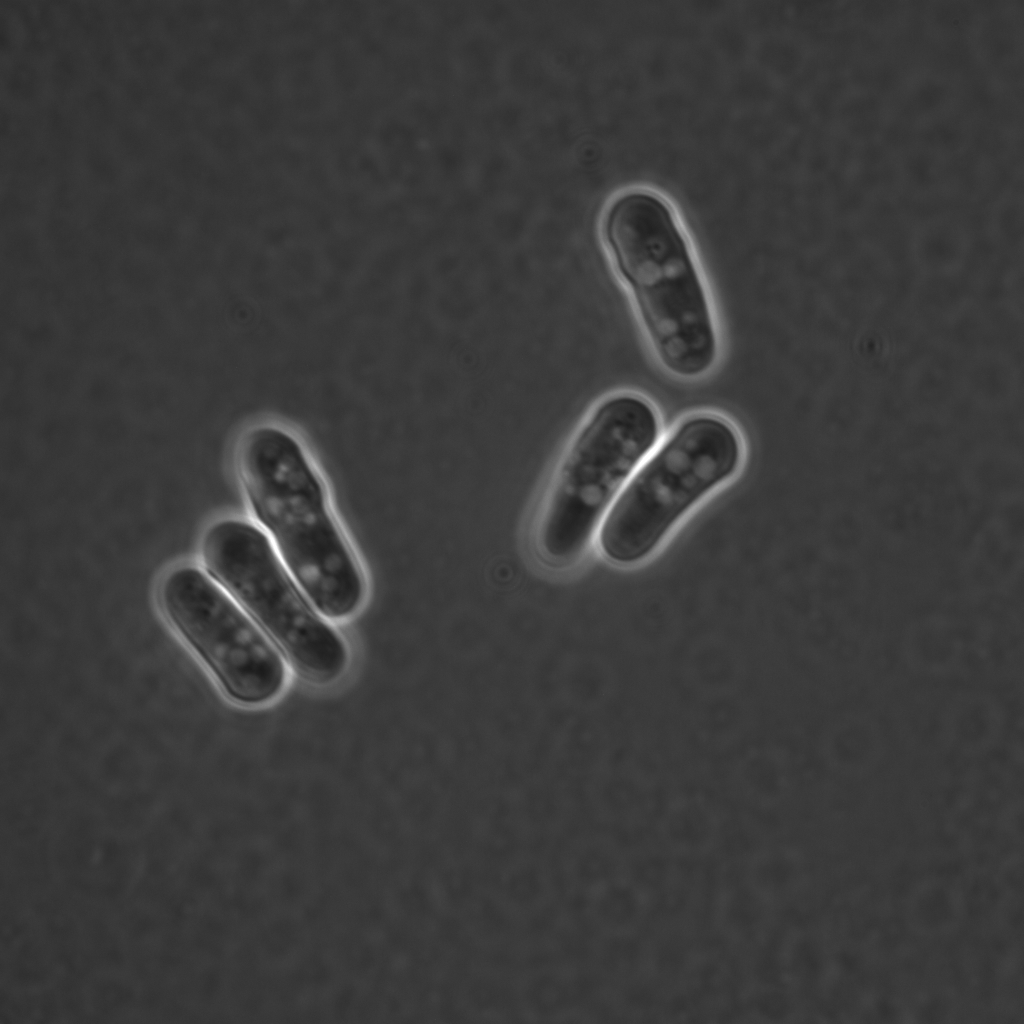

Supplement: Supplementary file 6 — Source data Fig. 2 [file 44318_2025_649_MOESM6_ESM.zip › 121174_Source_Data_Fig_2/Fig_2C/Fig2C_original_TIF_files/DCF_B6800_ppr4_001_w1DIC.TIF]

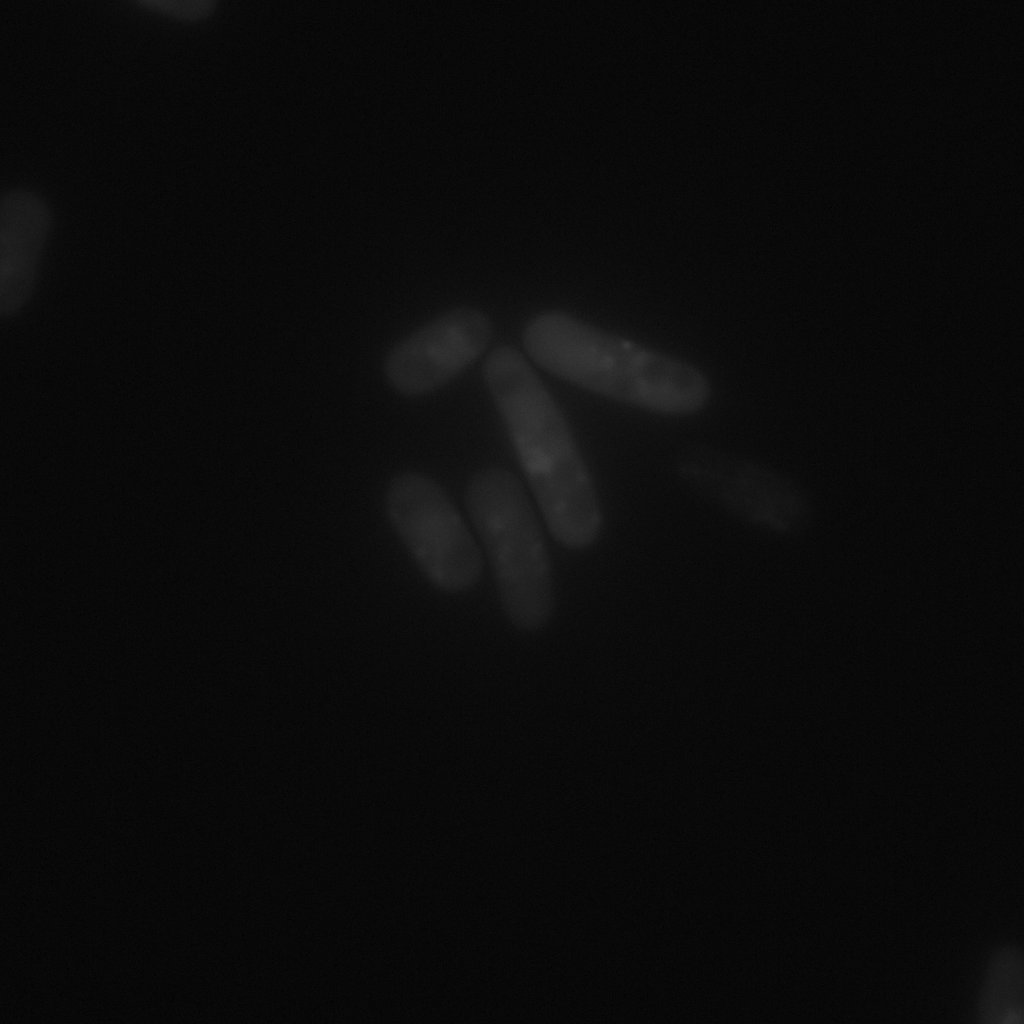

Supplement: Supplementary file 6 — Source data Fig. 2 [file 44318_2025_649_MOESM6_ESM.zip › 121174_Source_Data_Fig_2/Fig_2C/Fig2C_original_TIF_files/DCF_B7606_sdh7_005_w2FITC.TIF]

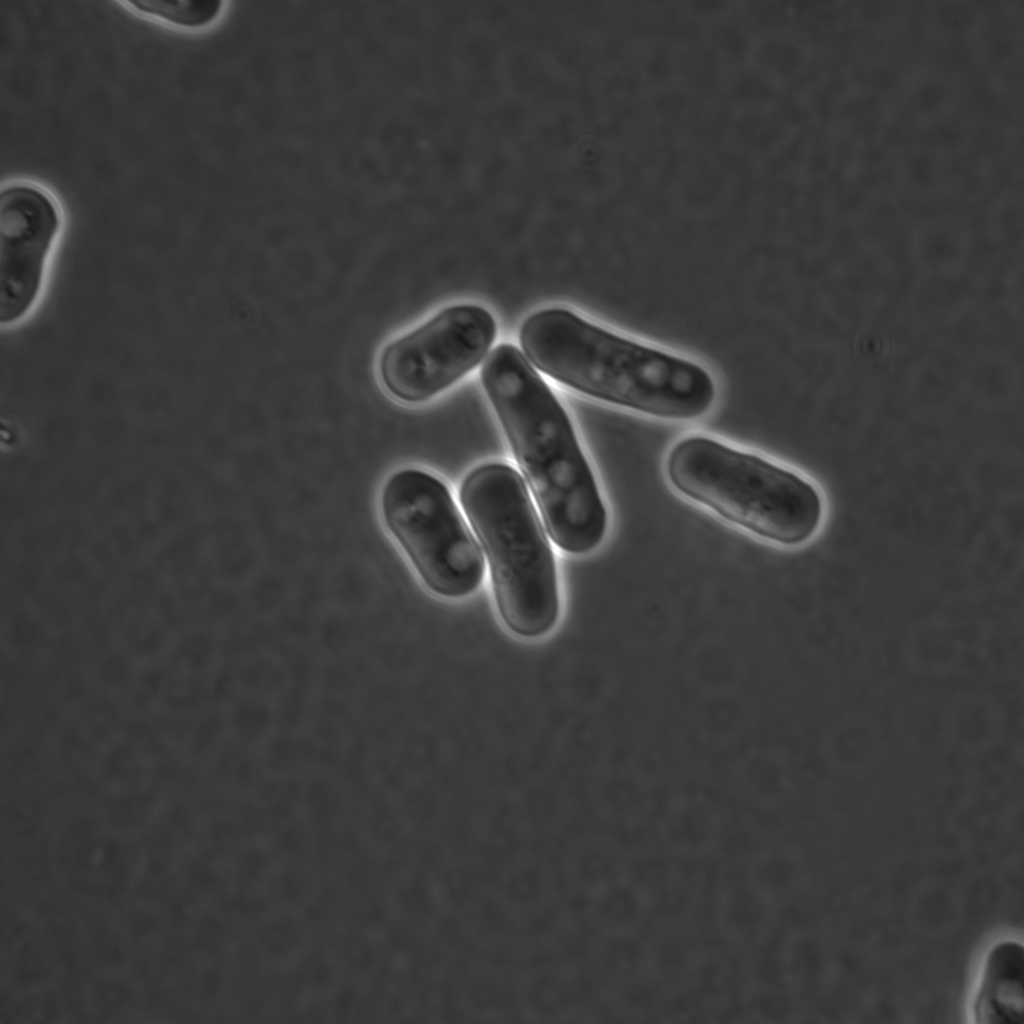

Supplement: Supplementary file 6 — Source data Fig. 2 [file 44318_2025_649_MOESM6_ESM.zip › 121174_Source_Data_Fig_2/Fig_2C/Fig2C_original_TIF_files/DCF_B7606_sdh7_005_w1DIC.TIF]

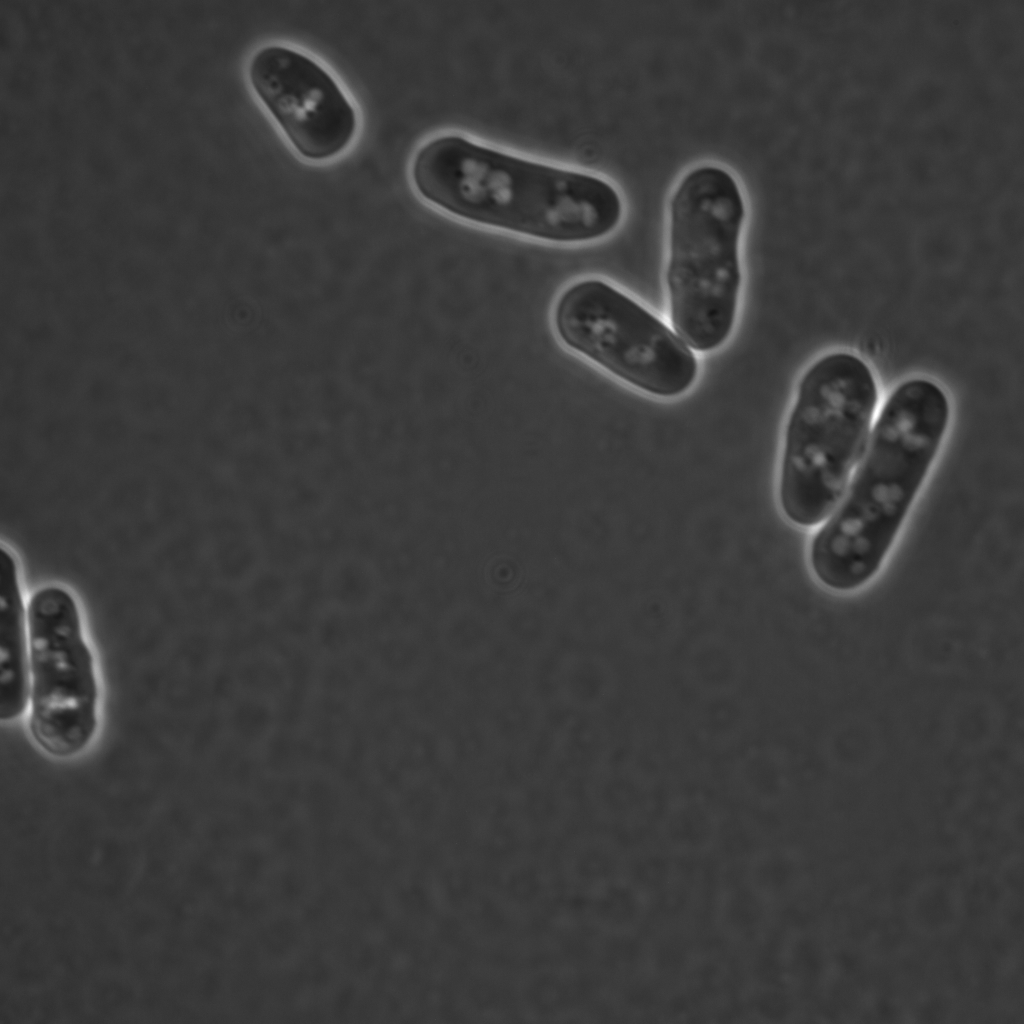

Supplement: Supplementary file 6 — Source data Fig. 2 [file 44318_2025_649_MOESM6_ESM.zip › 121174_Source_Data_Fig_2/Fig_2C/Fig2C_original_TIF_files/DCF_B6766_cup1_003_w1DIC.TIF]

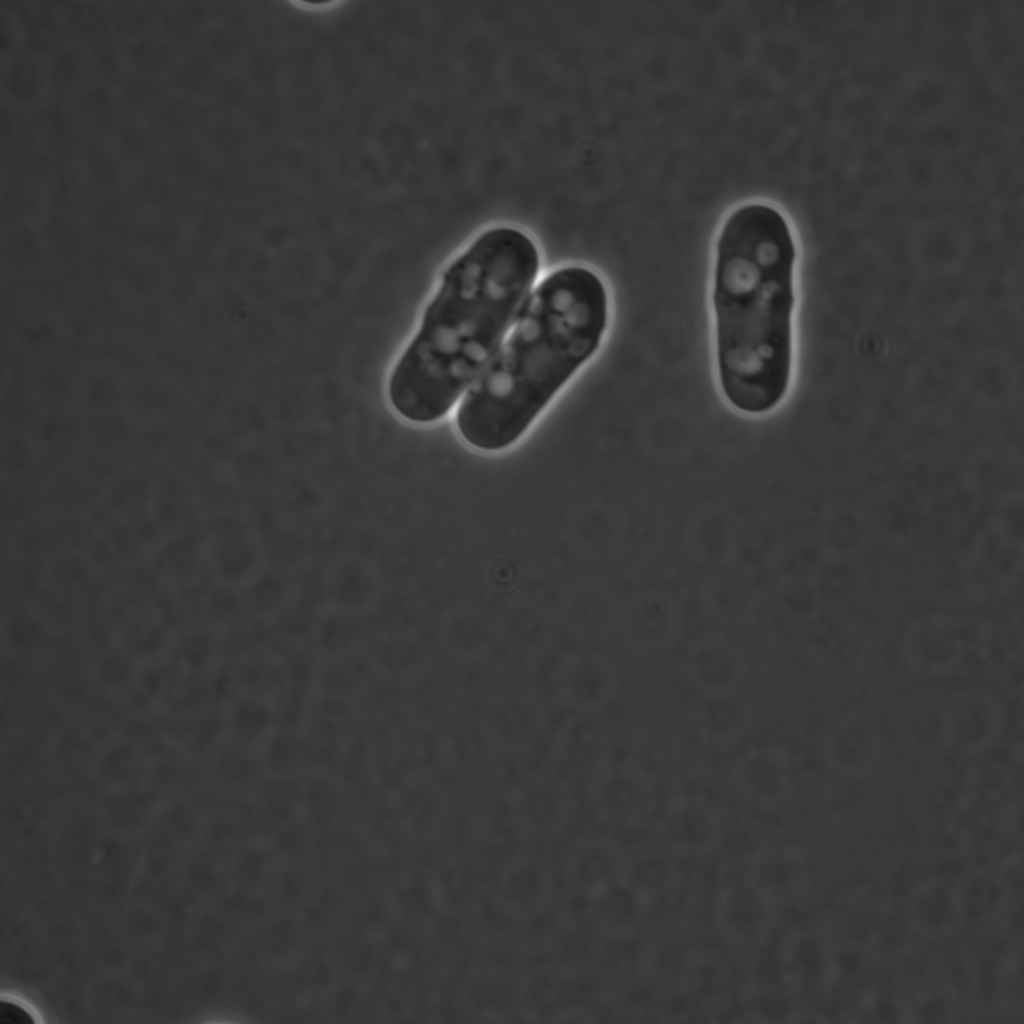

Supplement: Supplementary file 6 — Source data Fig. 2 [file 44318_2025_649_MOESM6_ESM.zip › 121174_Source_Data_Fig_2/Fig_2C/Fig2C_original_TIF_files/DCF_B7604_ndi1_001_w1DIC.TIF]

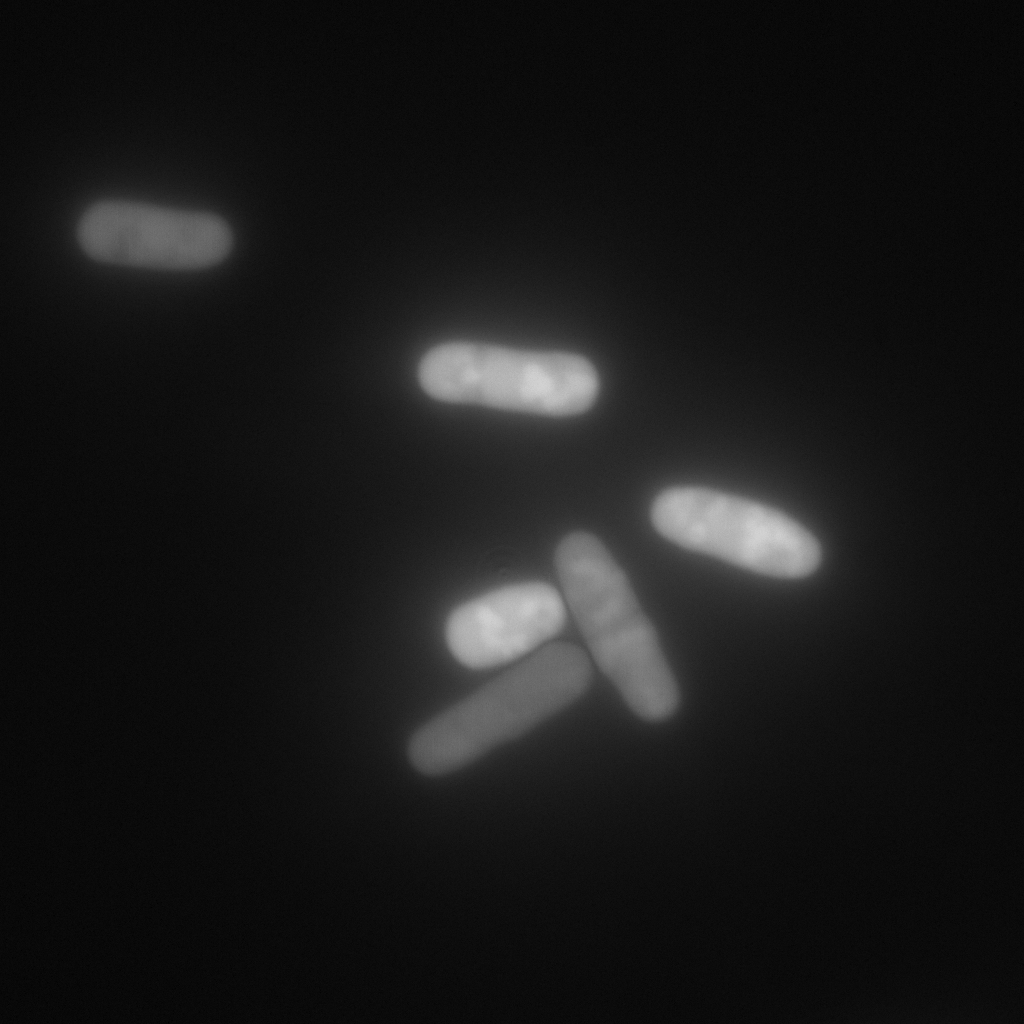

Supplement: Supplementary file 6 — Source data Fig. 2 [file 44318_2025_649_MOESM6_ESM.zip › 121174_Source_Data_Fig_2/Fig_2C/Fig2C_original_TIF_files/DCF_B7641_qcr7_001_w2FITC.TIF]

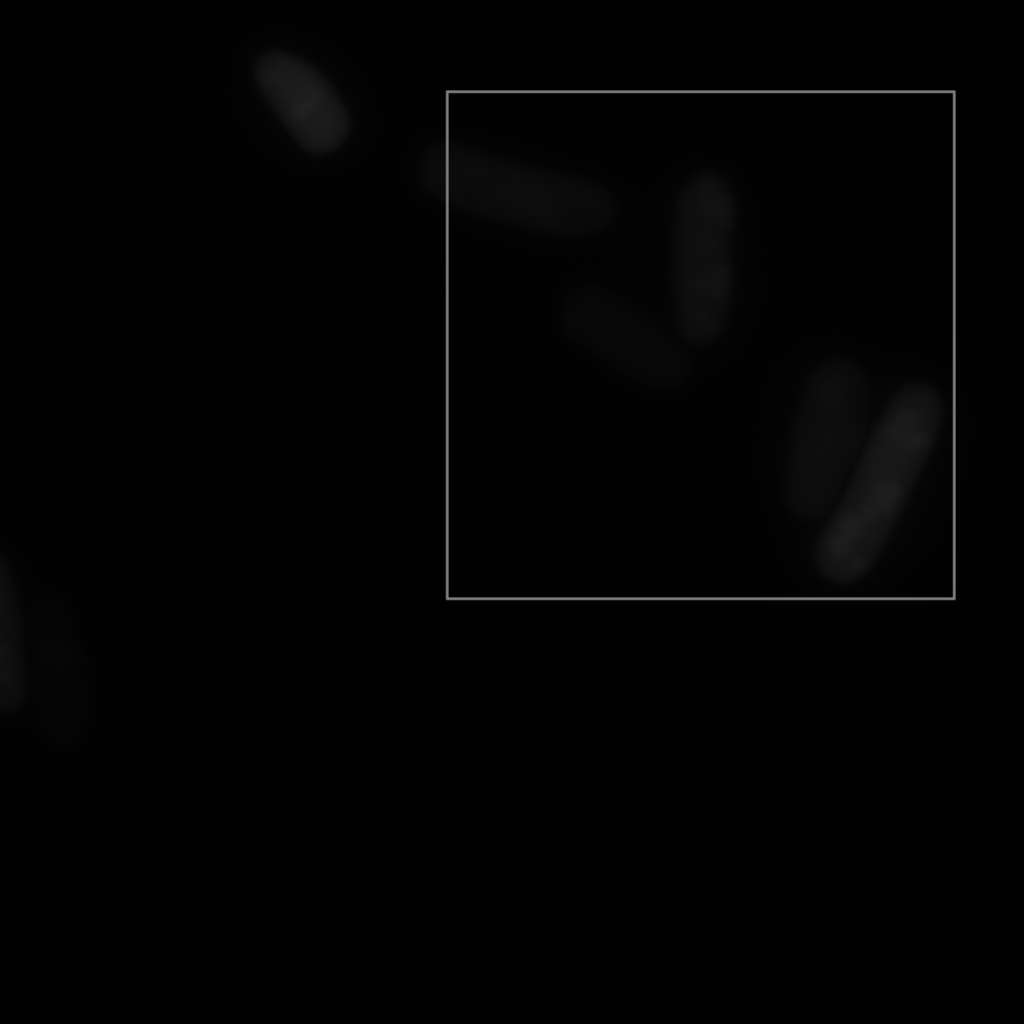

Supplement: Supplementary file 6 — Source data Fig. 2 [file 44318_2025_649_MOESM6_ESM.zip › 121174_Source_Data_Fig_2/Fig_2C/Fig_2C_annotated/Fellas_Fig_2C_DCF_cup1_annotated.TIF]

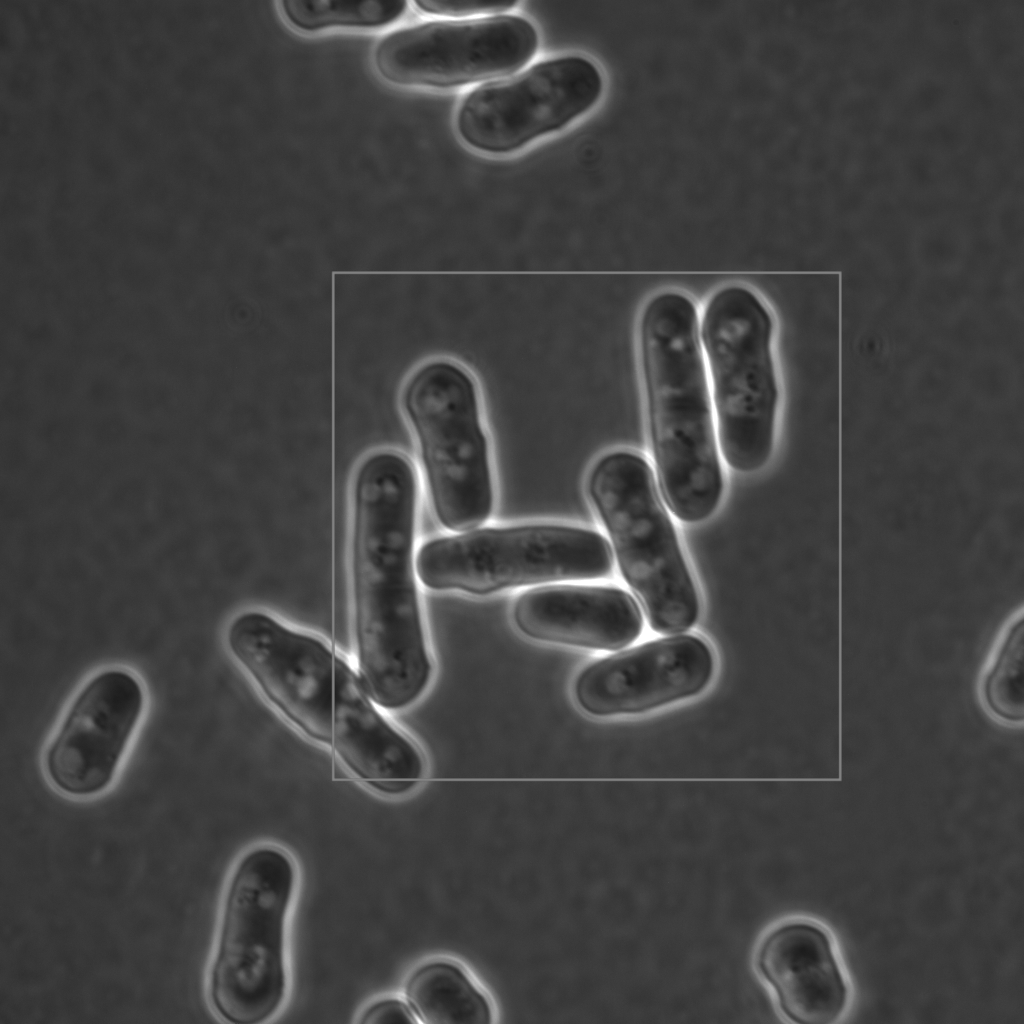

Supplement: Supplementary file 6 — Source data Fig. 2 [file 44318_2025_649_MOESM6_ESM.zip › 121174_Source_Data_Fig_2/Fig_2C/Fig_2C_annotated/Fellas_Fig_2C_BF_wt_annotated.TIF]

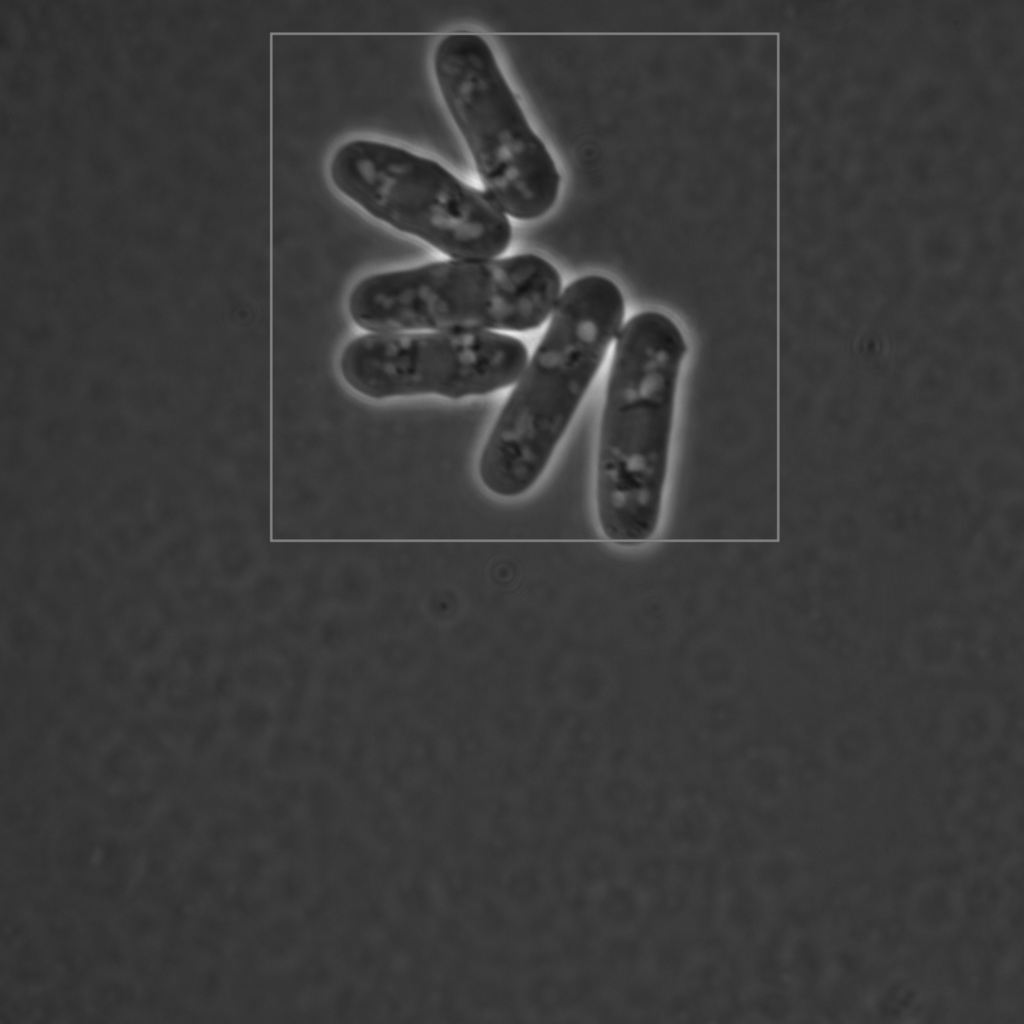

Supplement: Supplementary file 6 — Source data Fig. 2 [file 44318_2025_649_MOESM6_ESM.zip › 121174_Source_Data_Fig_2/Fig_2C/Fig_2C_annotated/Fellas_Fig_2C_BF_hba1_annotated.TIF]

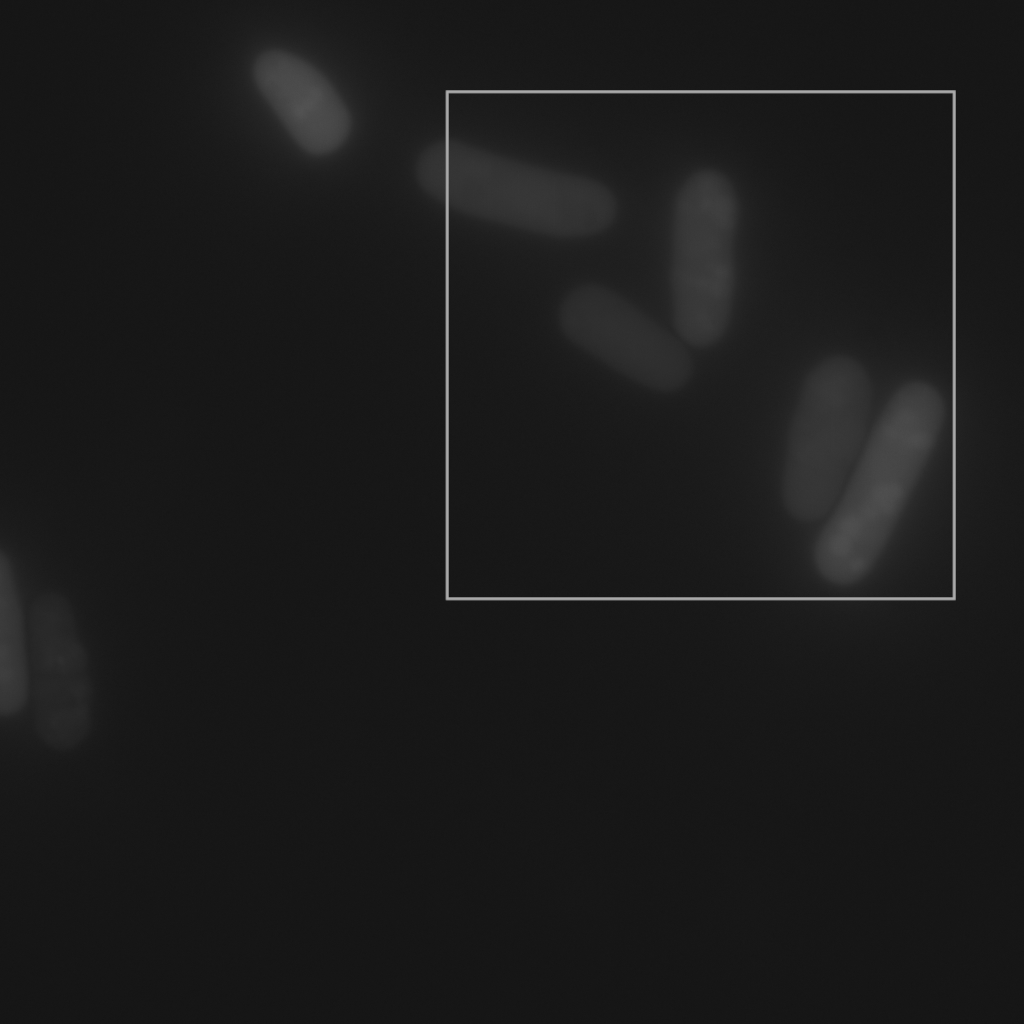

Supplement: Supplementary file 6 — Source data Fig. 2 [file 44318_2025_649_MOESM6_ESM.zip › 121174_Source_Data_Fig_2/Fig_2C/Fig_2C_annotated/Fellas_Fig_2C_DCF_cup1_annotated_bright..TIF]

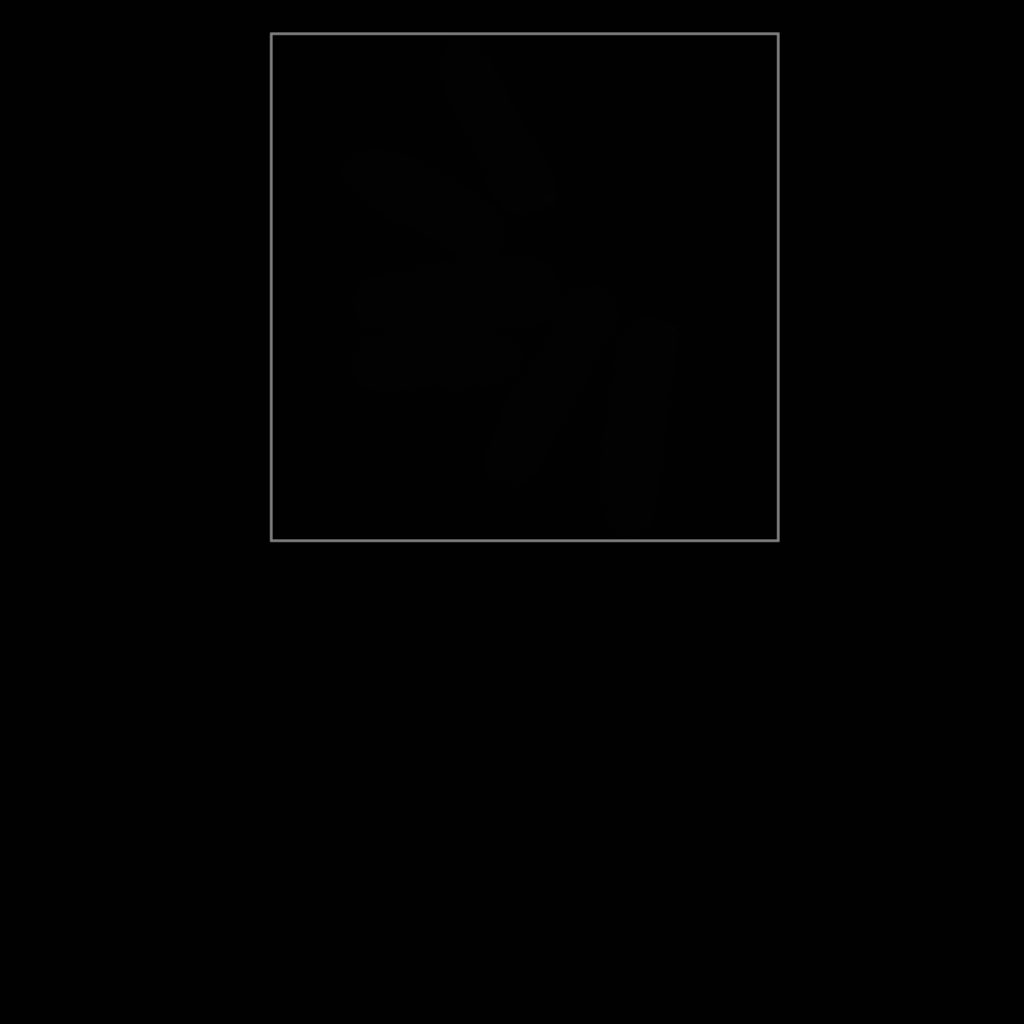

Supplement: Supplementary file 6 — Source data Fig. 2 [file 44318_2025_649_MOESM6_ESM.zip › 121174_Source_Data_Fig_2/Fig_2C/Fig_2C_annotated/Fellas_Fig_2C_DCF_hba1_annotated.TIF]

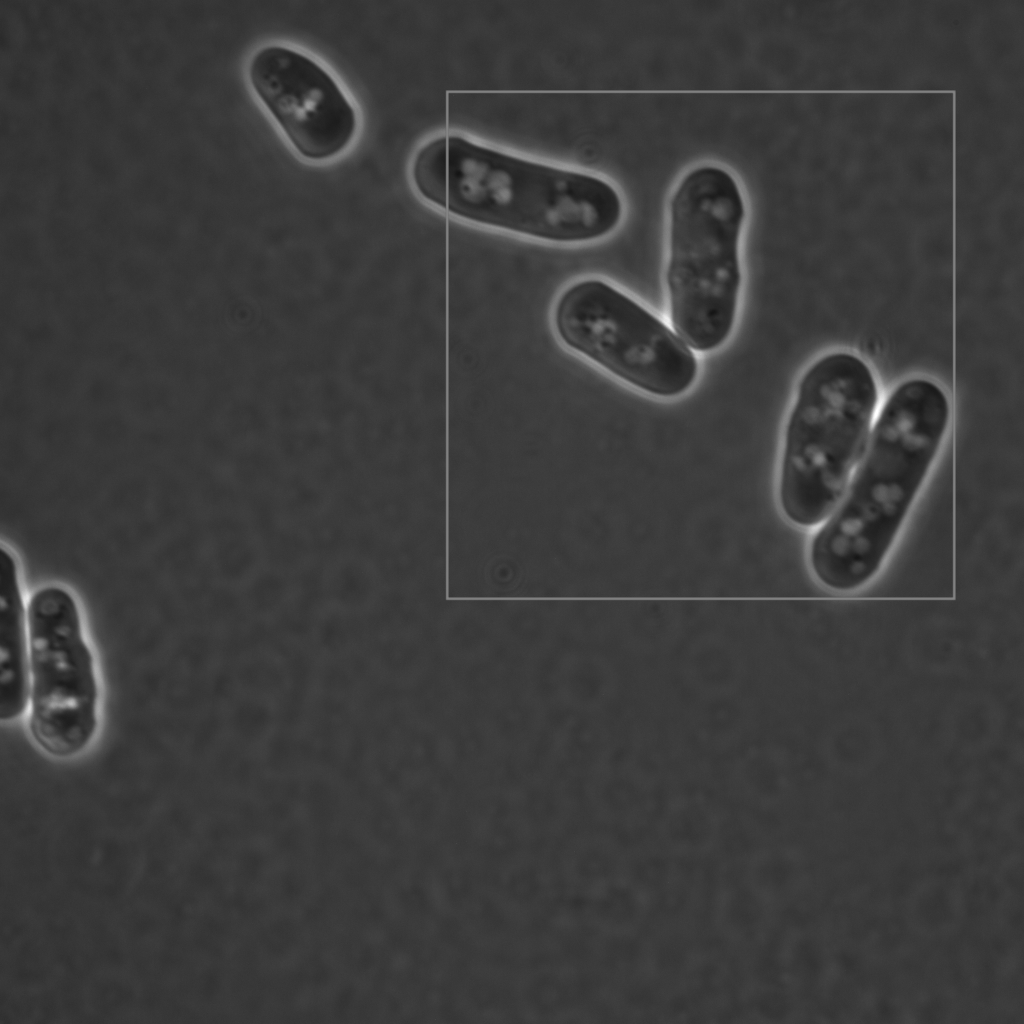

Supplement: Supplementary file 6 — Source data Fig. 2 [file 44318_2025_649_MOESM6_ESM.zip › 121174_Source_Data_Fig_2/Fig_2C/Fig_2C_annotated/Fellas_Fig_2C_BF_cup1_annotated.TIF]

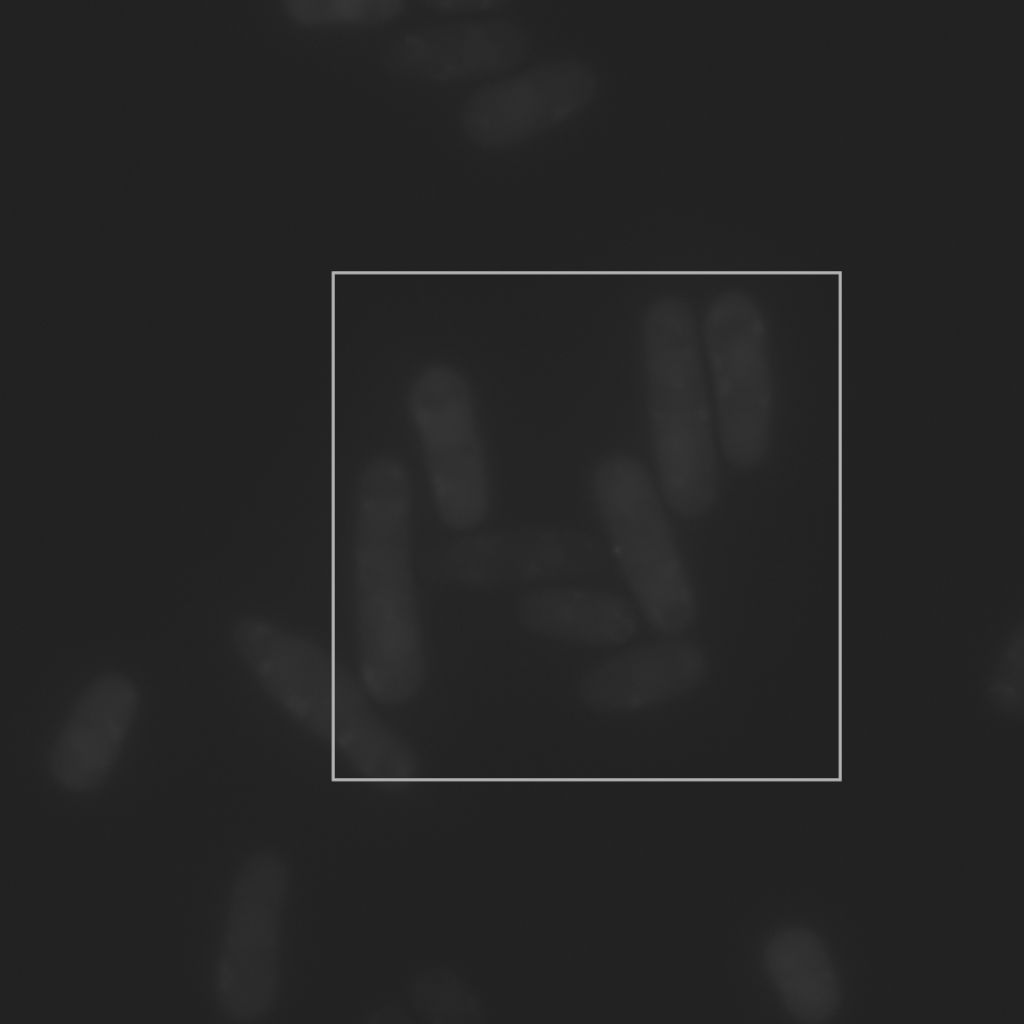

Supplement: Supplementary file 6 — Source data Fig. 2 [file 44318_2025_649_MOESM6_ESM.zip › 121174_Source_Data_Fig_2/Fig_2C/Fig_2C_annotated/Fellas_Fig_2C_DCF_wt_annotated_bright.TIF]

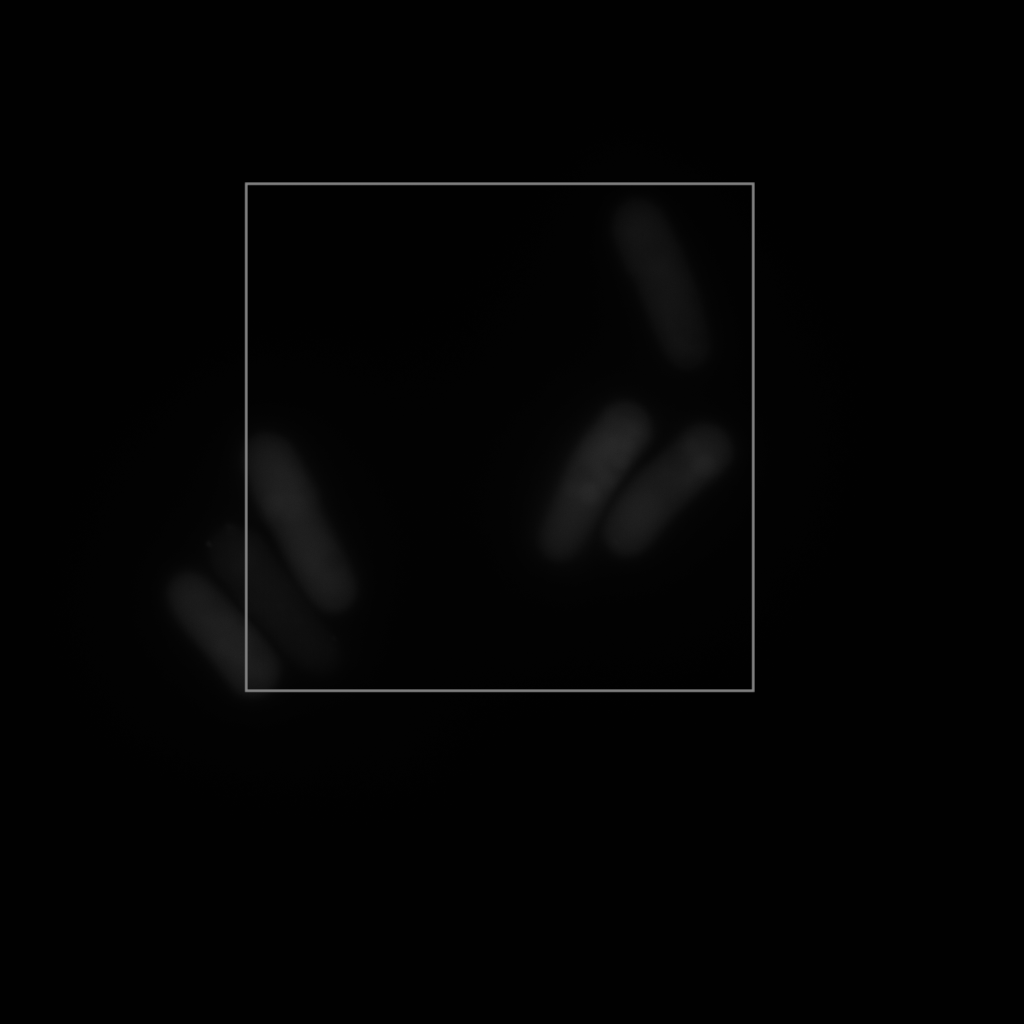

Supplement: Supplementary file 6 — Source data Fig. 2 [file 44318_2025_649_MOESM6_ESM.zip › 121174_Source_Data_Fig_2/Fig_2C/Fig_2C_annotated/Fellas_Fig_2C_DCF_ppr4_annotated.TIF]

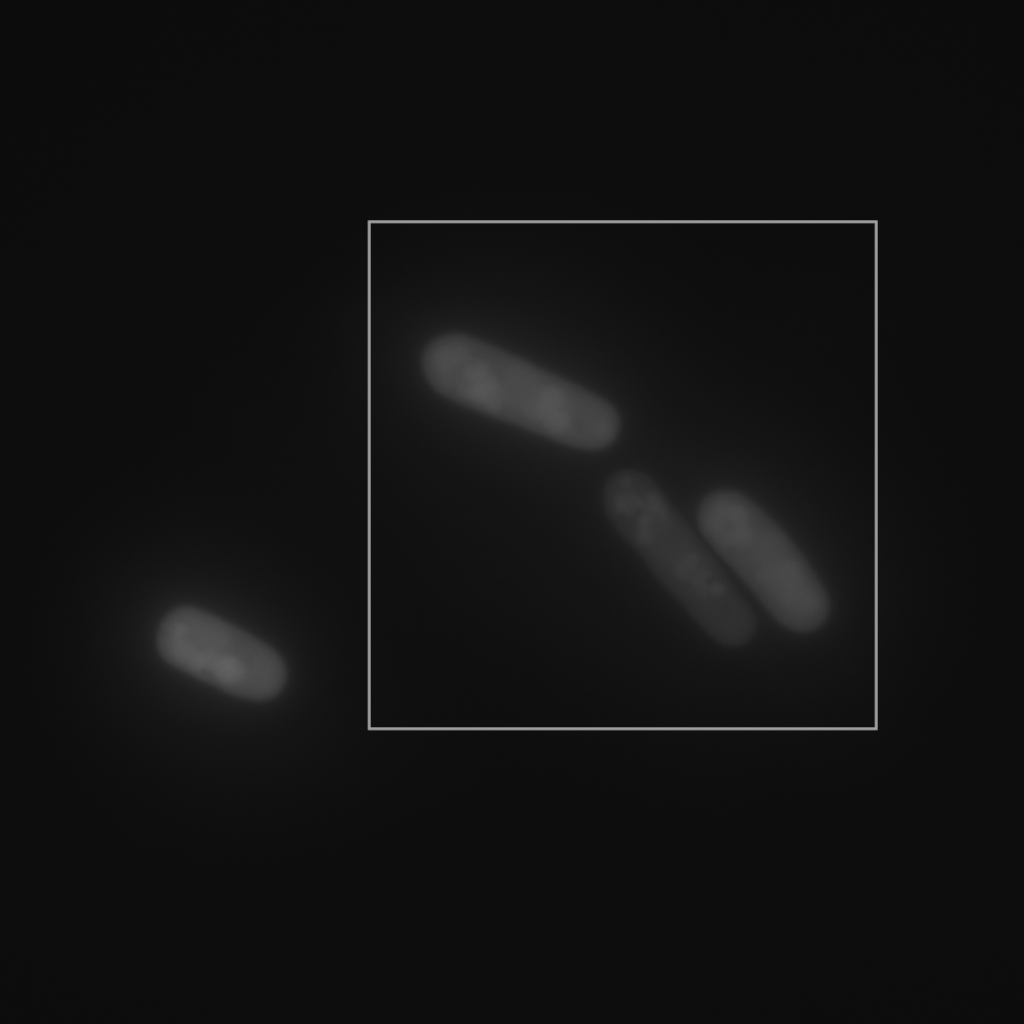

Supplement: Supplementary file 6 — Source data Fig. 2 [file 44318_2025_649_MOESM6_ESM.zip › 121174_Source_Data_Fig_2/Fig_2C/Fig_2C_annotated/Fellas_Fig_2C_DCF_cox4_annotated_bright..TIF]

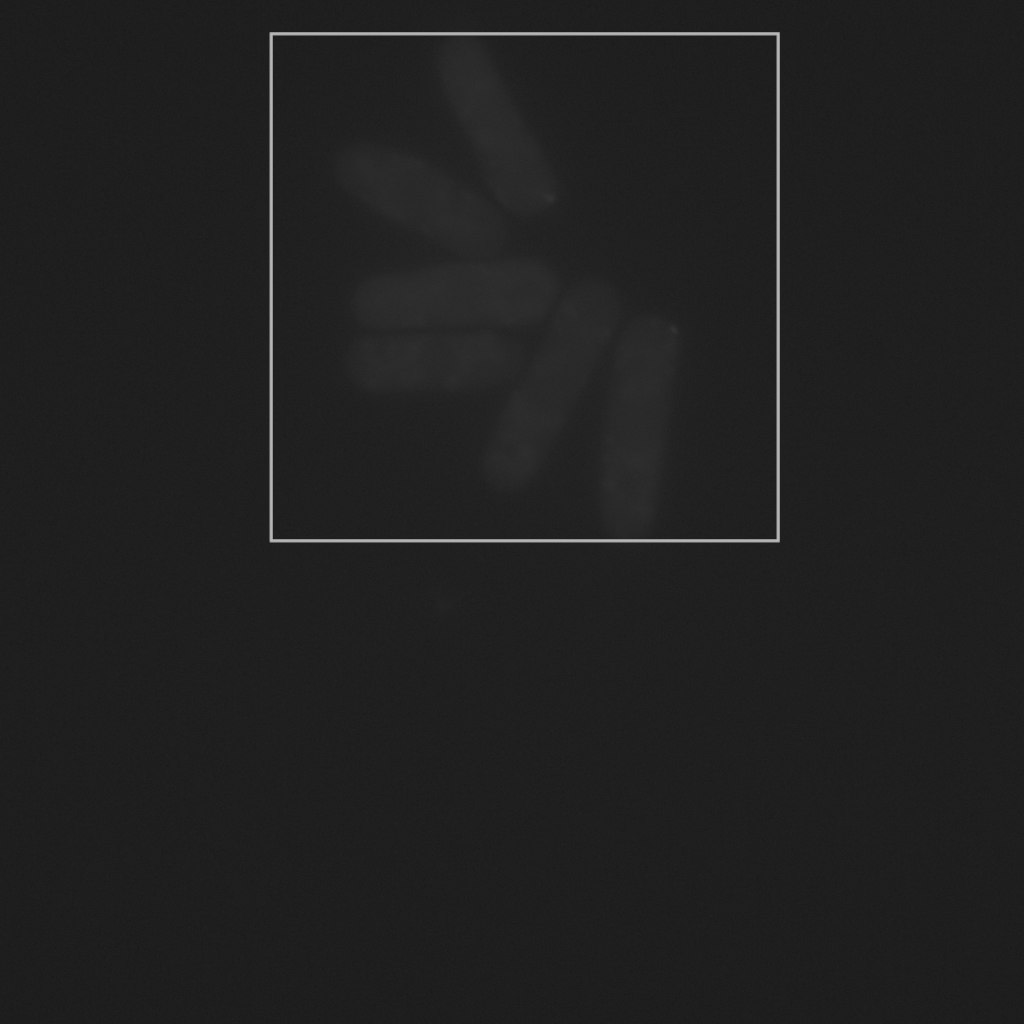

Supplement: Supplementary file 6 — Source data Fig. 2 [file 44318_2025_649_MOESM6_ESM.zip › 121174_Source_Data_Fig_2/Fig_2C/Fig_2C_annotated/Fellas_Fig_2C_DCF_hba1_annotated_bright..TIF]

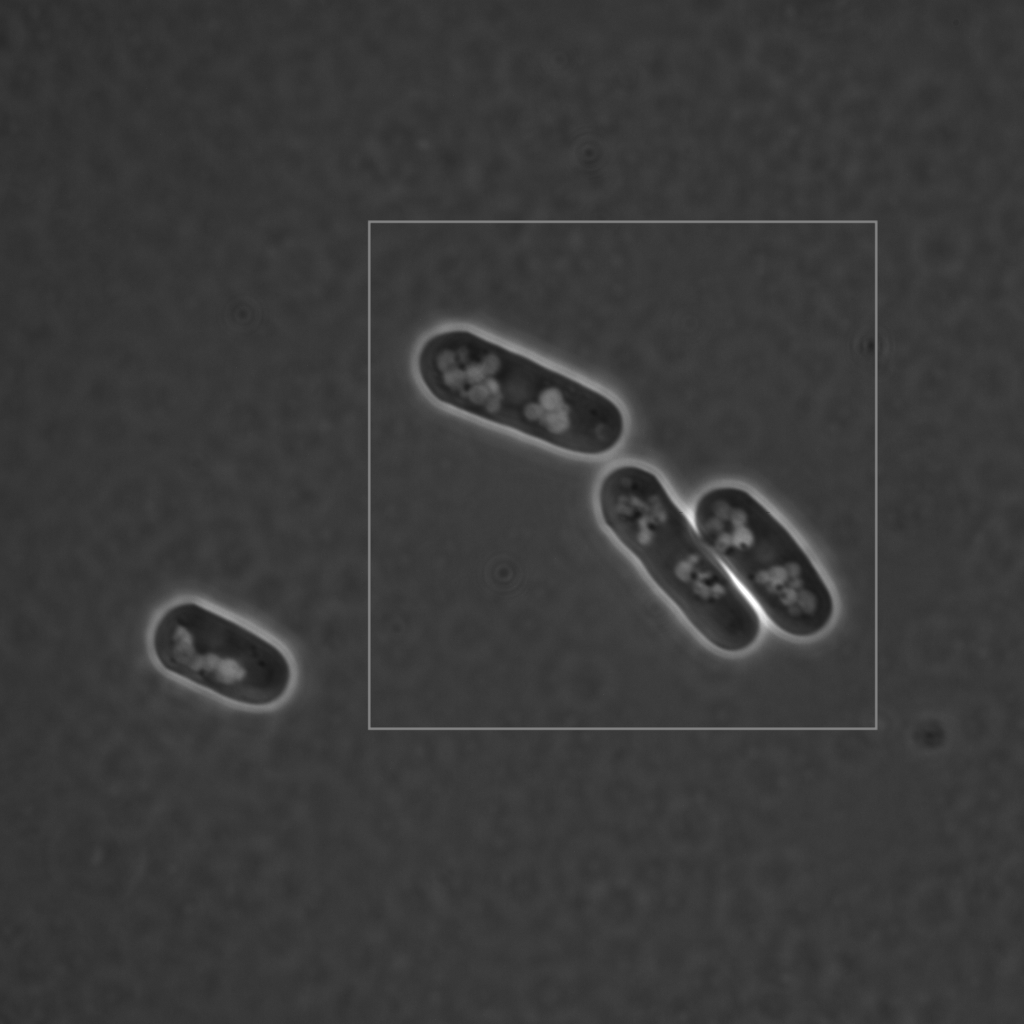

Supplement: Supplementary file 6 — Source data Fig. 2 [file 44318_2025_649_MOESM6_ESM.zip › 121174_Source_Data_Fig_2/Fig_2C/Fig_2C_annotated/Fellas_Fig_2C_BF_cox4_annotated.TIF]

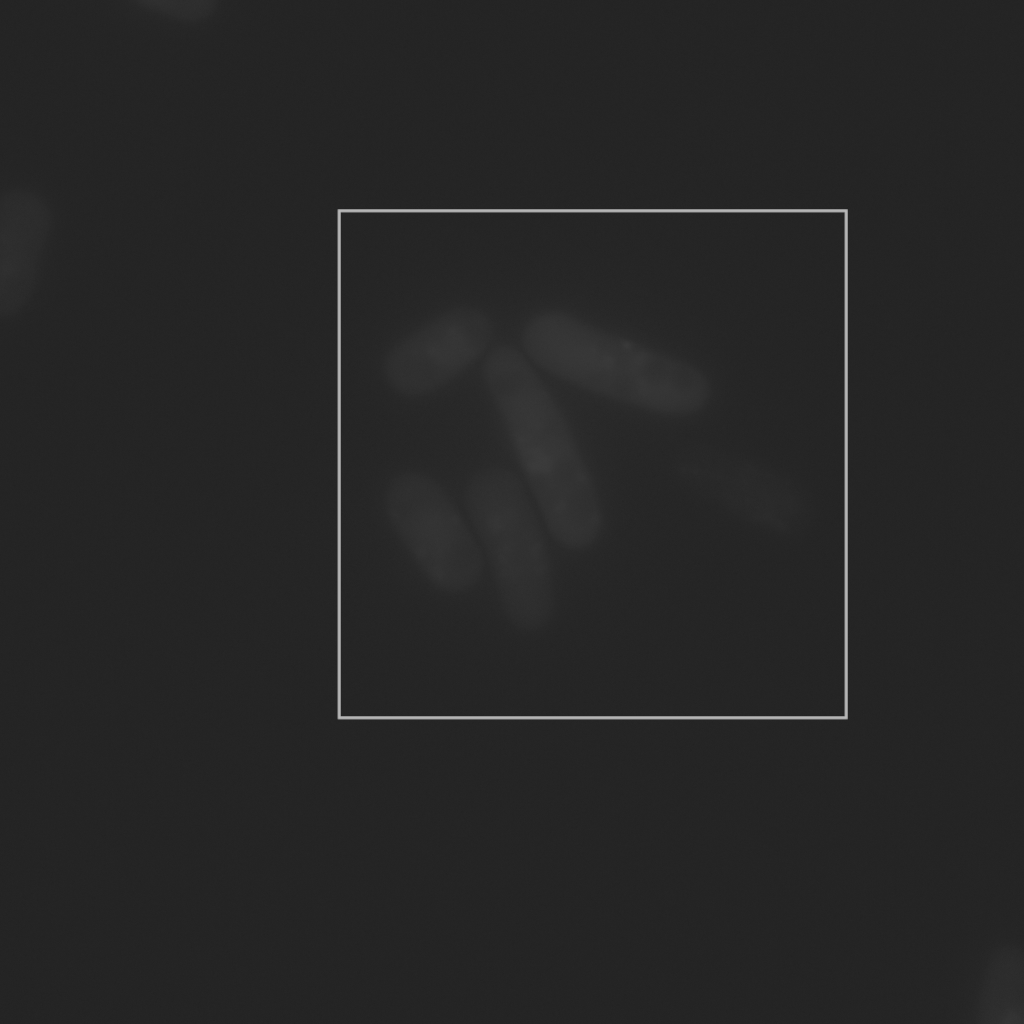

Supplement: Supplementary file 6 — Source data Fig. 2 [file 44318_2025_649_MOESM6_ESM.zip › 121174_Source_Data_Fig_2/Fig_2C/Fig_2C_annotated/Fellas_Fig_2C_DCF_sdh7_annotated_bright..TIF]

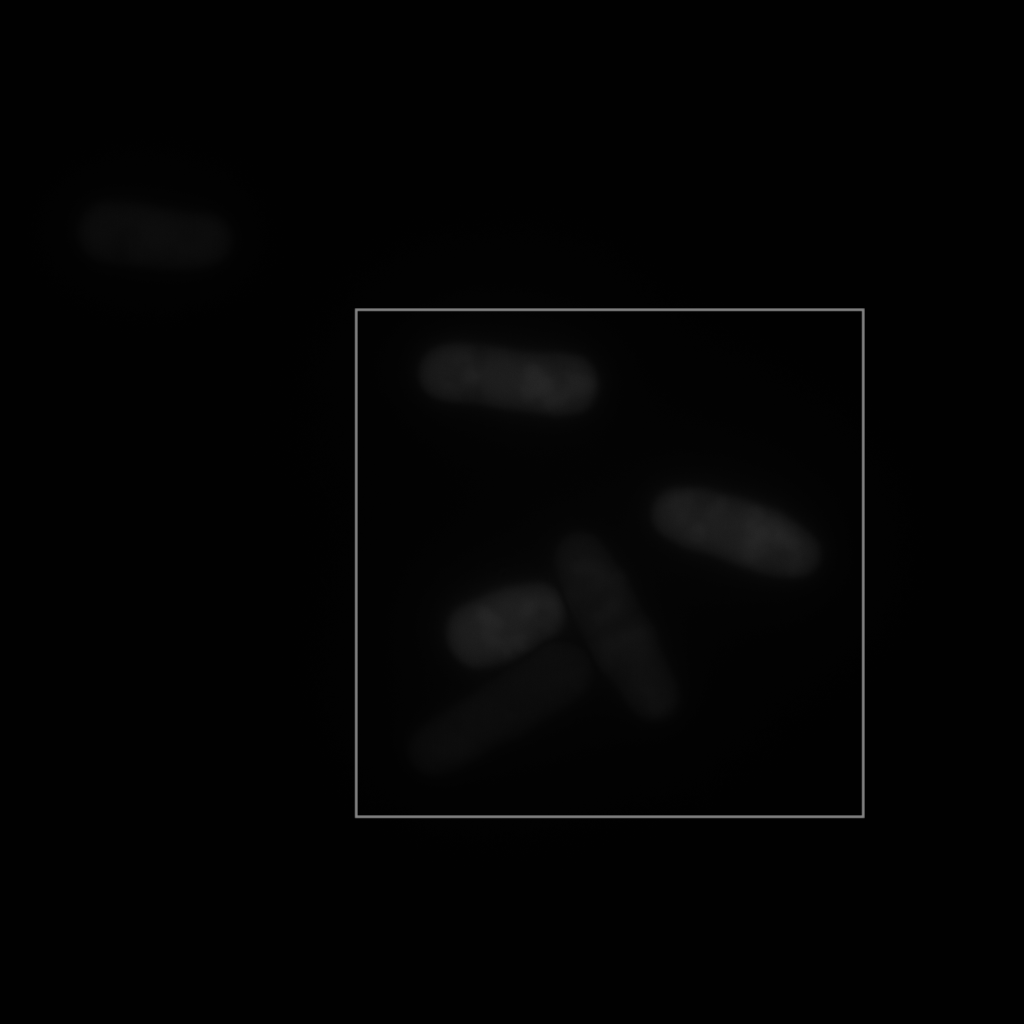

Supplement: Supplementary file 6 — Source data Fig. 2 [file 44318_2025_649_MOESM6_ESM.zip › 121174_Source_Data_Fig_2/Fig_2C/Fig_2C_annotated/Fellas_Fig_2C_DCF_qcr7_annotated.TIF]

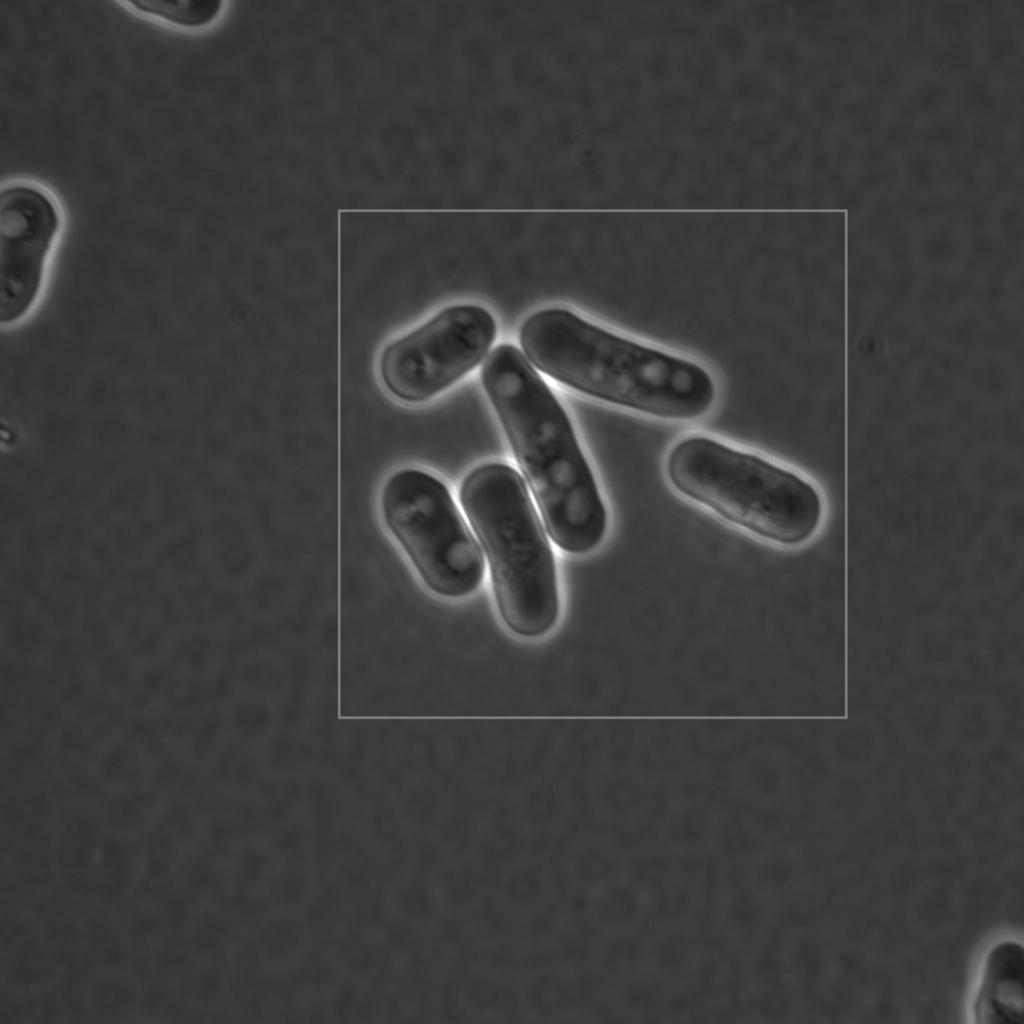

Supplement: Supplementary file 6 — Source data Fig. 2 [file 44318_2025_649_MOESM6_ESM.zip › 121174_Source_Data_Fig_2/Fig_2C/Fig_2C_annotated/Fellas_Fig_2C_BF_sdh7_annotated.TIF]

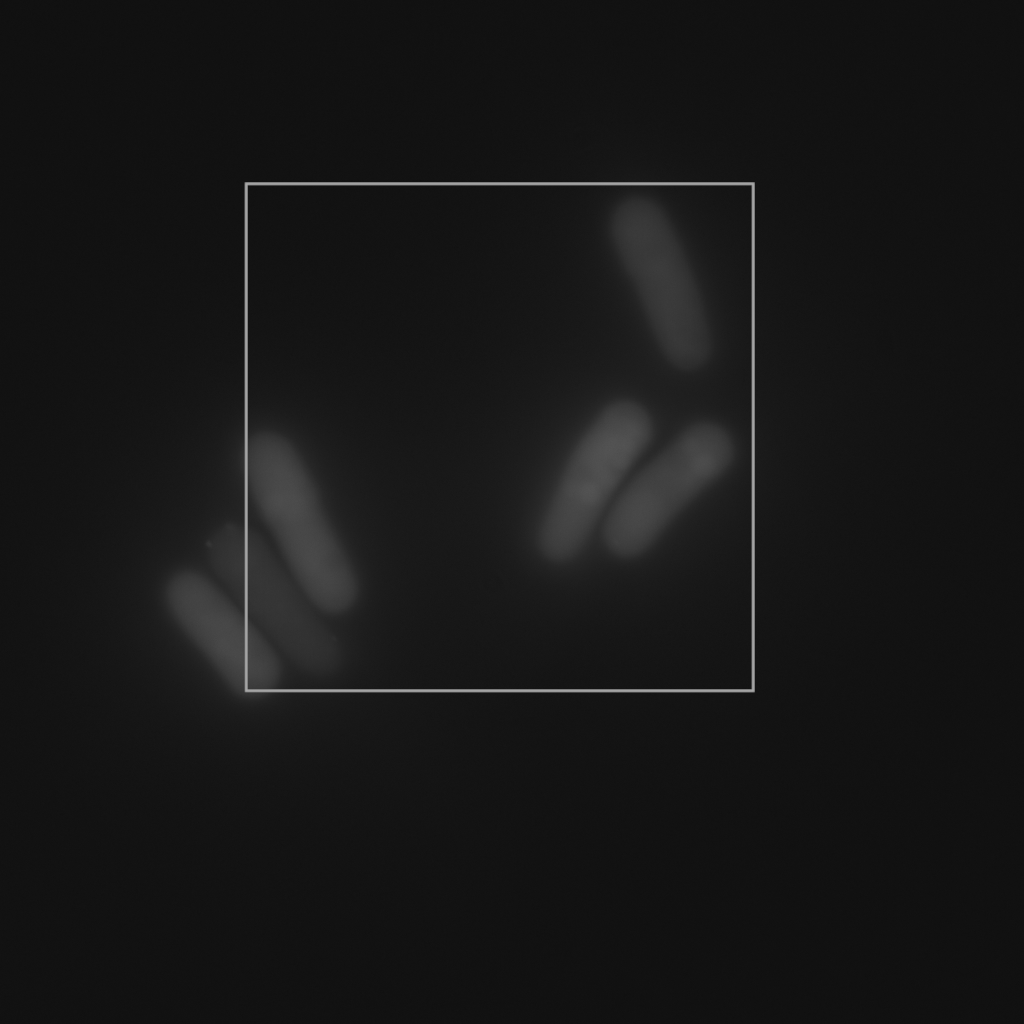

Supplement: Supplementary file 6 — Source data Fig. 2 [file 44318_2025_649_MOESM6_ESM.zip › 121174_Source_Data_Fig_2/Fig_2C/Fig_2C_annotated/Fellas_Fig_2C_DCF_ppr4_annotated_bright..TIF]

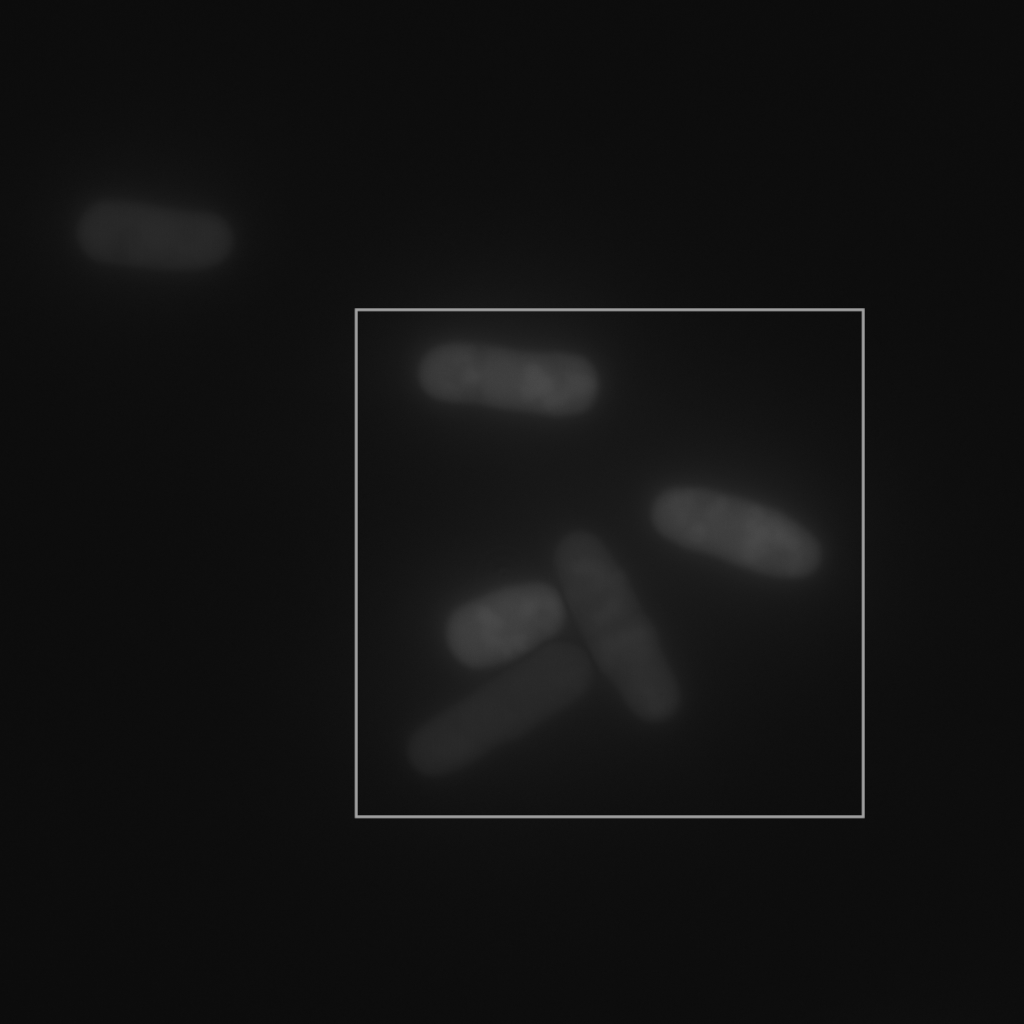

Supplement: Supplementary file 6 — Source data Fig. 2 [file 44318_2025_649_MOESM6_ESM.zip › 121174_Source_Data_Fig_2/Fig_2C/Fig_2C_annotated/Fellas_Fig_2C_DCF_qcr7_annotated_bright..TIF]

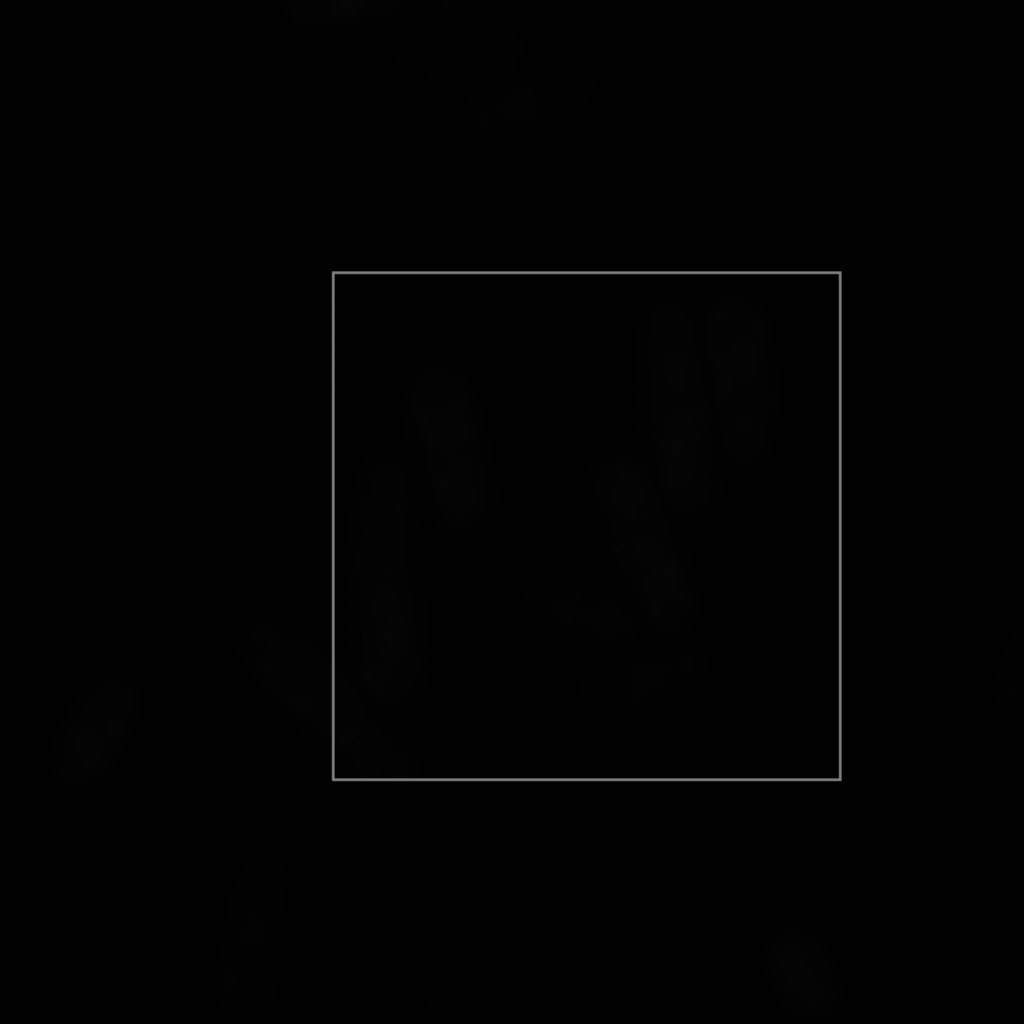

Supplement: Supplementary file 6 — Source data Fig. 2 [file 44318_2025_649_MOESM6_ESM.zip › 121174_Source_Data_Fig_2/Fig_2C/Fig_2C_annotated/Fellas_Fig_2C_DCF_wt_annotated.TIF]

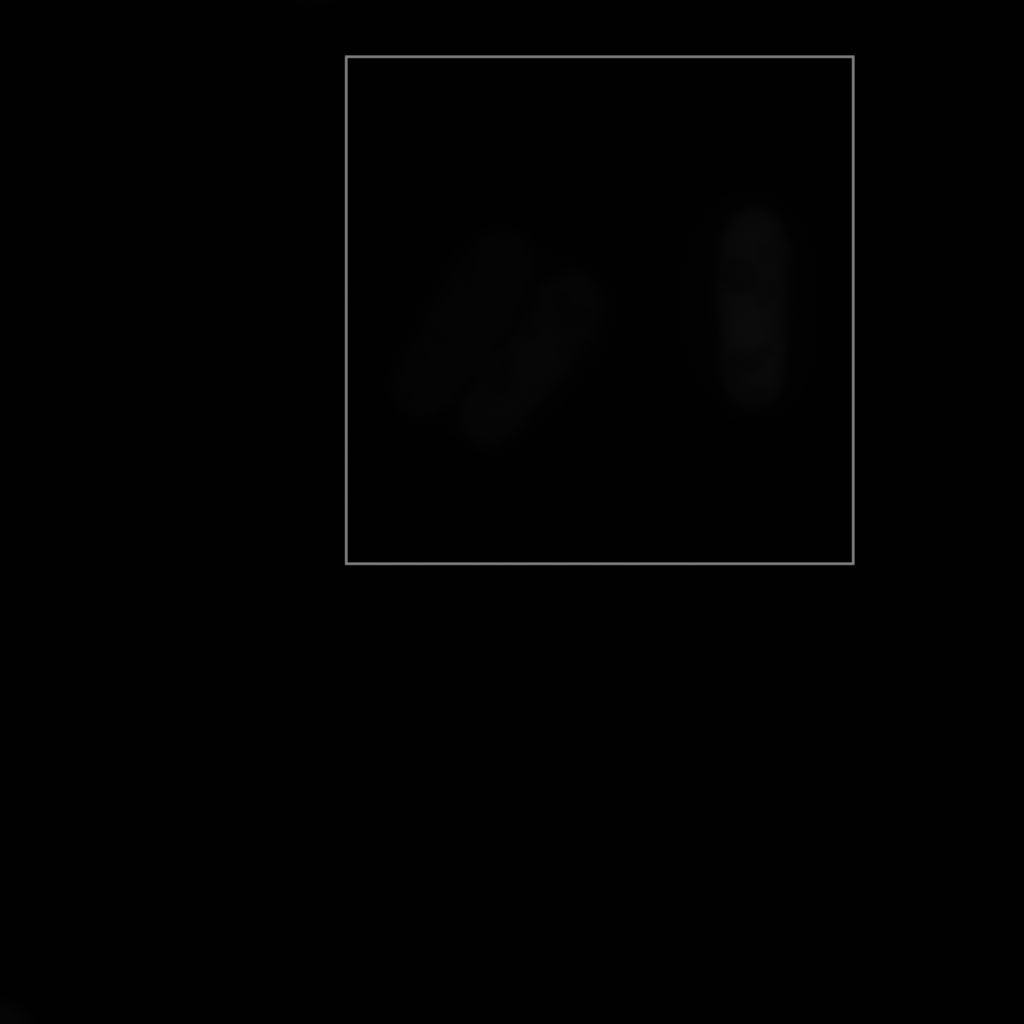

Supplement: Supplementary file 6 — Source data Fig. 2 [file 44318_2025_649_MOESM6_ESM.zip › 121174_Source_Data_Fig_2/Fig_2C/Fig_2C_annotated/Fellas_Fig_2C_DCF_ndi1_annotated.TIF]

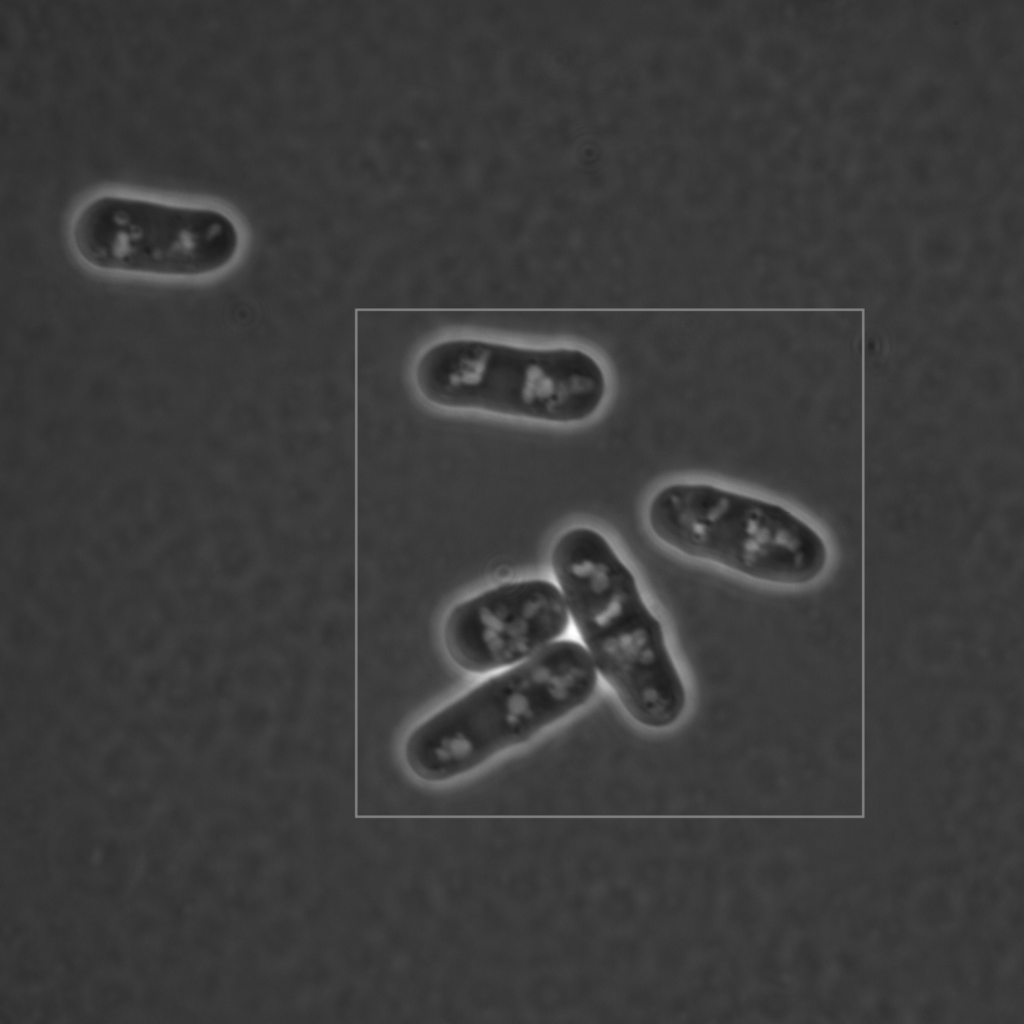

Supplement: Supplementary file 6 — Source data Fig. 2 [file 44318_2025_649_MOESM6_ESM.zip › 121174_Source_Data_Fig_2/Fig_2C/Fig_2C_annotated/Fellas_Fig_2C_BF_qcr7_annotated.TIF]

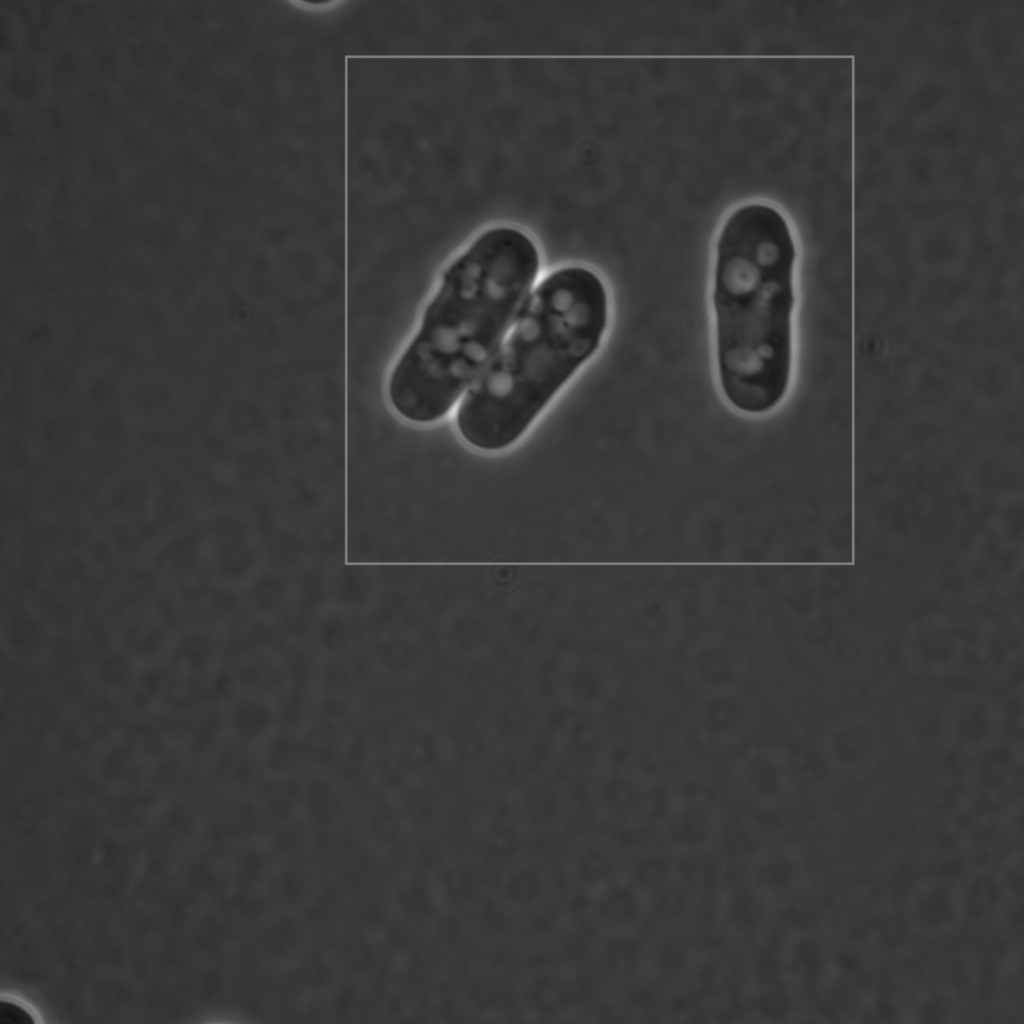

Supplement: Supplementary file 6 — Source data Fig. 2 [file 44318_2025_649_MOESM6_ESM.zip › 121174_Source_Data_Fig_2/Fig_2C/Fig_2C_annotated/Fellas_Fig_2C_BF_ndi1_annotated.TIF]

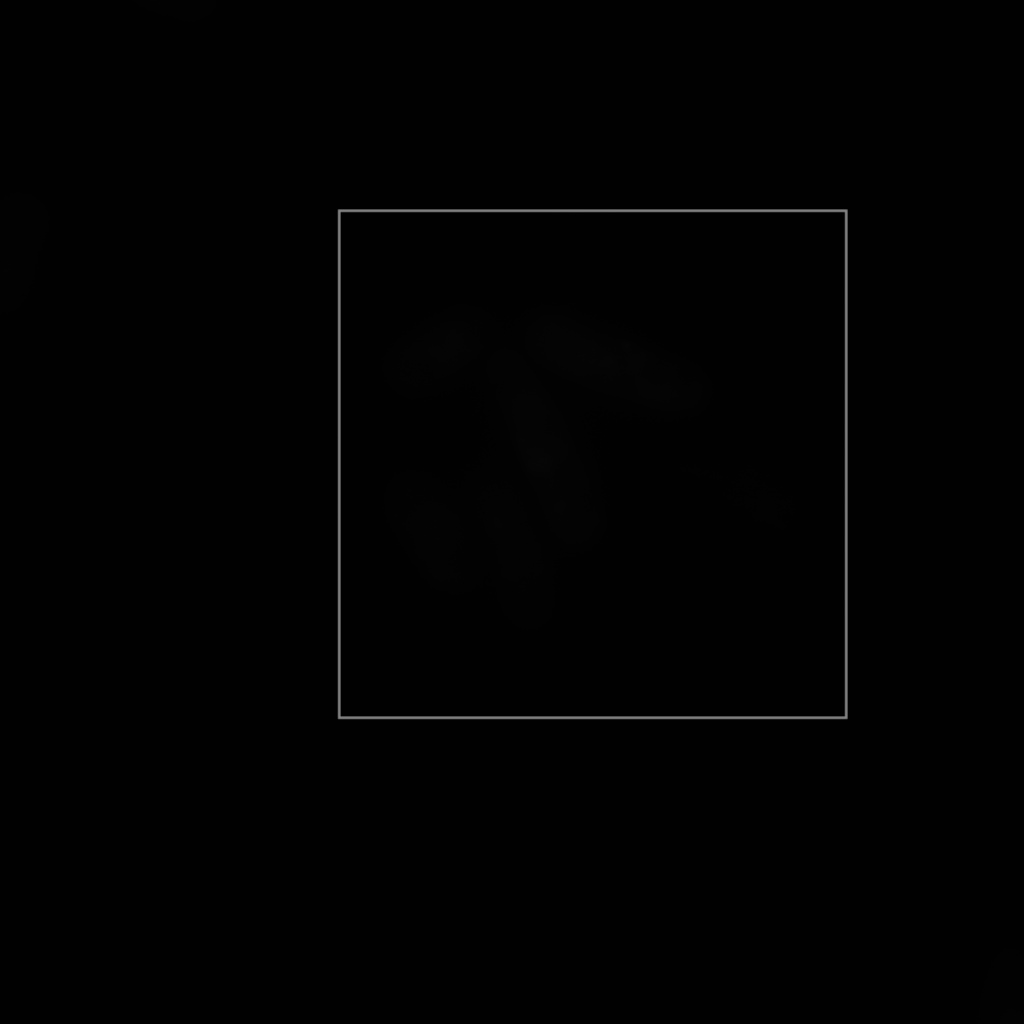

Supplement: Supplementary file 6 — Source data Fig. 2 [file 44318_2025_649_MOESM6_ESM.zip › 121174_Source_Data_Fig_2/Fig_2C/Fig_2C_annotated/Fellas_Fig_2C_DCF_sdh7_annotated.TIF]

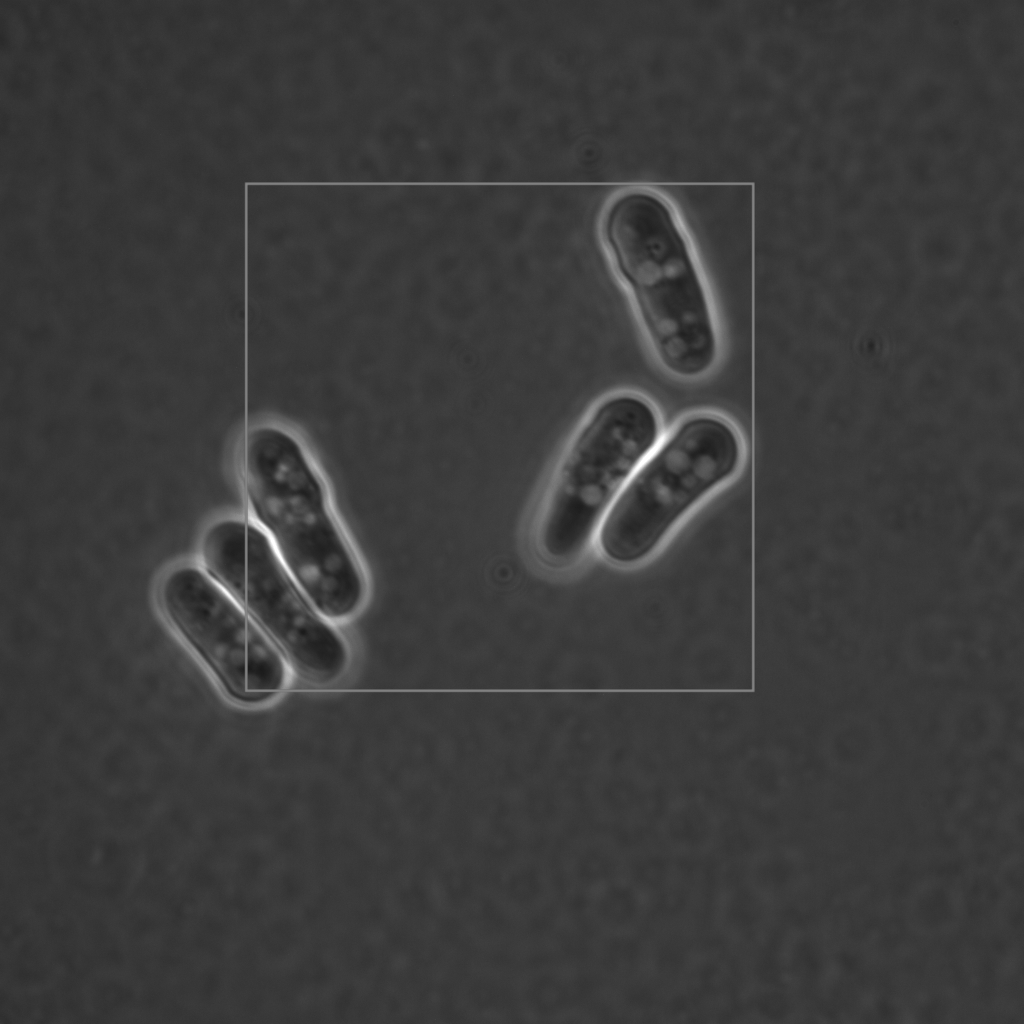

Supplement: Supplementary file 6 — Source data Fig. 2 [file 44318_2025_649_MOESM6_ESM.zip › 121174_Source_Data_Fig_2/Fig_2C/Fig_2C_annotated/Fellas_Fig_2C_BF_ppr4_annotated.TIF]

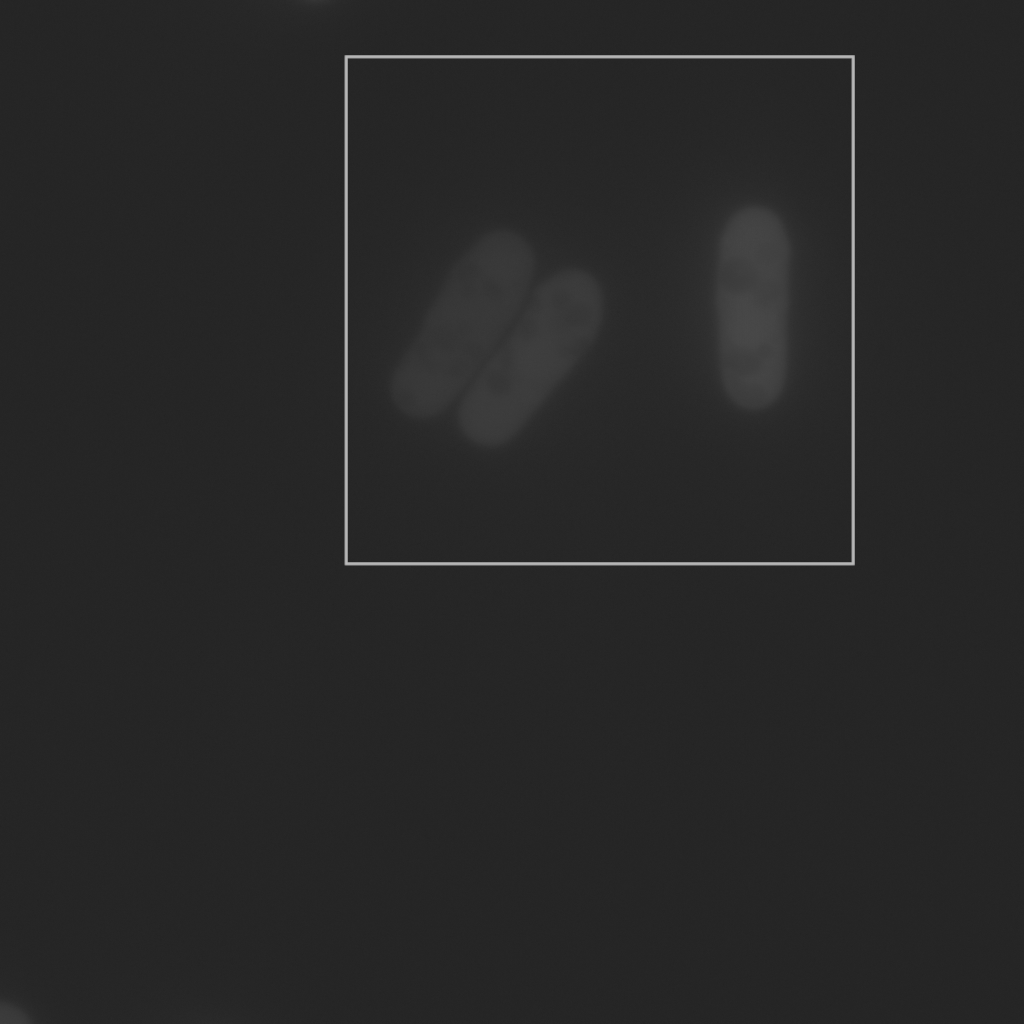

Supplement: Supplementary file 6 — Source data Fig. 2 [file 44318_2025_649_MOESM6_ESM.zip › 121174_Source_Data_Fig_2/Fig_2C/Fig_2C_annotated/Fellas_Fig_2C_DCF_ndi1_annotated_bright..TIF]

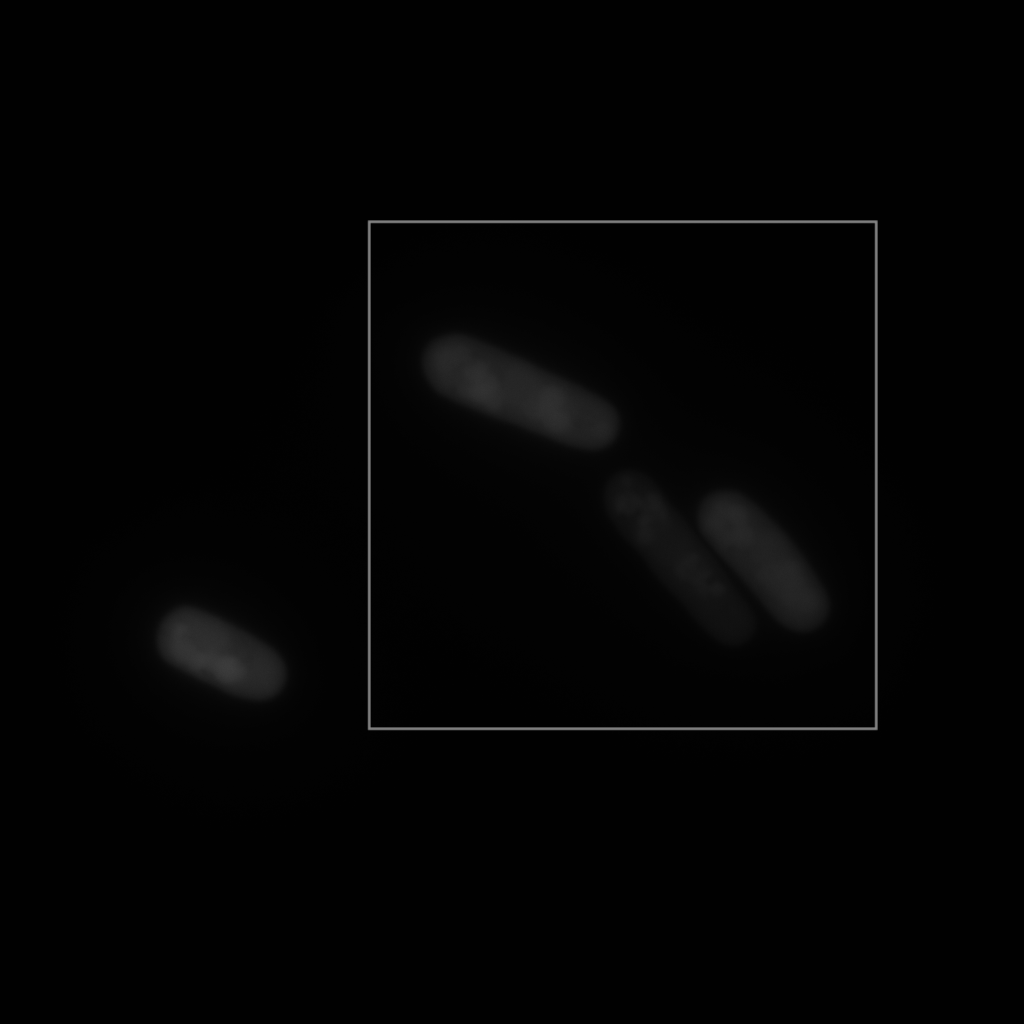

Supplement: Supplementary file 6 — Source data Fig. 2 [file 44318_2025_649_MOESM6_ESM.zip › 121174_Source_Data_Fig_2/Fig_2C/Fig_2C_annotated/Fellas_Fig_2C_DCF_cox4_annotated.TIF]

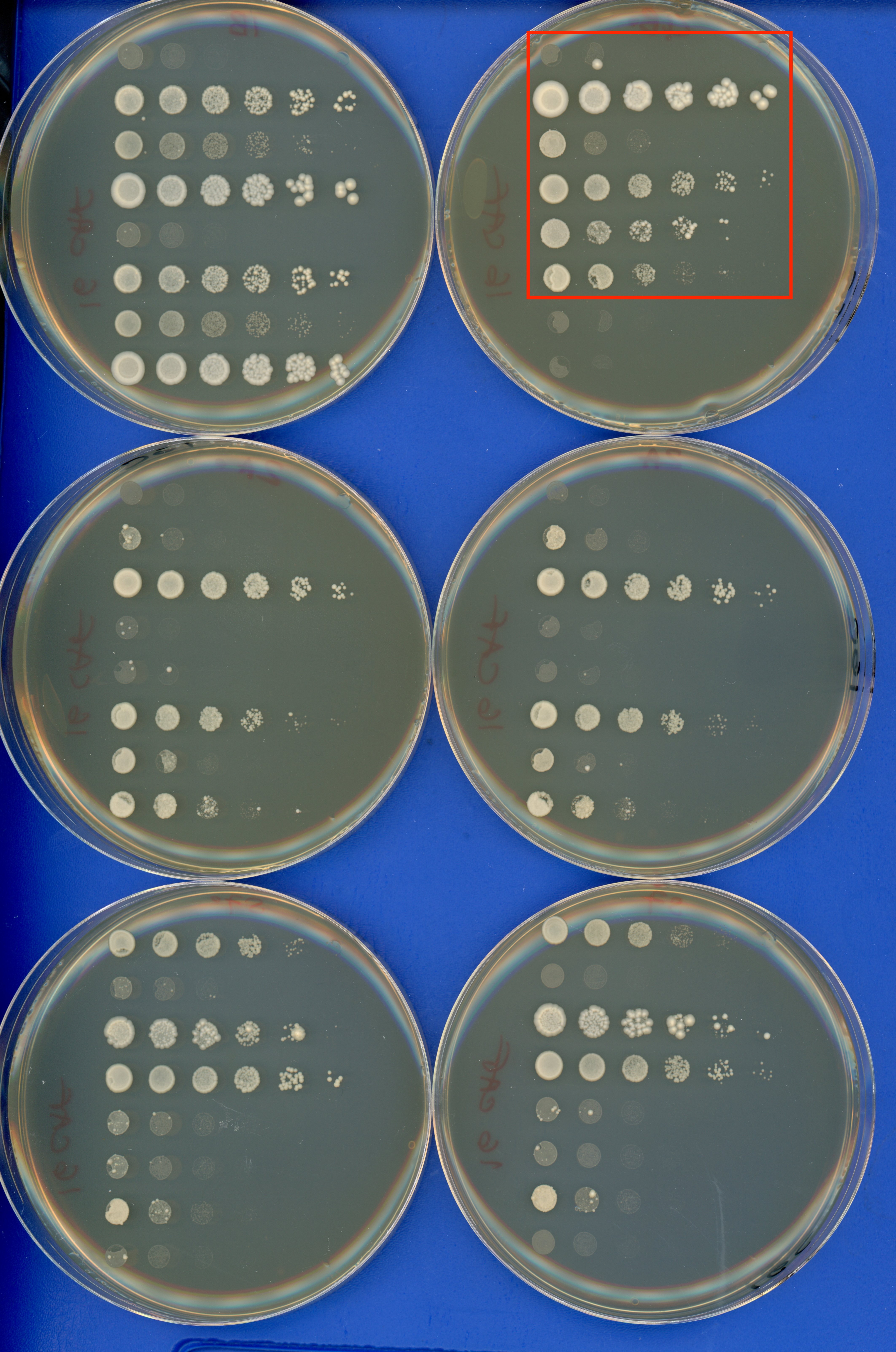

Supplement: Supplementary file 8 — Source data Fig. 4 [file 44318_2025_649_MOESM8_ESM.zip › 121174_Source_Data_Fig_4/Fig_4B/Fellas_Fig_4B_16CAF_annotated.jpg]

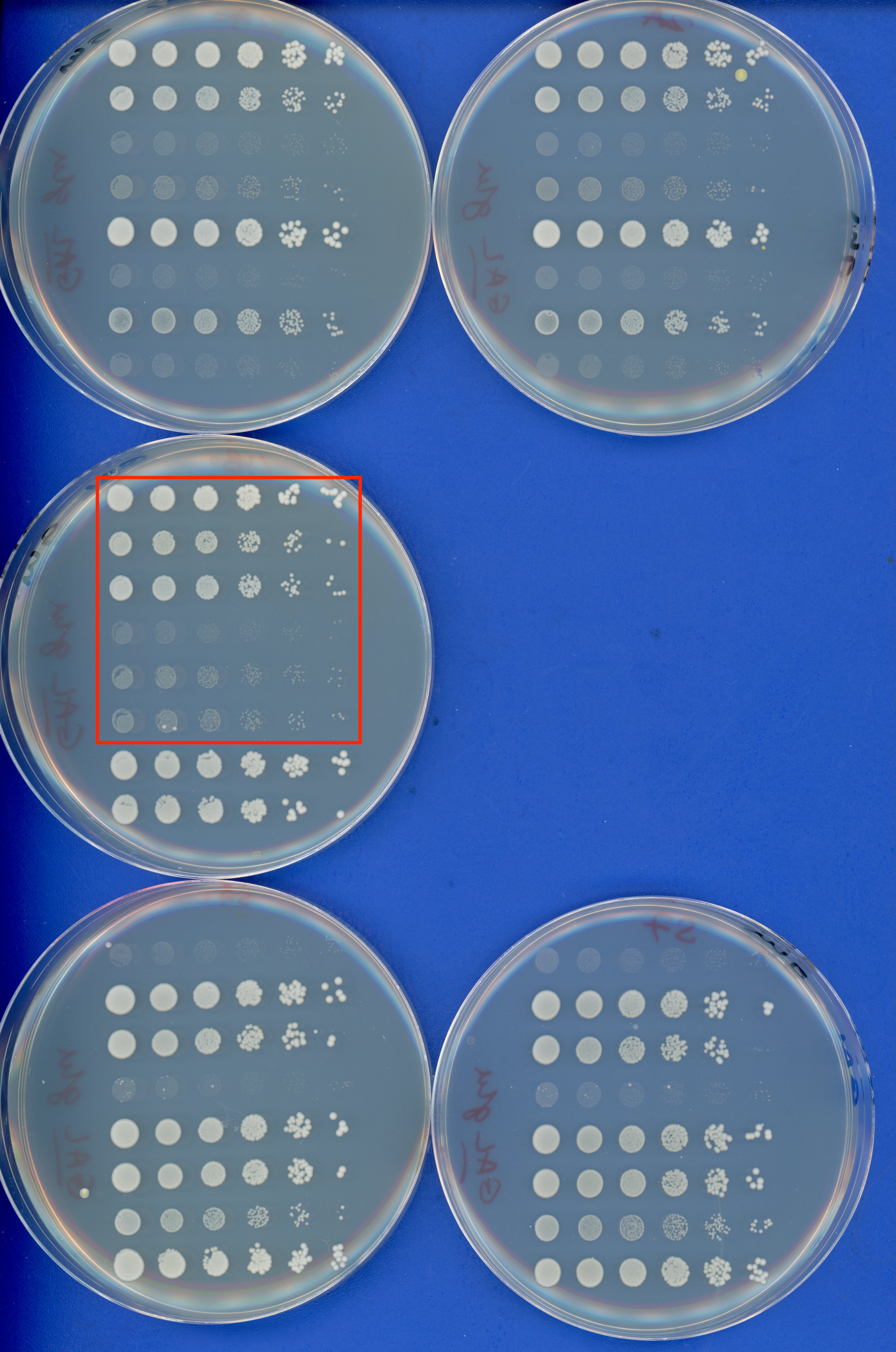

Supplement: Supplementary file 8 — Source data Fig. 4 [file 44318_2025_649_MOESM8_ESM.zip › 121174_Source_Data_Fig_4/Fig_4B/Fellas_Fig_4B_GALglu_annotated.jpg]

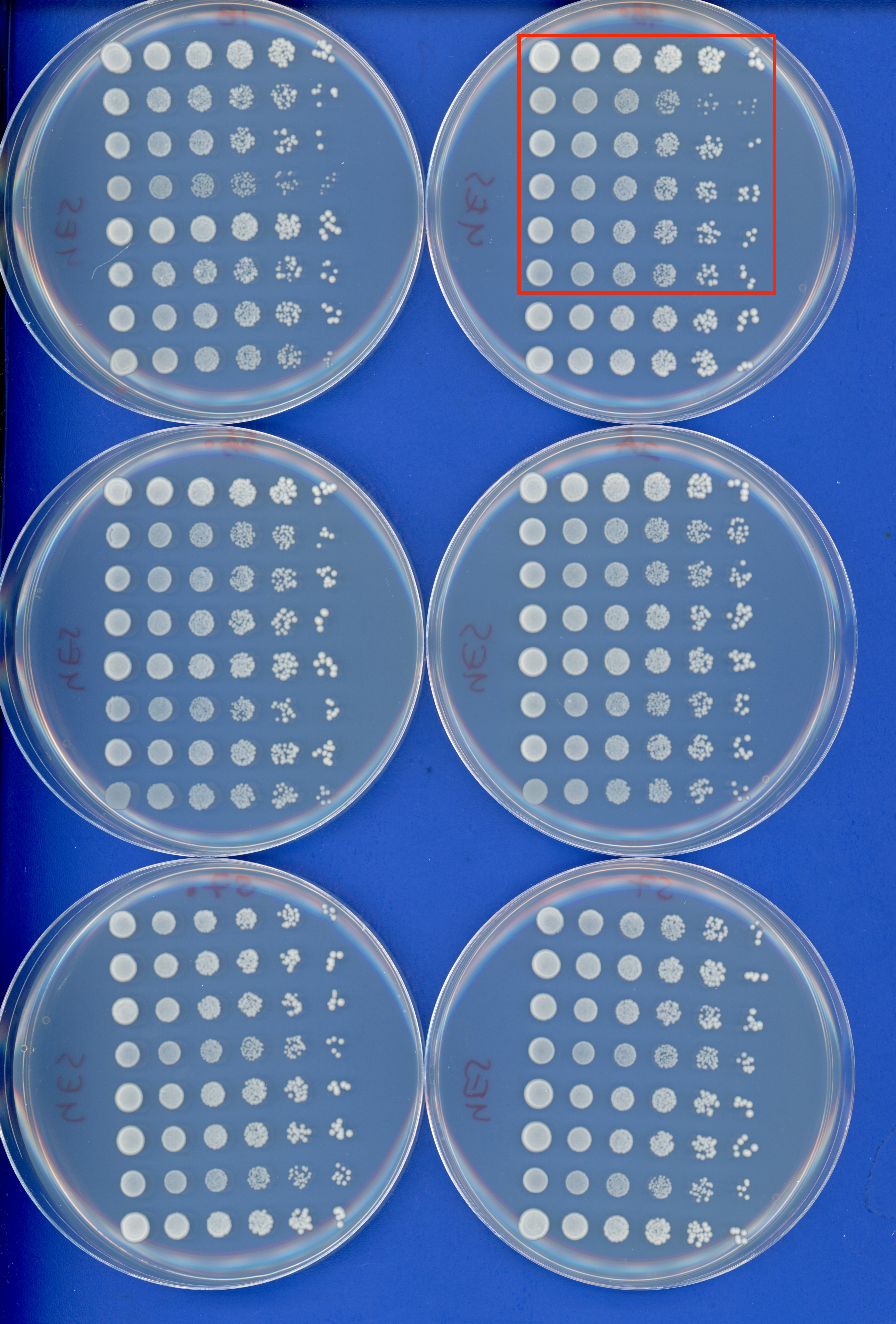

Supplement: Supplementary file 8 — Source data Fig. 4 [file 44318_2025_649_MOESM8_ESM.zip › 121174_Source_Data_Fig_4/Fig_4B/Fellas_Fig_4B_YES_annotated.jpg]

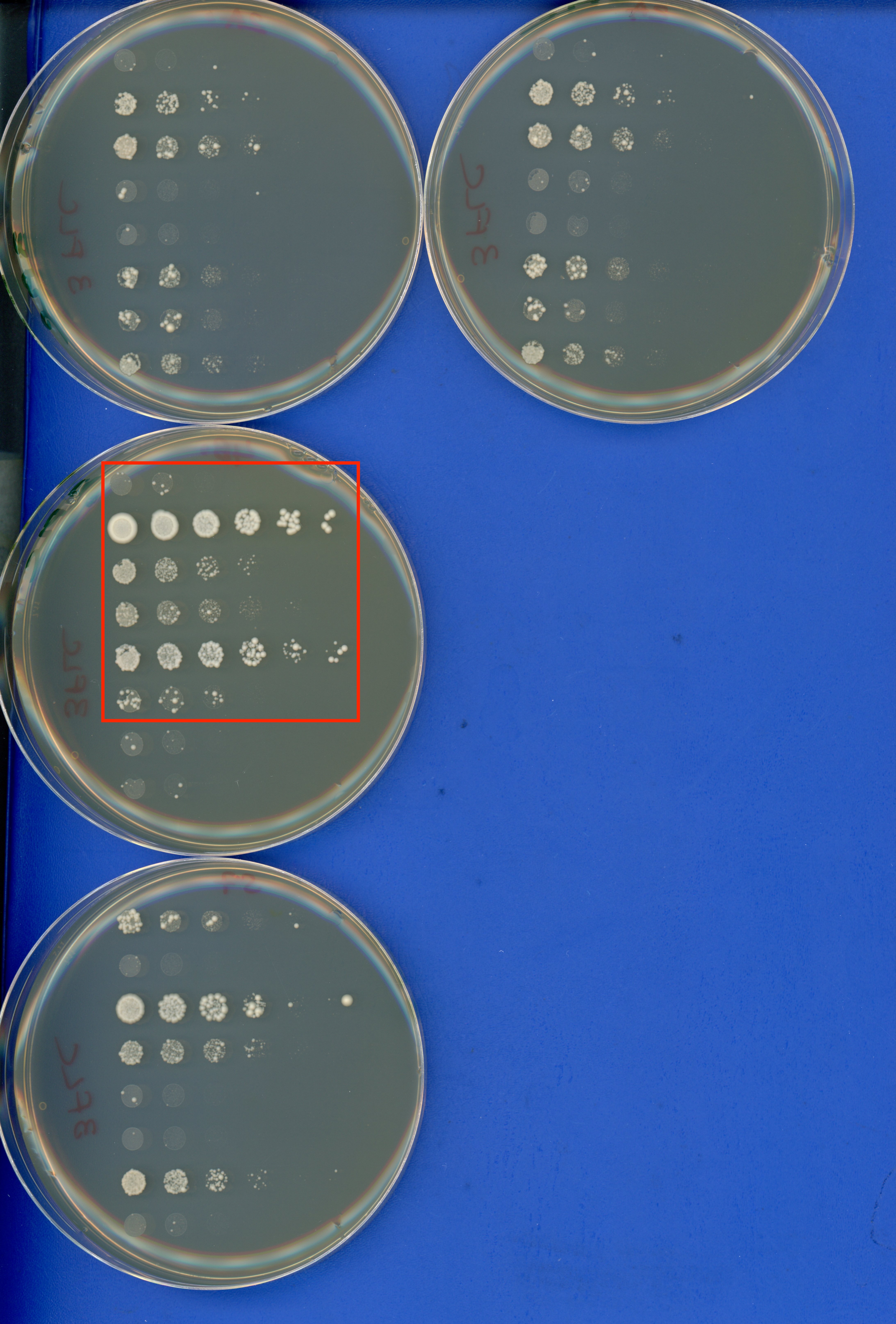

Supplement: Supplementary file 8 — Source data Fig. 4 [file 44318_2025_649_MOESM8_ESM.zip › 121174_Source_Data_Fig_4/Fig_4B/Fellas_Fig_4B_FLC_annotated.jpg]

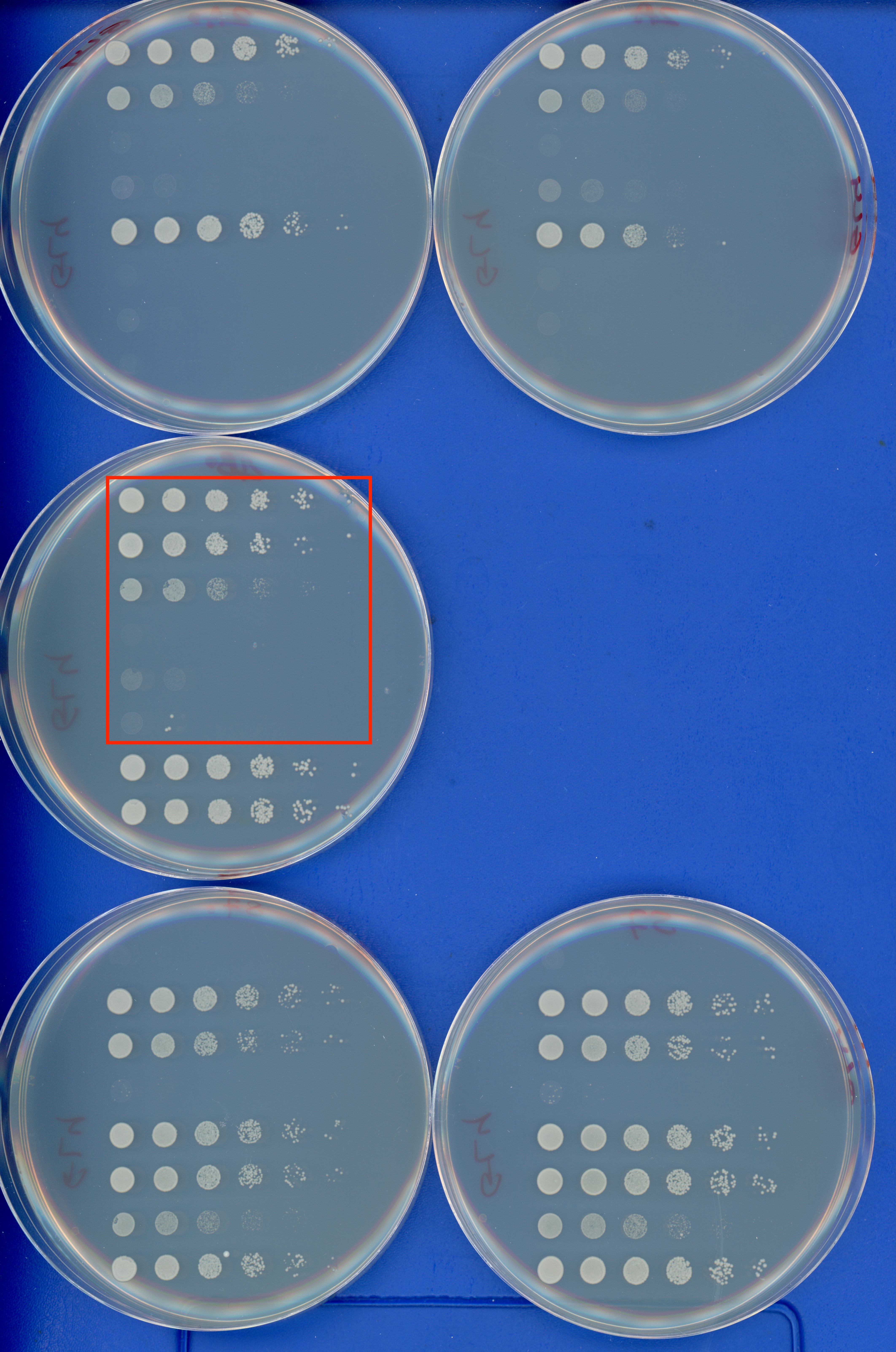

Supplement: Supplementary file 8 — Source data Fig. 4 [file 44318_2025_649_MOESM8_ESM.zip › 121174_Source_Data_Fig_4/Fig_4B/Fellas_Fig_4B_GLY_annotated.jpg]

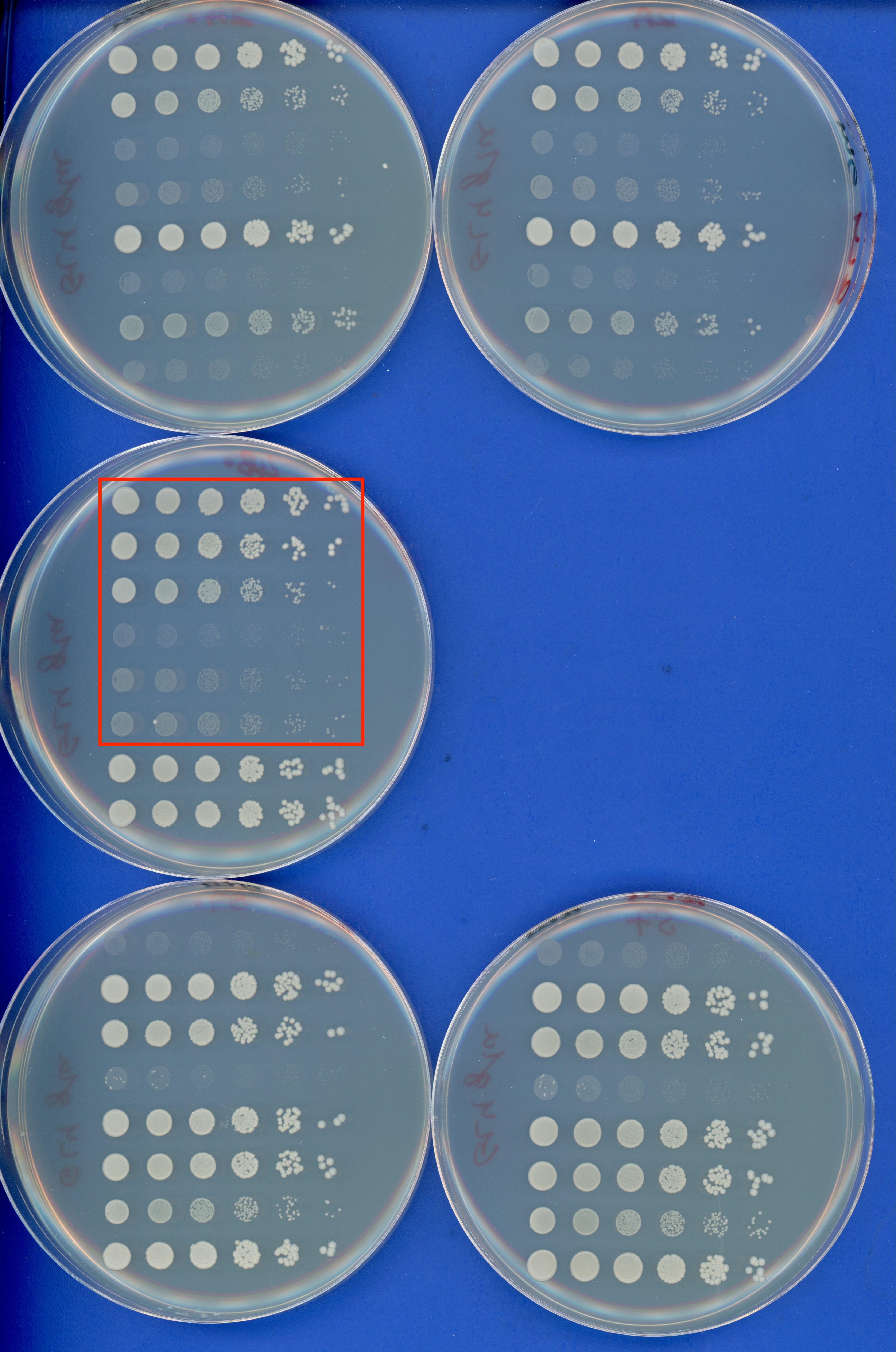

Supplement: Supplementary file 8 — Source data Fig. 4 [file 44318_2025_649_MOESM8_ESM.zip › 121174_Source_Data_Fig_4/Fig_4B/Fellas_Fig_4B_GLYglu_annotated.jpg]

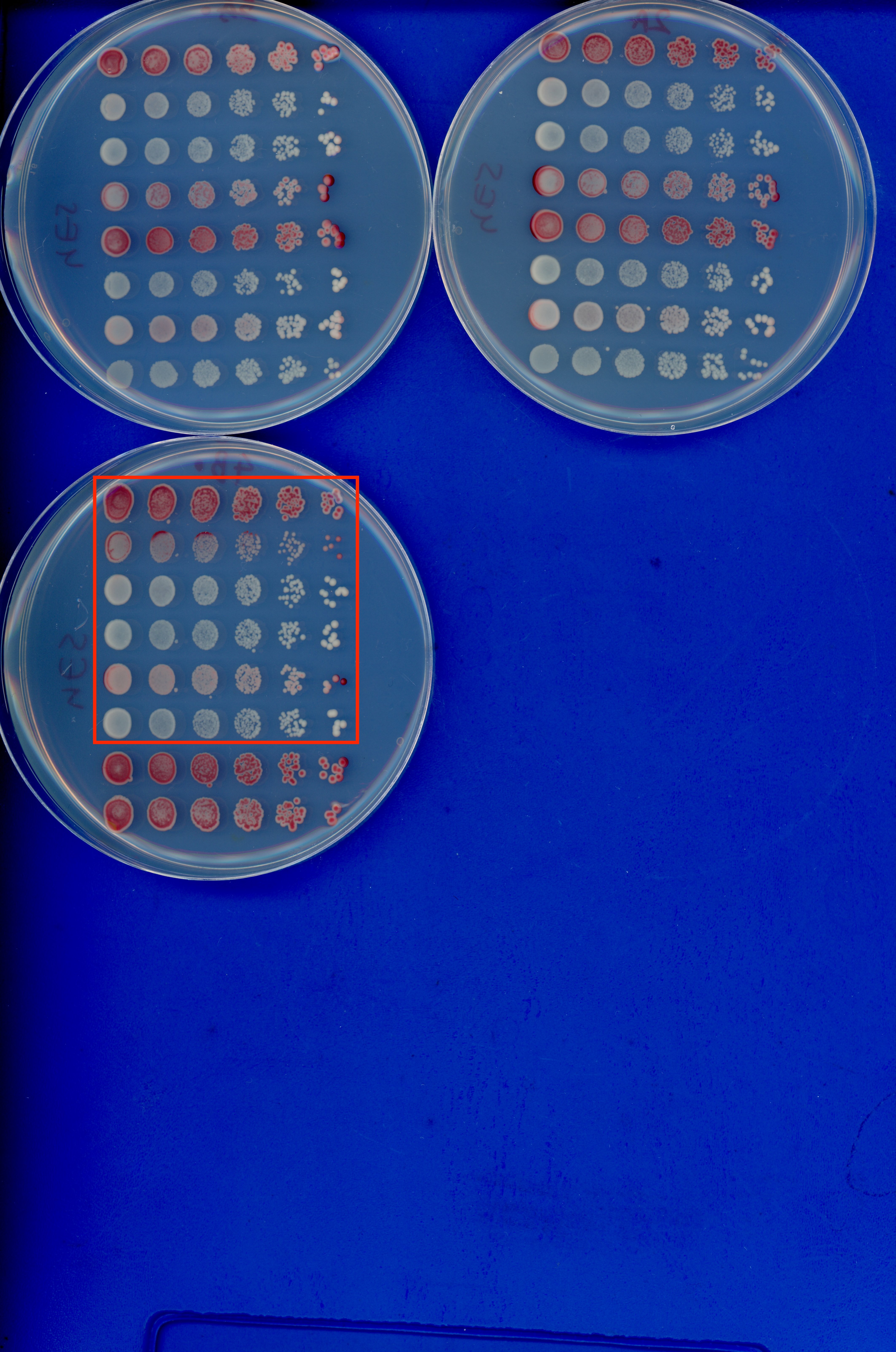

Supplement: Supplementary file 8 — Source data Fig. 4 [file 44318_2025_649_MOESM8_ESM.zip › 121174_Source_Data_Fig_4/Fig_4B/Fellas_Fig_4B_TTC_annotated.jpg]

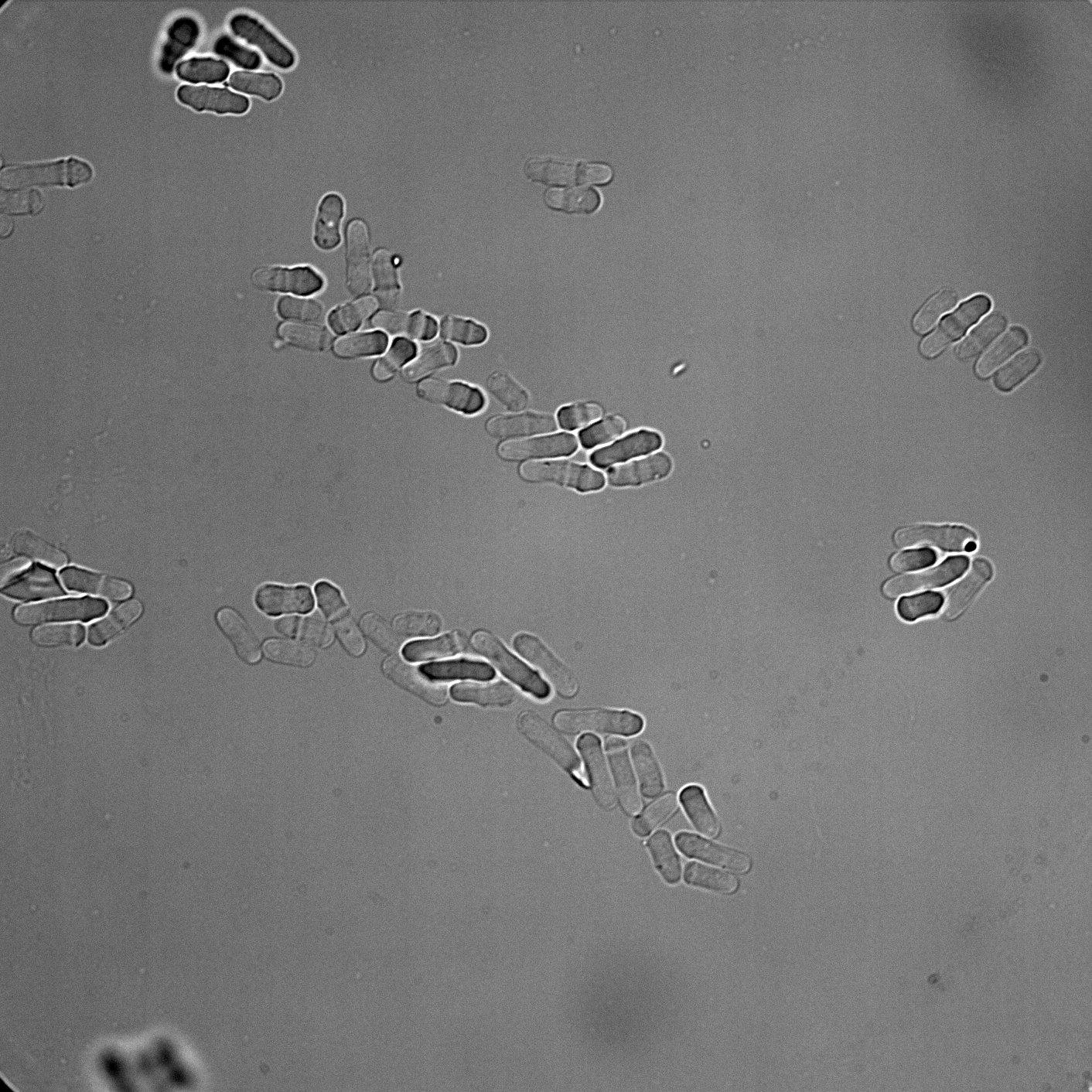

Supplement: Supplementary file 9 — Source data Fig. 5 [file 44318_2025_649_MOESM9_ESM.zip › 121174_Source_Data_Fig_5/Fig_5B/B5676_004-1_brightfield.tif]

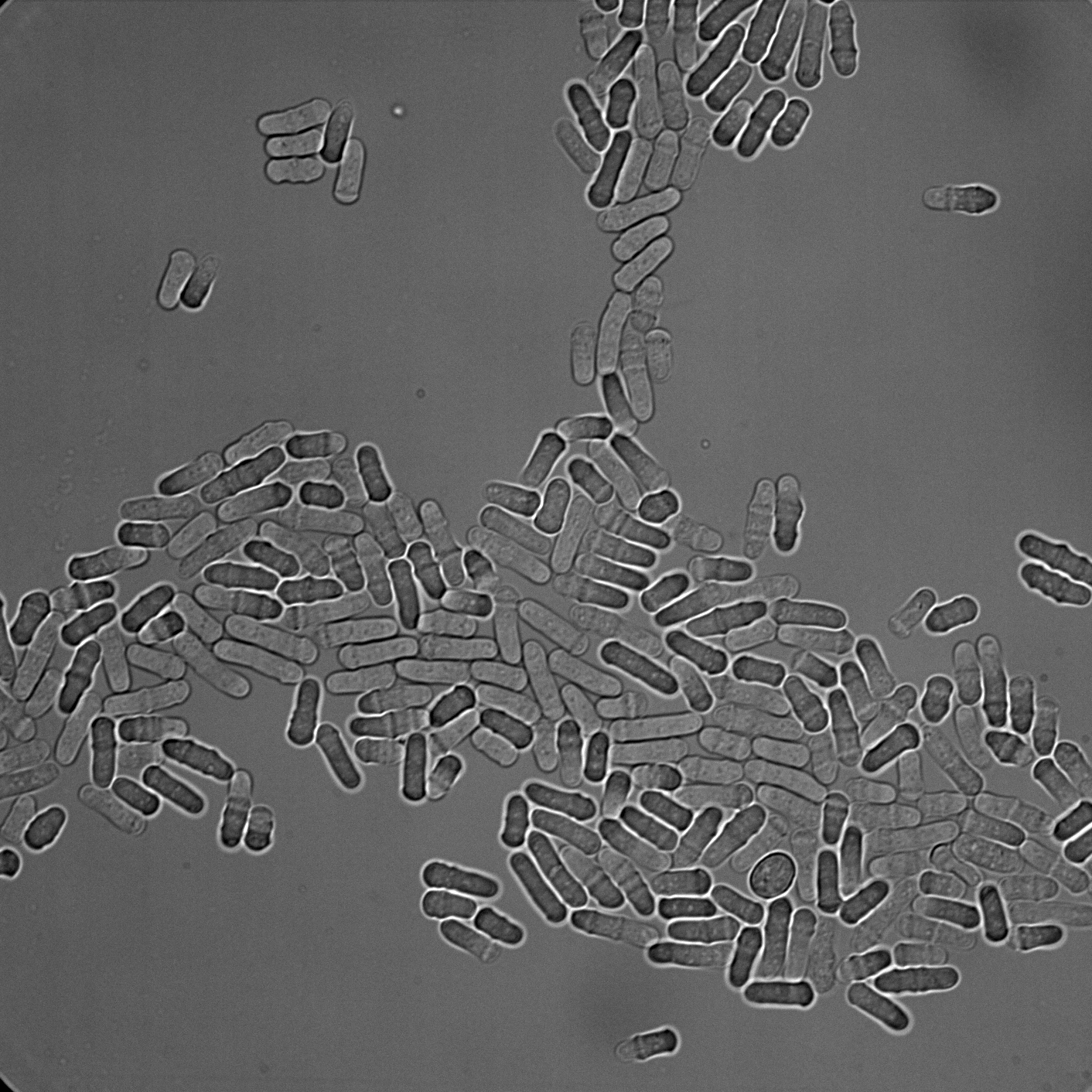

Supplement: Supplementary file 9 — Source data Fig. 5 [file 44318_2025_649_MOESM9_ESM.zip › 121174_Source_Data_Fig_5/Fig_5B/B5680_001-1_brightfield.tif]

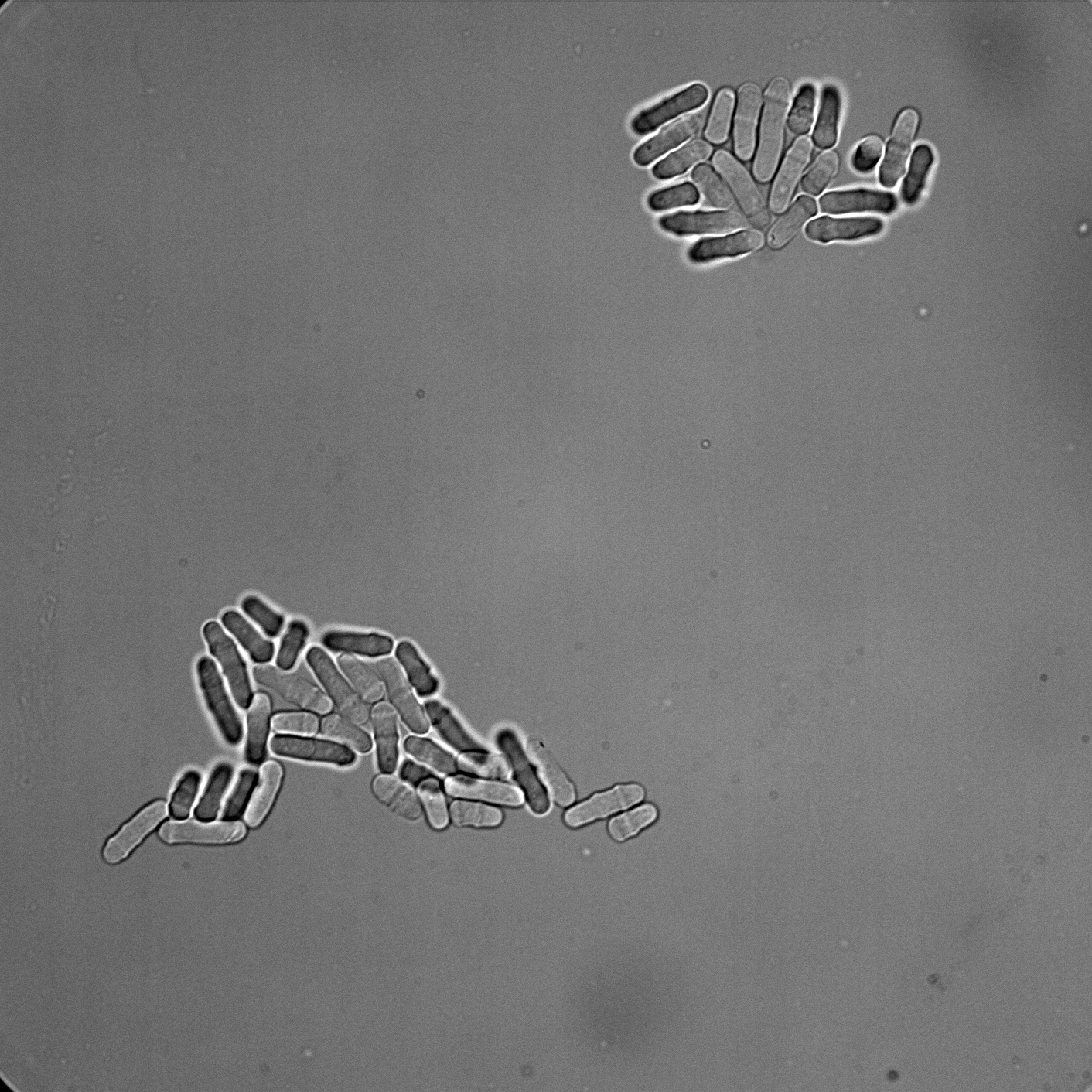

Supplement: Supplementary file 9 — Source data Fig. 5 [file 44318_2025_649_MOESM9_ESM.zip › 121174_Source_Data_Fig_5/Fig_5B/B5665_001-1_brightfield.tif]

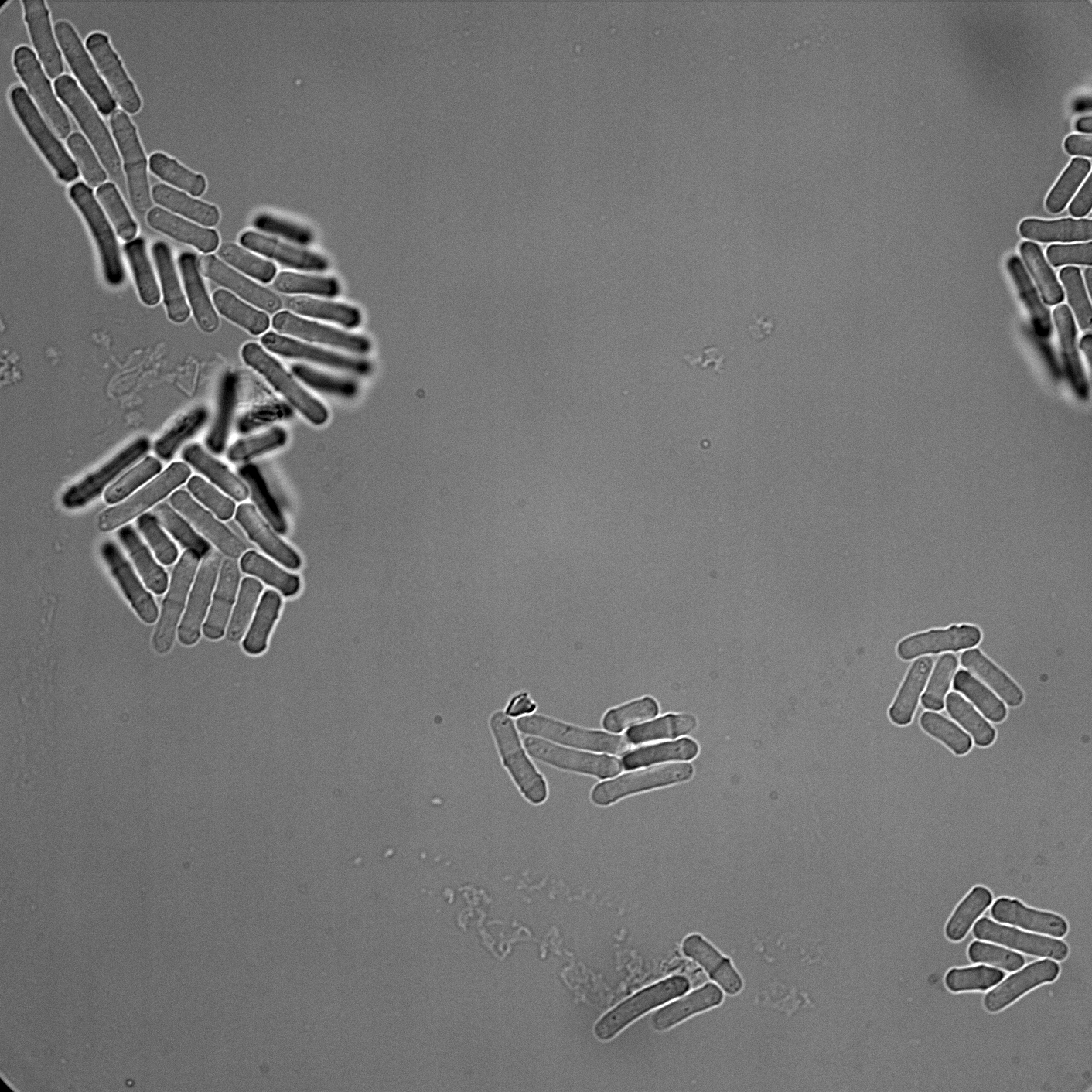

Supplement: Supplementary file 9 — Source data Fig. 5 [file 44318_2025_649_MOESM9_ESM.zip › 121174_Source_Data_Fig_5/Fig_5B/B5671_004-1_brightfield.tif]

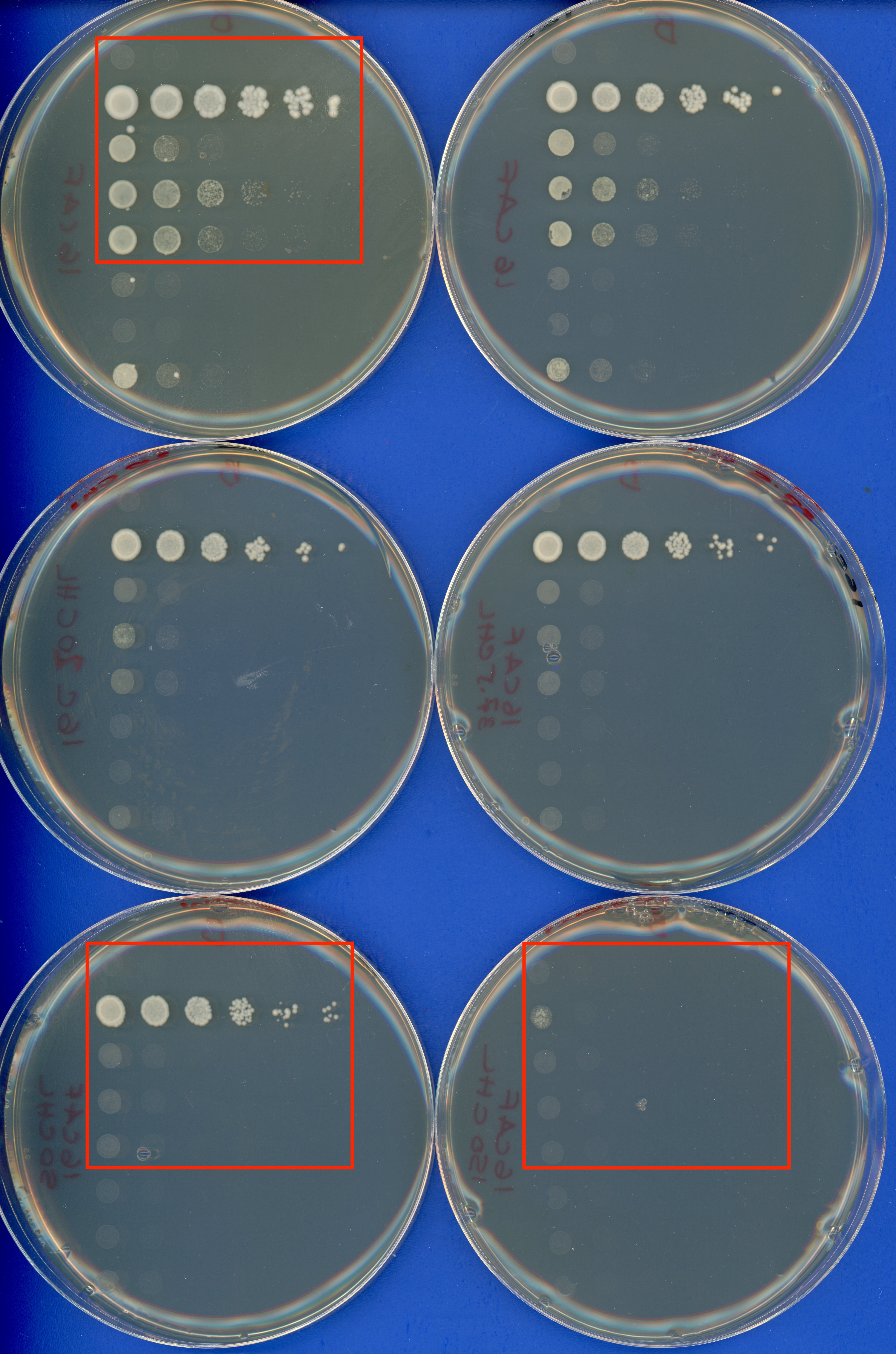

Supplement: Supplementary file 9 — Source data Fig. 5 [file 44318_2025_649_MOESM9_ESM.zip › 121174_Source_Data_Fig_5/Fig_5F/Fellas_Fig_5F_16CAF_0_50_150_CHL.jpg]

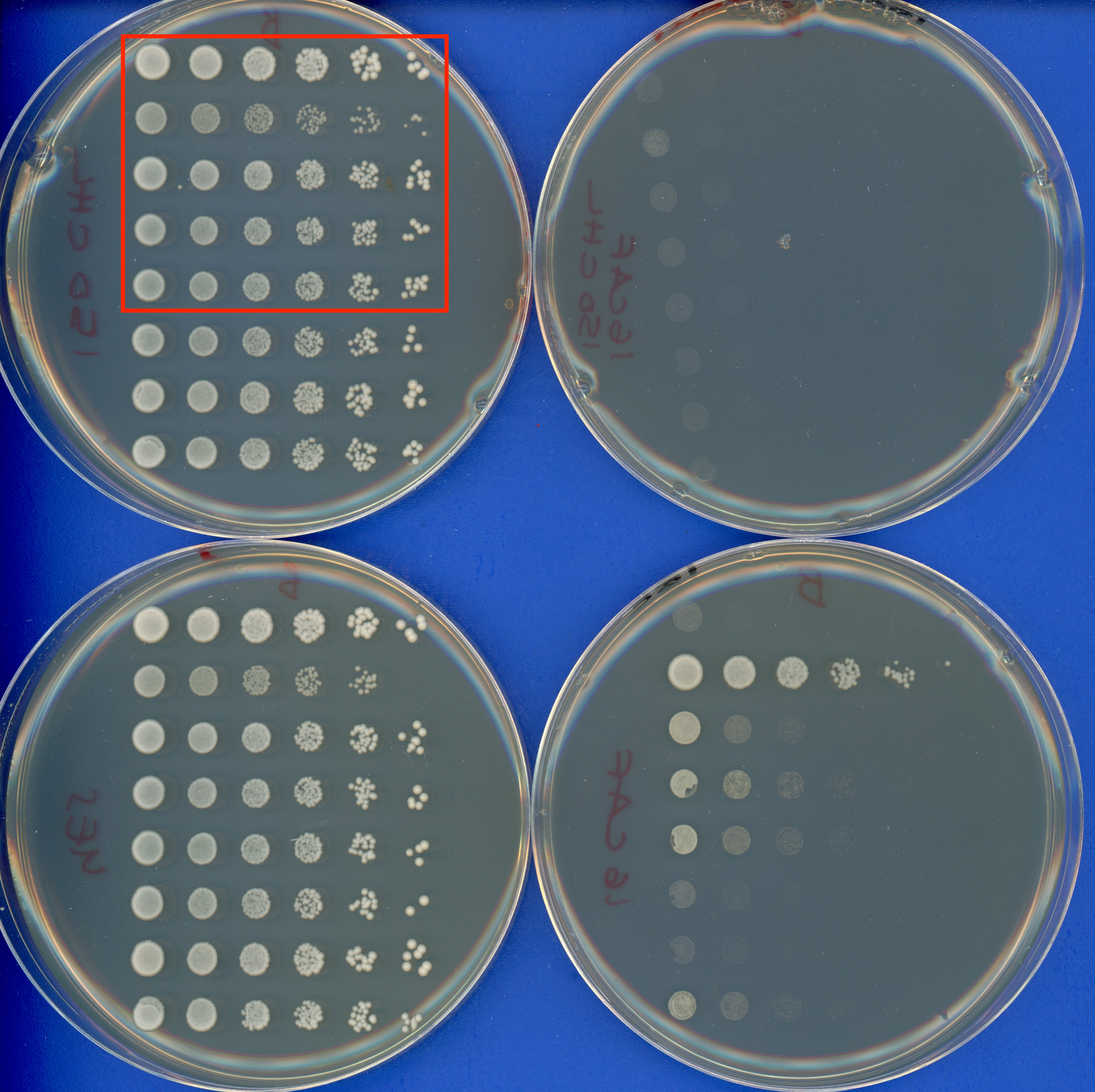

Supplement: Supplementary file 9 — Source data Fig. 5 [file 44318_2025_649_MOESM9_ESM.zip › 121174_Source_Data_Fig_5/Fig_5F/Fellas_Fig_5F_150CHL.jpg]

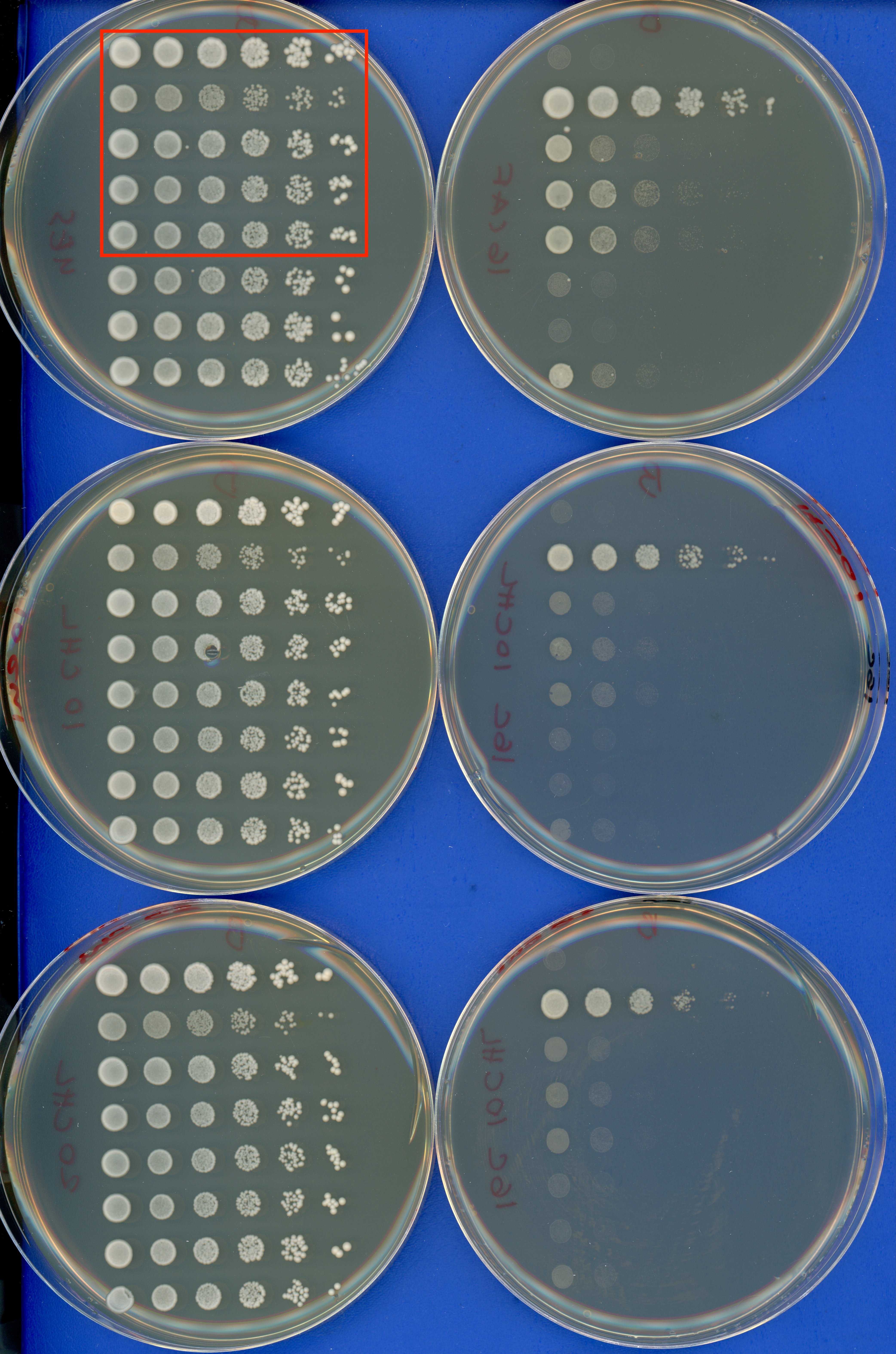

Supplement: Supplementary file 9 — Source data Fig. 5 [file 44318_2025_649_MOESM9_ESM.zip › 121174_Source_Data_Fig_5/Fig_5F/Fellas_Fig_5F_YES.jpg]

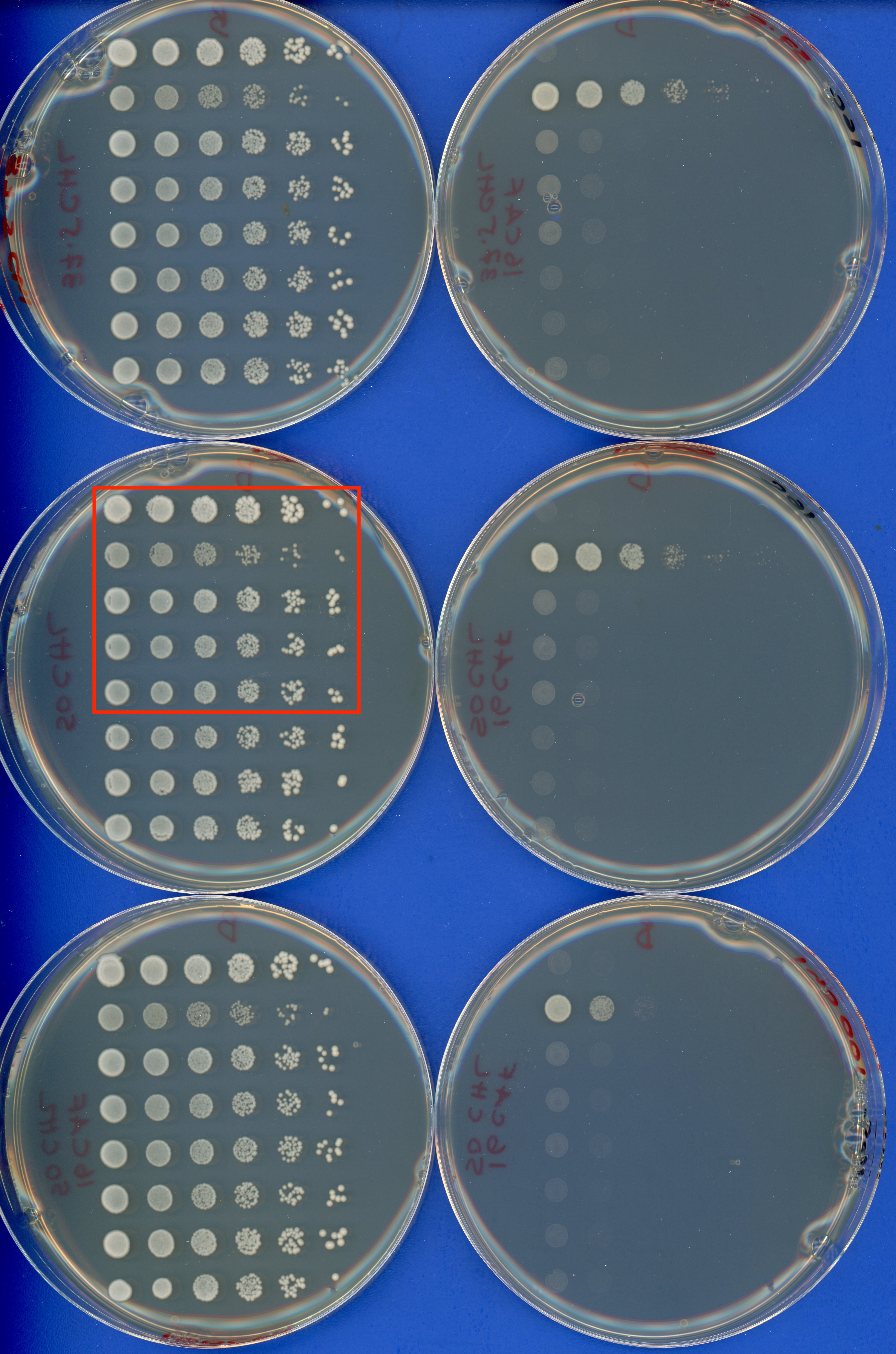

Supplement: Supplementary file 9 — Source data Fig. 5 [file 44318_2025_649_MOESM9_ESM.zip › 121174_Source_Data_Fig_5/Fig_5F/Fellas_Fig_5F_50CHL.jpg]

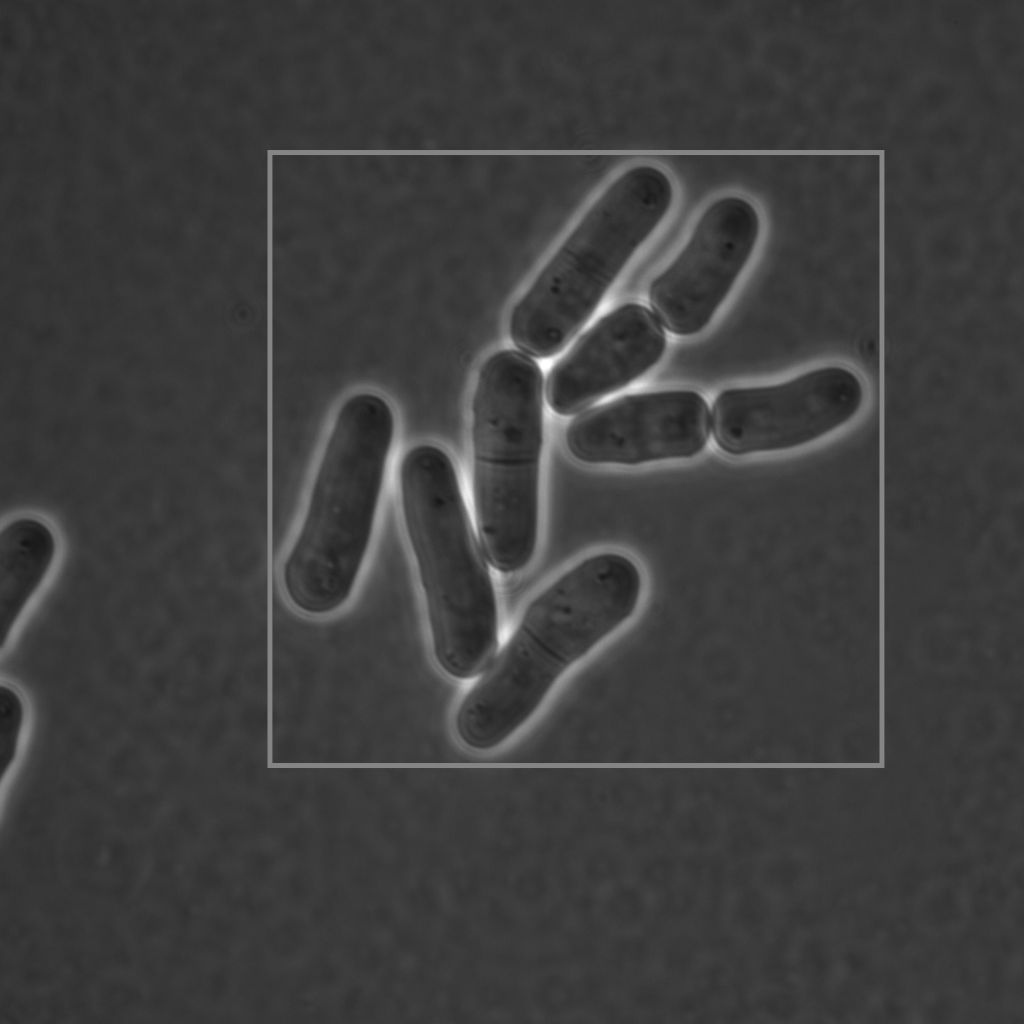

Supplement: Supplementary file 9 — Source data Fig. 5 [file 44318_2025_649_MOESM9_ESM.zip › 121174_Source_Data_Fig_5/Fig_5A/Fig5A_annotated/Fellas_Fig_5A_DIC_cup1_annotated.TIF]

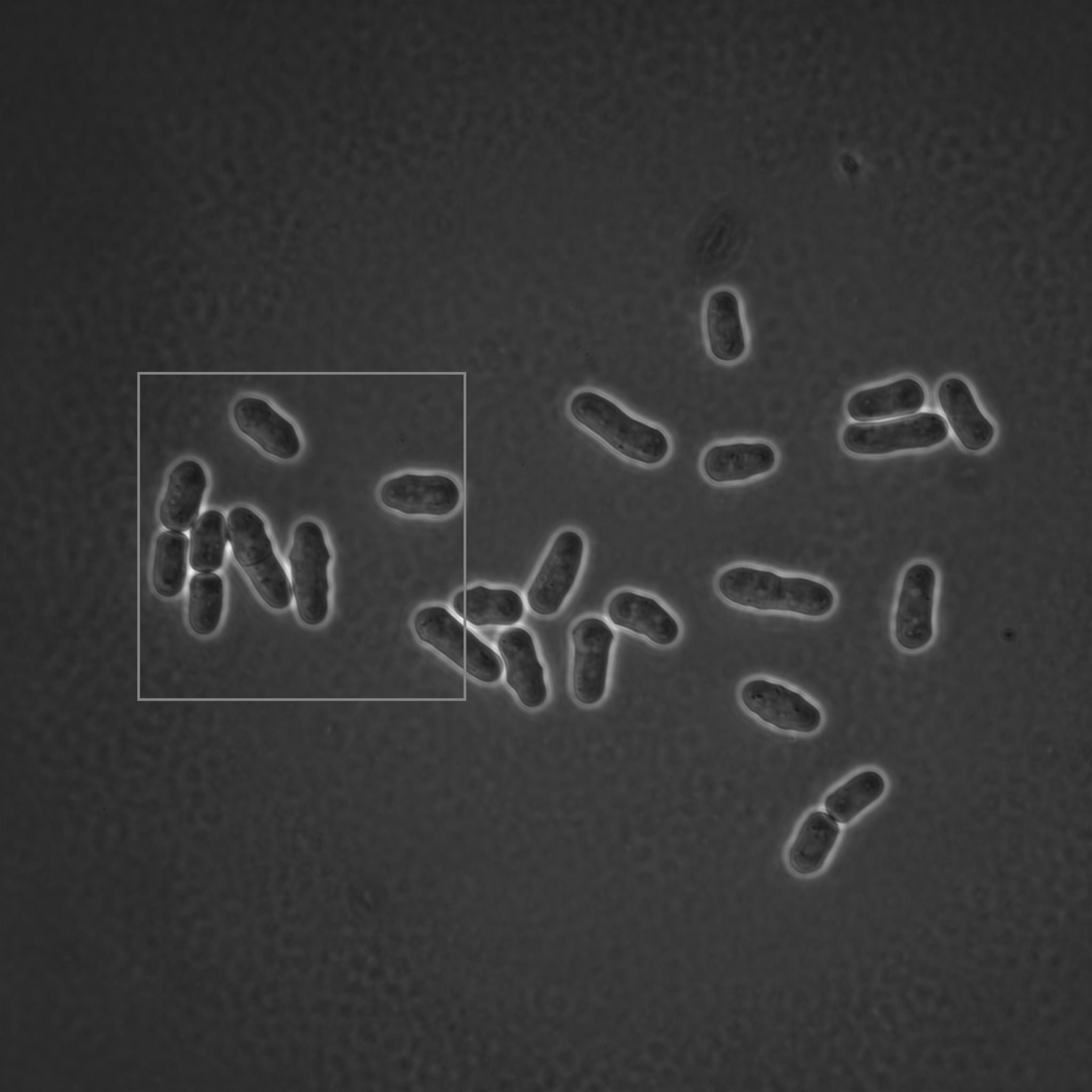

Supplement: Supplementary file 9 — Source data Fig. 5 [file 44318_2025_649_MOESM9_ESM.zip › 121174_Source_Data_Fig_5/Fig_5A/Fig5A_annotated/Fellas_Fig_5A_DIC_wt_annotated.TIF]

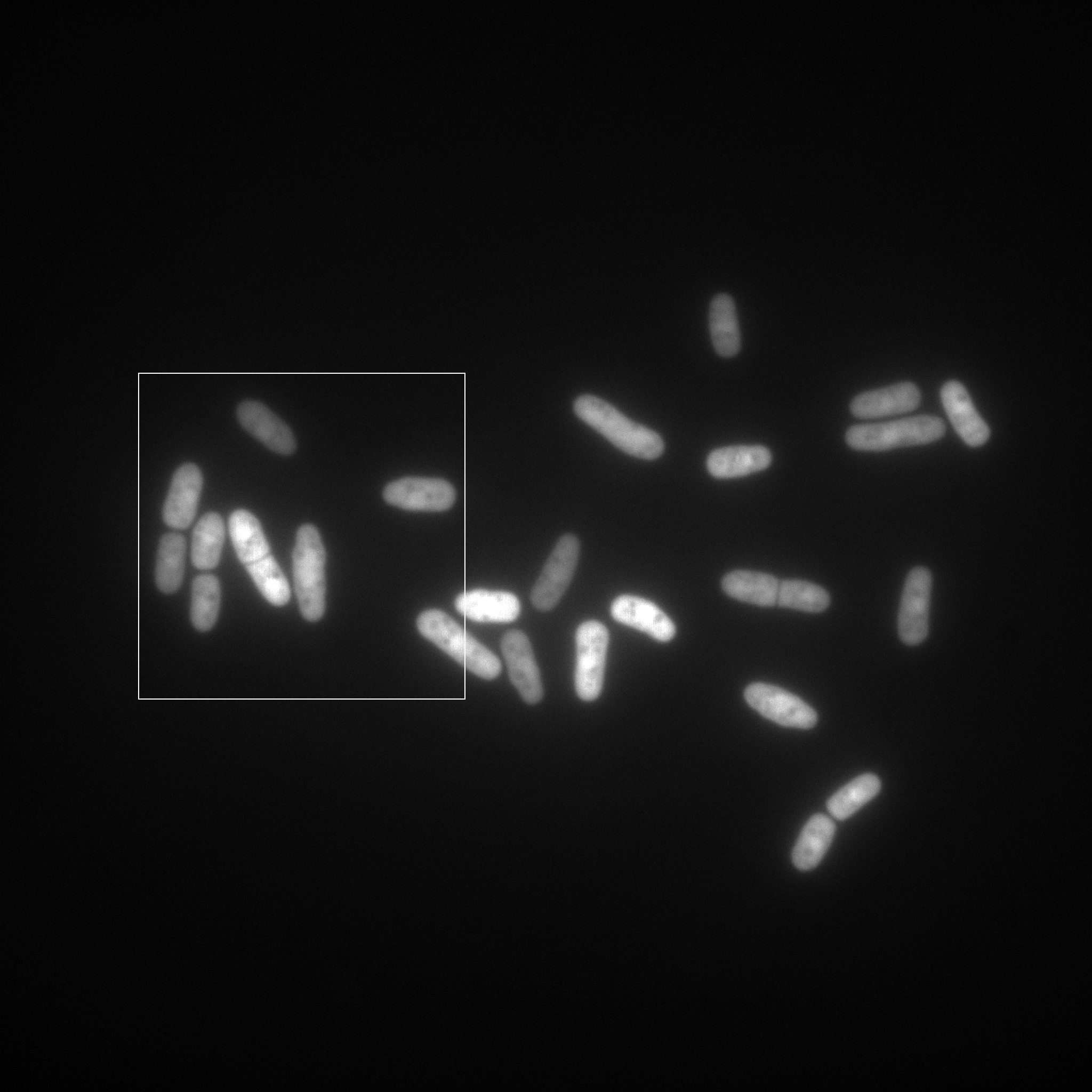

Supplement: Supplementary file 9 — Source data Fig. 5 [file 44318_2025_649_MOESM9_ESM.zip › 121174_Source_Data_Fig_5/Fig_5A/Fig5A_annotated/Fellas_Fig_5A_FITCGFP_wt_annotated.TIF]

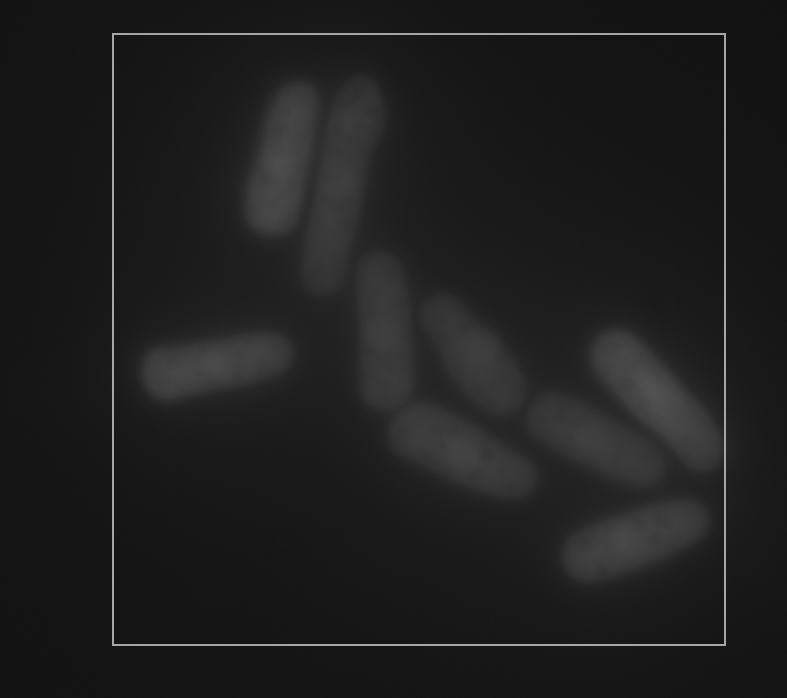

Supplement: Supplementary file 9 — Source data Fig. 5 [file 44318_2025_649_MOESM9_ESM.zip › 121174_Source_Data_Fig_5/Fig_5A/Fig5A_annotated/Fellas_Fig_5A_FITCGFP_qcr7_annotated_bright.TIF]

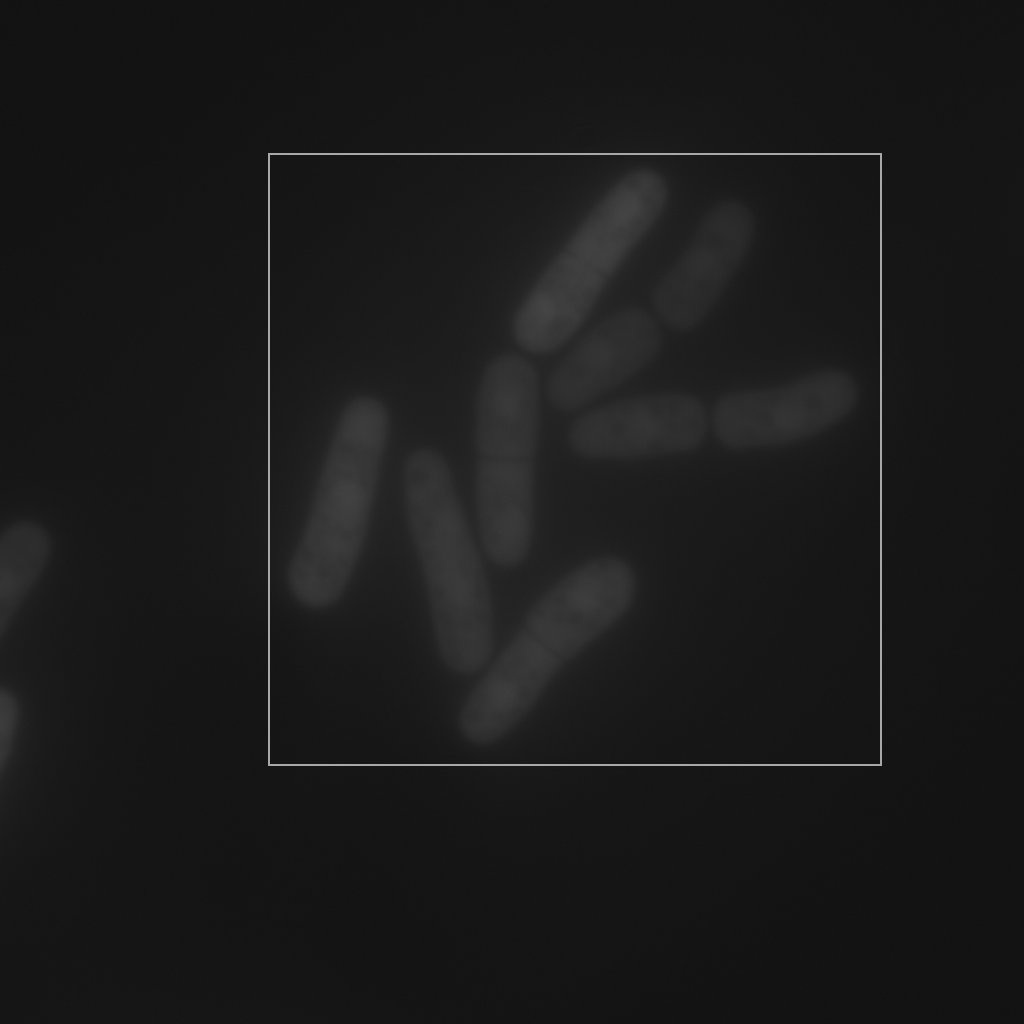

Supplement: Supplementary file 9 — Source data Fig. 5 [file 44318_2025_649_MOESM9_ESM.zip › 121174_Source_Data_Fig_5/Fig_5A/Fig5A_annotated/Fellas_Fig_5A_FITCGFP_cup1_annotated_bright.TIF]

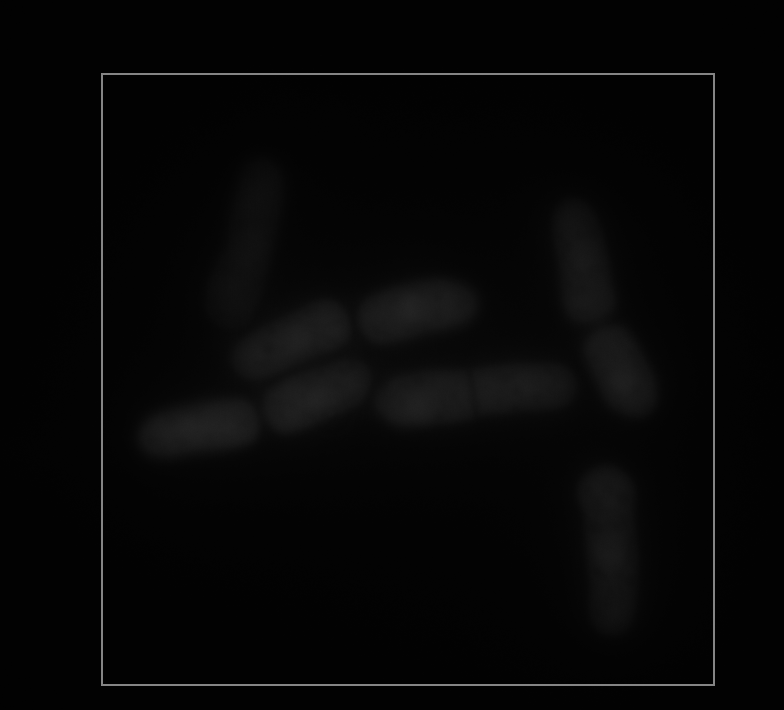

Supplement: Supplementary file 9 — Source data Fig. 5 [file 44318_2025_649_MOESM9_ESM.zip › 121174_Source_Data_Fig_5/Fig_5A/Fig5A_annotated/Fellas_Fig_5A_FITCGFP_ppr4_annotated.TIF]

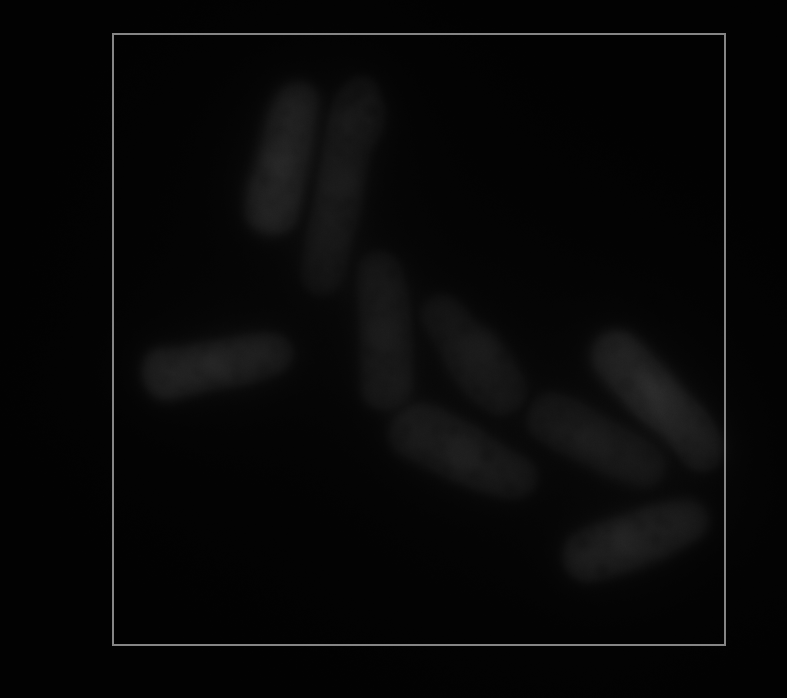

Supplement: Supplementary file 9 — Source data Fig. 5 [file 44318_2025_649_MOESM9_ESM.zip › 121174_Source_Data_Fig_5/Fig_5A/Fig5A_annotated/Fellas_Fig_5A_FITCGFP_qcr7_annotated.TIF]

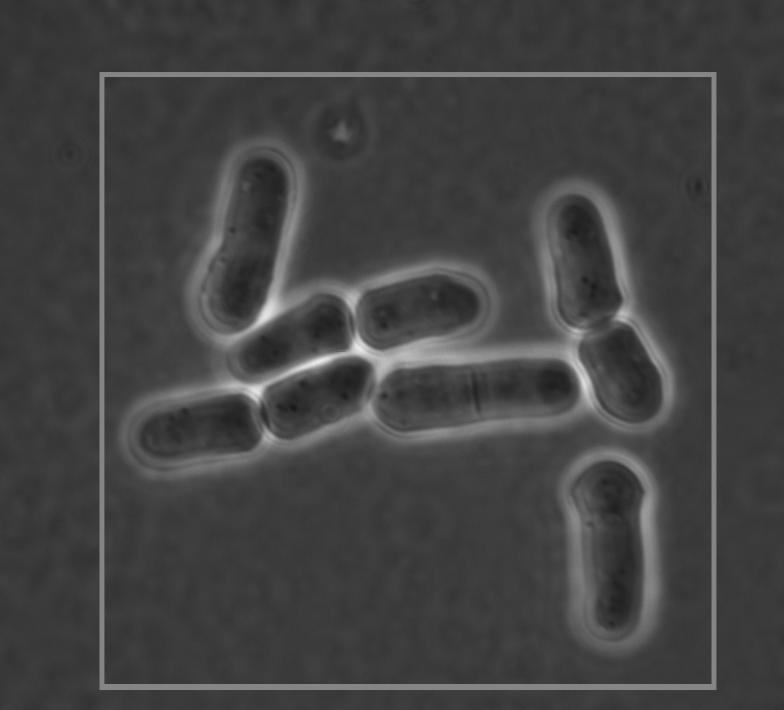

Supplement: Supplementary file 9 — Source data Fig. 5 [file 44318_2025_649_MOESM9_ESM.zip › 121174_Source_Data_Fig_5/Fig_5A/Fig5A_annotated/Fellas_Fig_5A_DIC_ppr4_annotated.TIF]

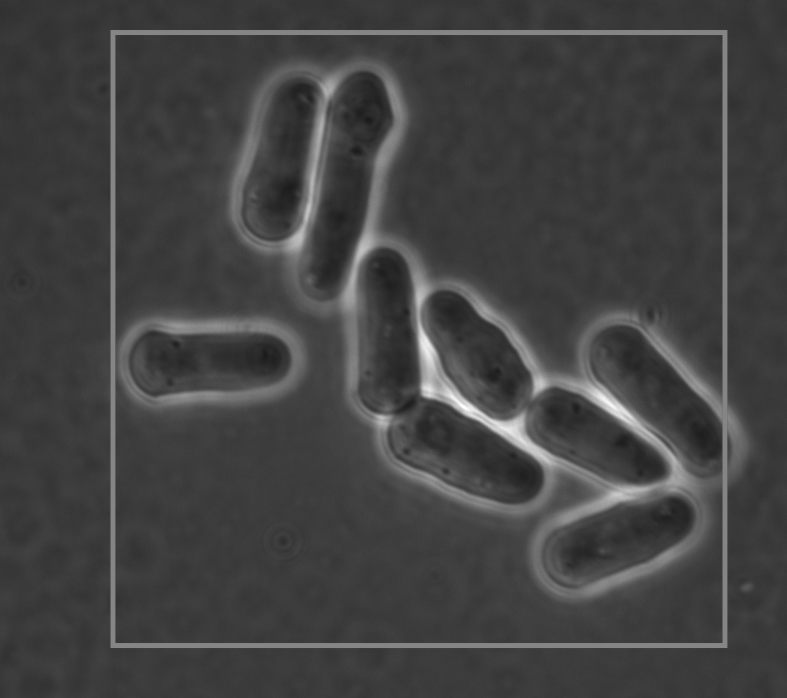

Supplement: Supplementary file 9 — Source data Fig. 5 [file 44318_2025_649_MOESM9_ESM.zip › 121174_Source_Data_Fig_5/Fig_5A/Fig5A_annotated/Fellas_Fig_5A_DIC_qcr7_annotated.TIF]

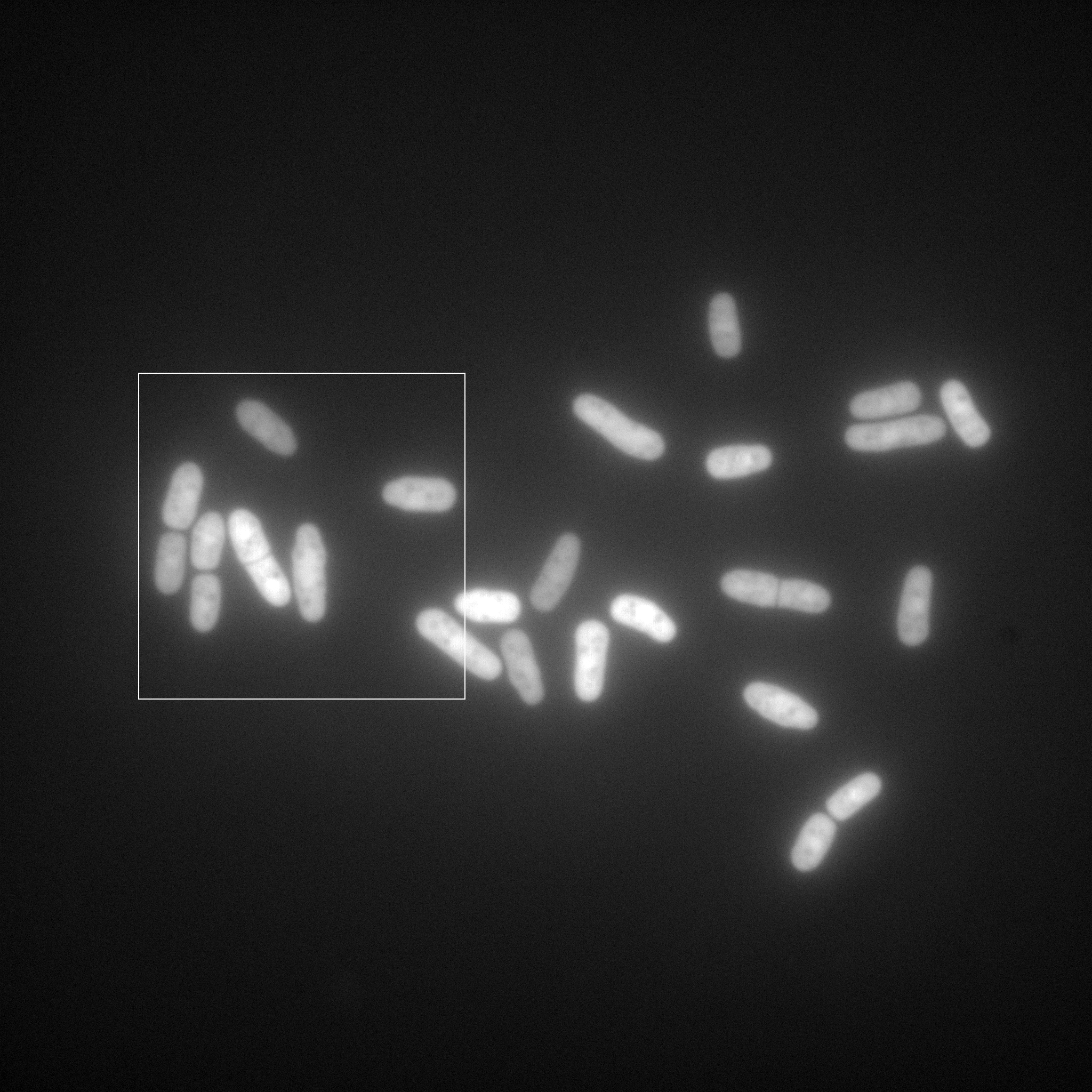

Supplement: Supplementary file 9 — Source data Fig. 5 [file 44318_2025_649_MOESM9_ESM.zip › 121174_Source_Data_Fig_5/Fig_5A/Fig5A_annotated/Fellas_Fig_5A_FITCGFP_wt_annotated_bright.TIF]

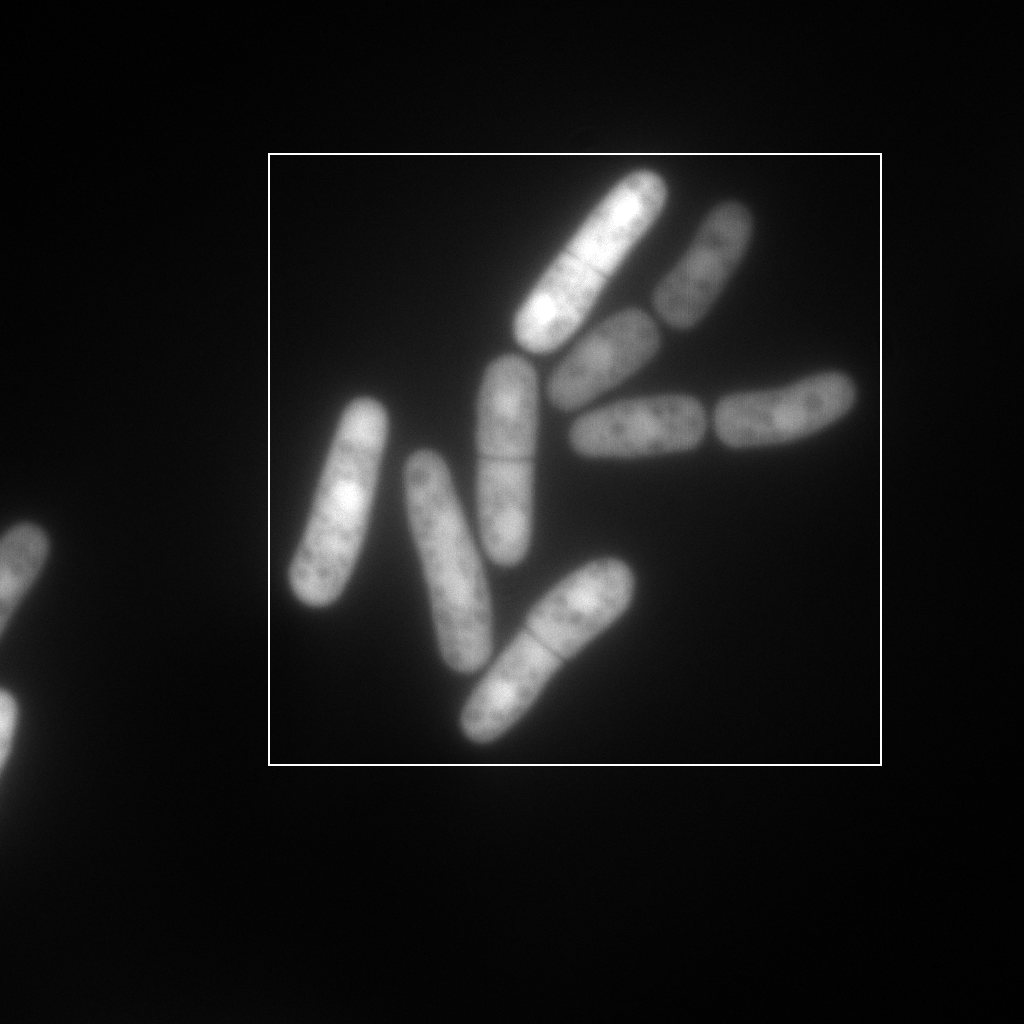

Supplement: Supplementary file 9 — Source data Fig. 5 [file 44318_2025_649_MOESM9_ESM.zip › 121174_Source_Data_Fig_5/Fig_5A/Fig5A_annotated/Fellas_Fig_5A_FITCGFP_cup1_annotated.TIF]

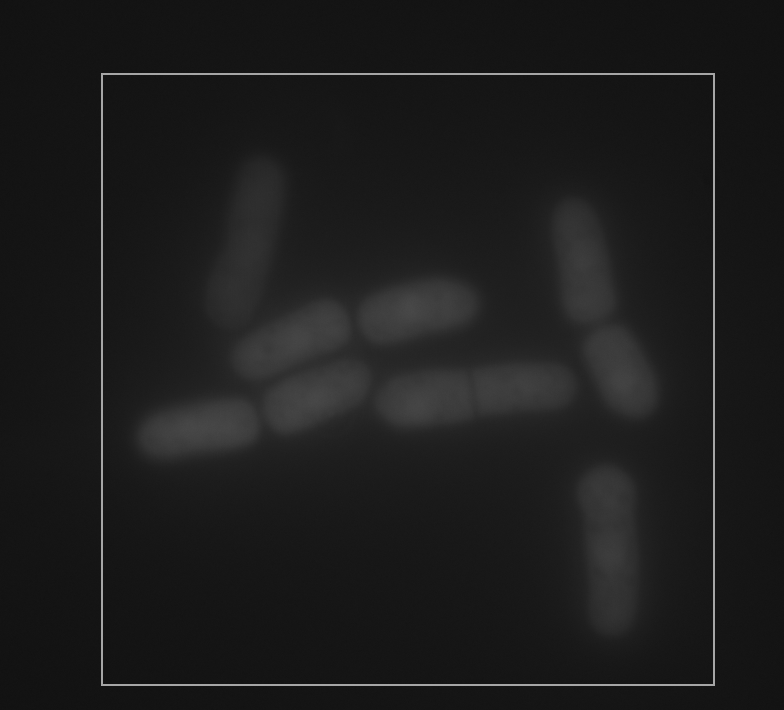

Supplement: Supplementary file 9 — Source data Fig. 5 [file 44318_2025_649_MOESM9_ESM.zip › 121174_Source_Data_Fig_5/Fig_5A/Fig5A_annotated/Fellas_Fig_5A_FITCGFP_ppr4_annotated_bright.TIF]

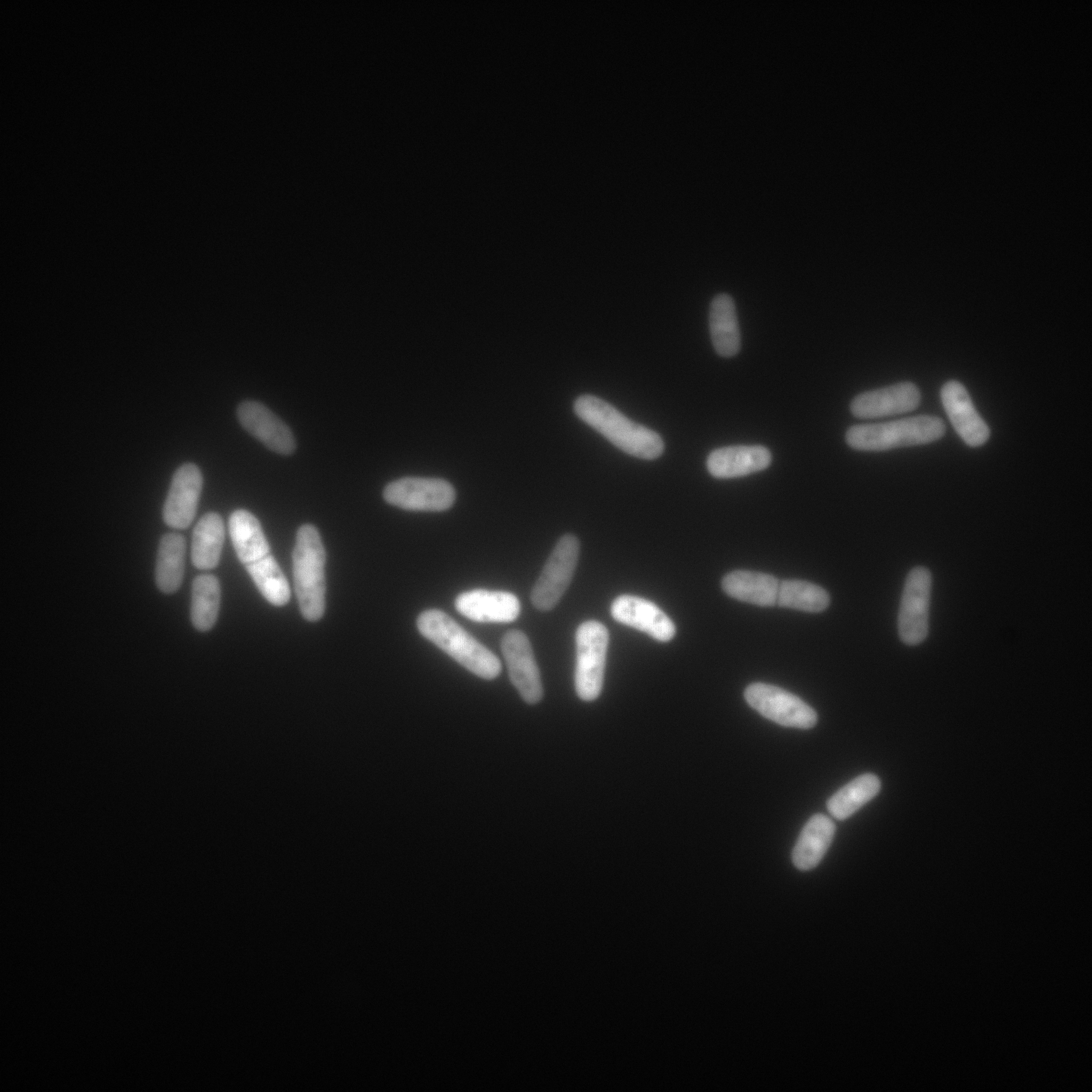

Supplement: Supplementary file 9 — Source data Fig. 5 [file 44318_2025_649_MOESM9_ESM.zip › 121174_Source_Data_Fig_5/Fig_5A/Fig5A_original_TIF_files/obr1pro-GFP_B6088-wt_006_w2FITC copy.TIF]

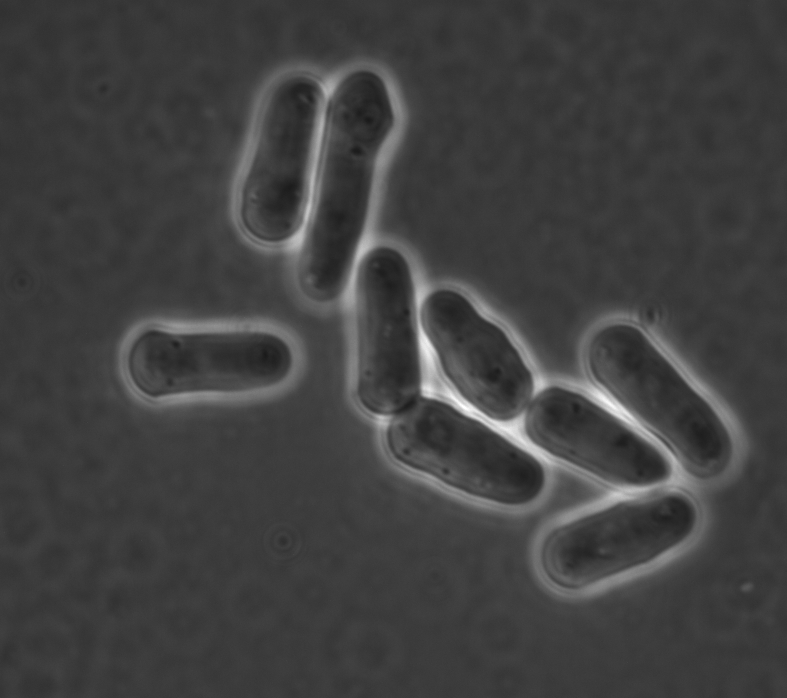

Supplement: Supplementary file 9 — Source data Fig. 5 [file 44318_2025_649_MOESM9_ESM.zip › 121174_Source_Data_Fig_5/Fig_5A/Fig5A_original_TIF_files/obr1pro-GFP_B8312-qcr7_001_w1DIC.TIF]

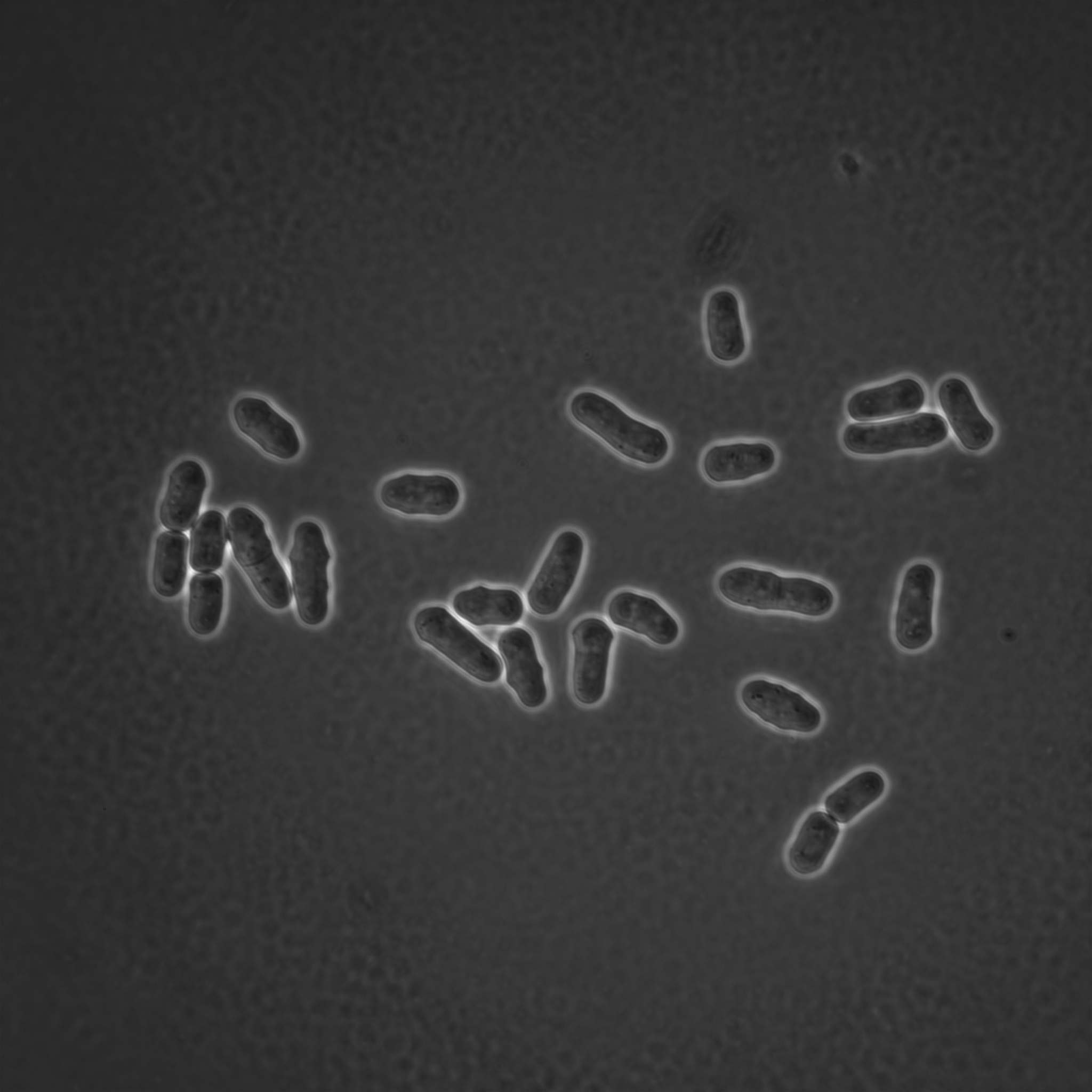

Supplement: Supplementary file 9 — Source data Fig. 5 [file 44318_2025_649_MOESM9_ESM.zip › 121174_Source_Data_Fig_5/Fig_5A/Fig5A_original_TIF_files/obr1pro-GFP_B6088-wt_006_w1DIC.TIF]

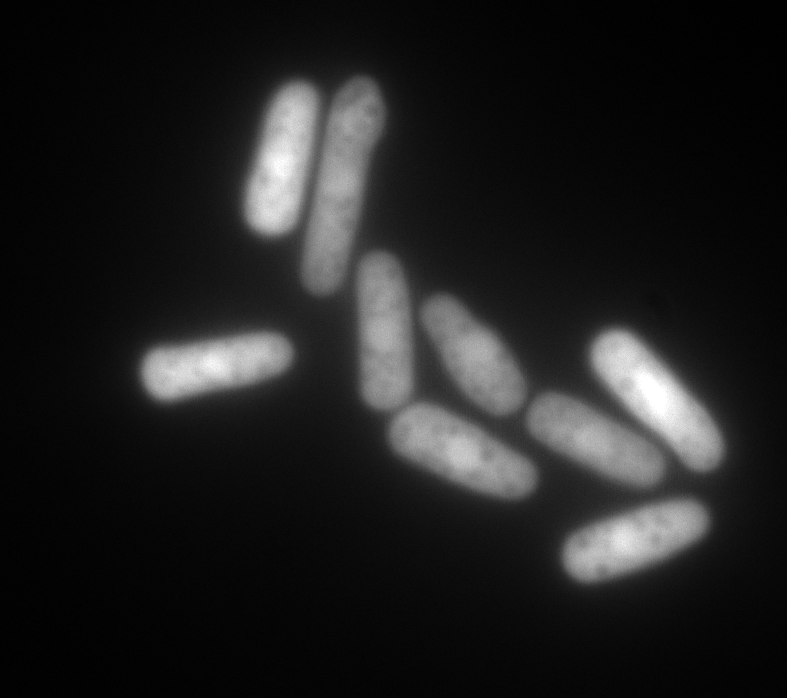

Supplement: Supplementary file 9 — Source data Fig. 5 [file 44318_2025_649_MOESM9_ESM.zip › 121174_Source_Data_Fig_5/Fig_5A/Fig5A_original_TIF_files/obr1pro-GFP_B8312-qcr7_001_w2FITC copy.TIF]

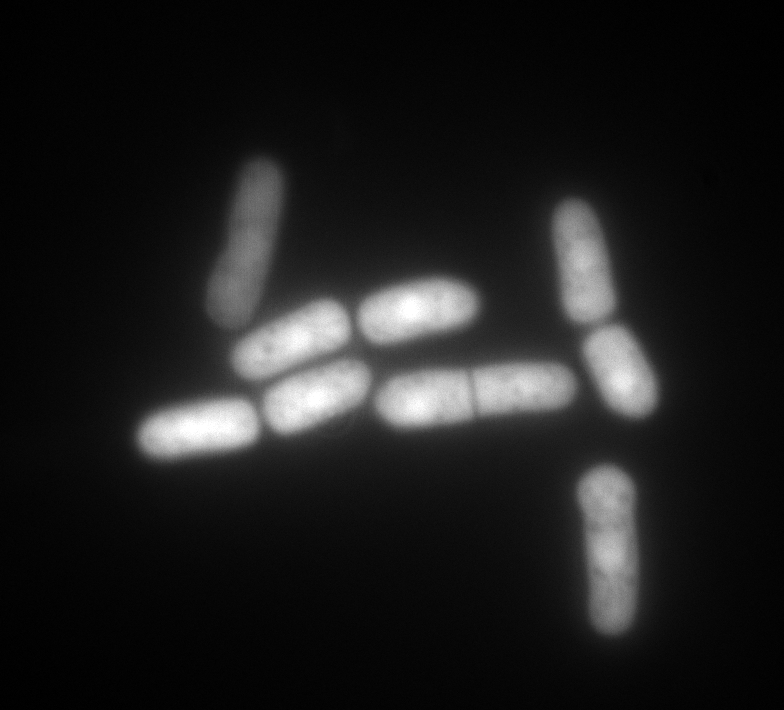

Supplement: Supplementary file 9 — Source data Fig. 5 [file 44318_2025_649_MOESM9_ESM.zip › 121174_Source_Data_Fig_5/Fig_5A/Fig5A_original_TIF_files/obr1pro-GFP_B6354-ppr4_002_w2FITC copy.TIF]

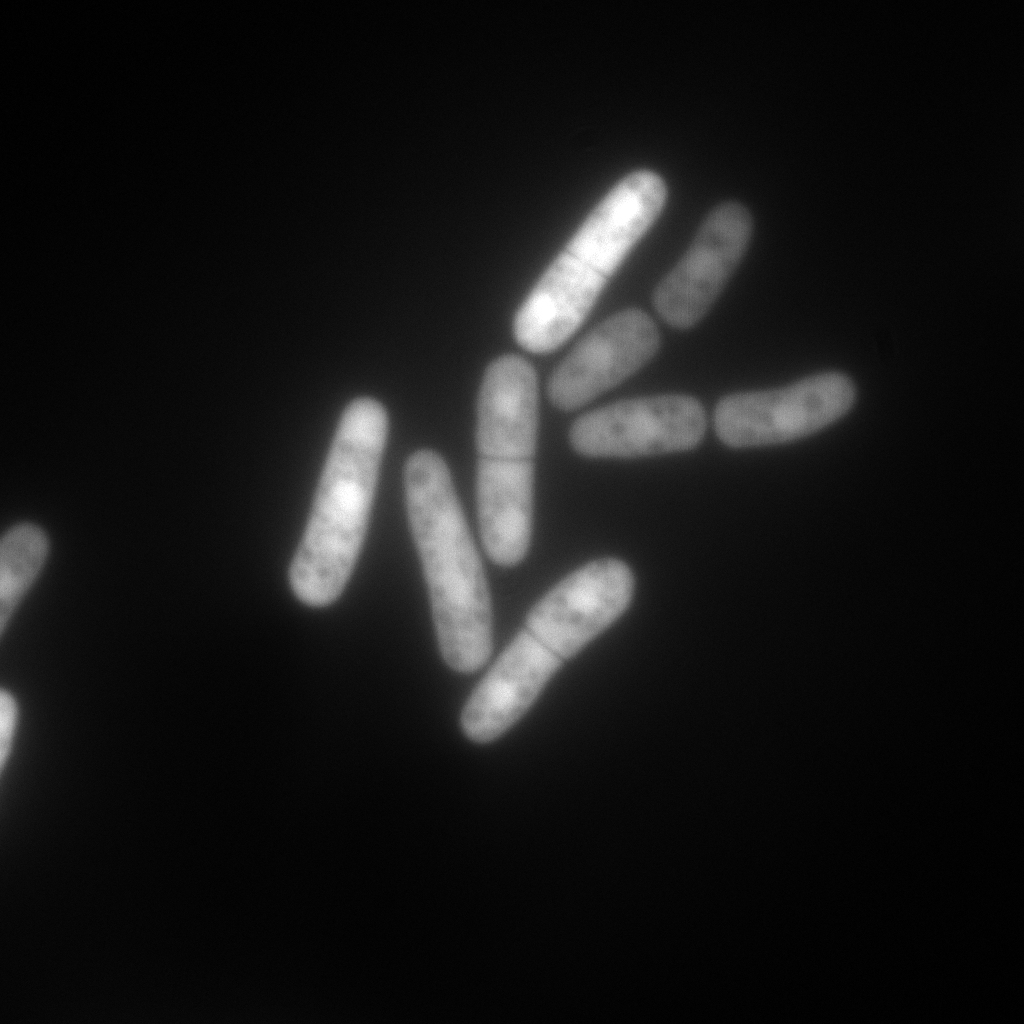

Supplement: Supplementary file 9 — Source data Fig. 5 [file 44318_2025_649_MOESM9_ESM.zip › 121174_Source_Data_Fig_5/Fig_5A/Fig5A_original_TIF_files/obr1pro-GFP_B8250-cup1_008_w2FITC copy.TIF]

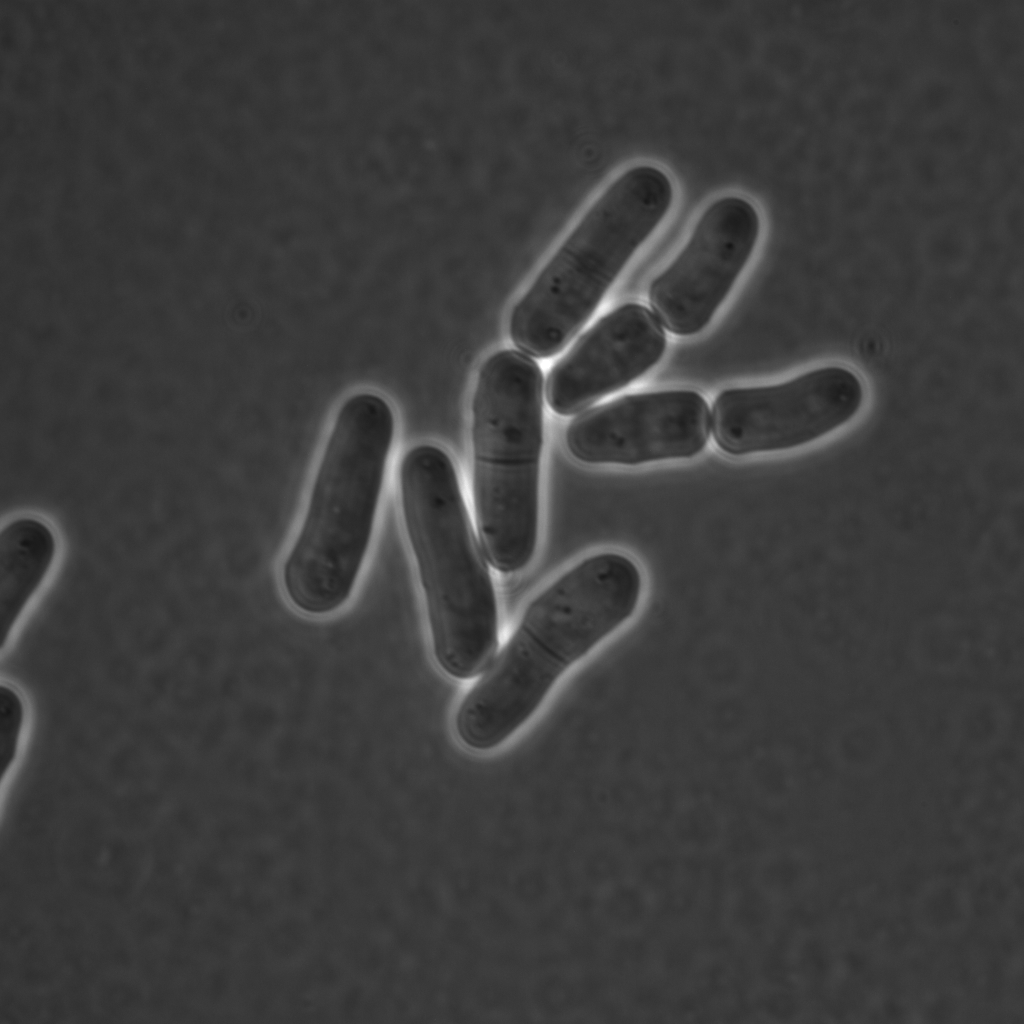

Supplement: Supplementary file 9 — Source data Fig. 5 [file 44318_2025_649_MOESM9_ESM.zip › 121174_Source_Data_Fig_5/Fig_5A/Fig5A_original_TIF_files/obr1pro-GFP_B8250-cup1_008_w1DIC.TIF]

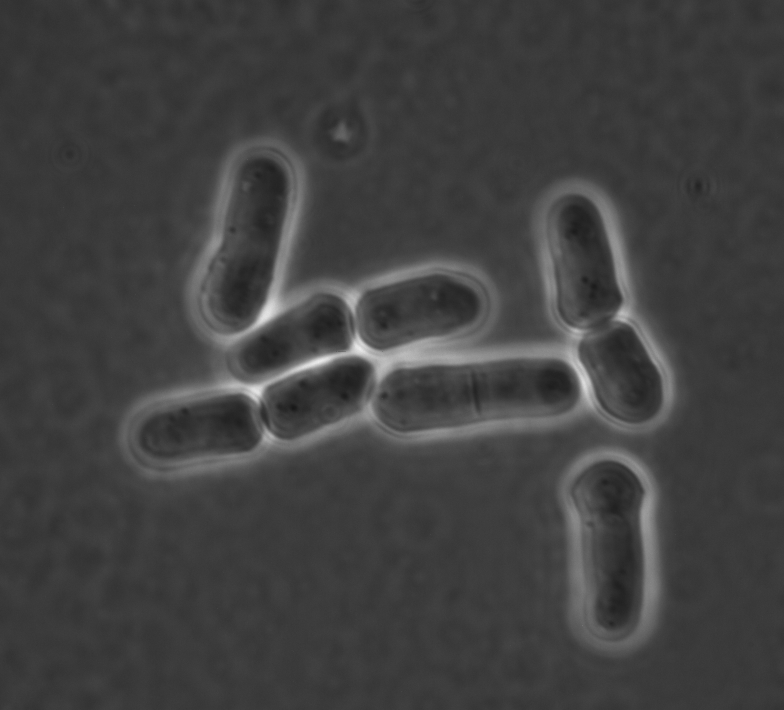

Supplement: Supplementary file 9 — Source data Fig. 5 [file 44318_2025_649_MOESM9_ESM.zip › 121174_Source_Data_Fig_5/Fig_5A/Fig5A_original_TIF_files/obr1pro-GFP_B6354-ppr4_002_w1DIC.TIF]

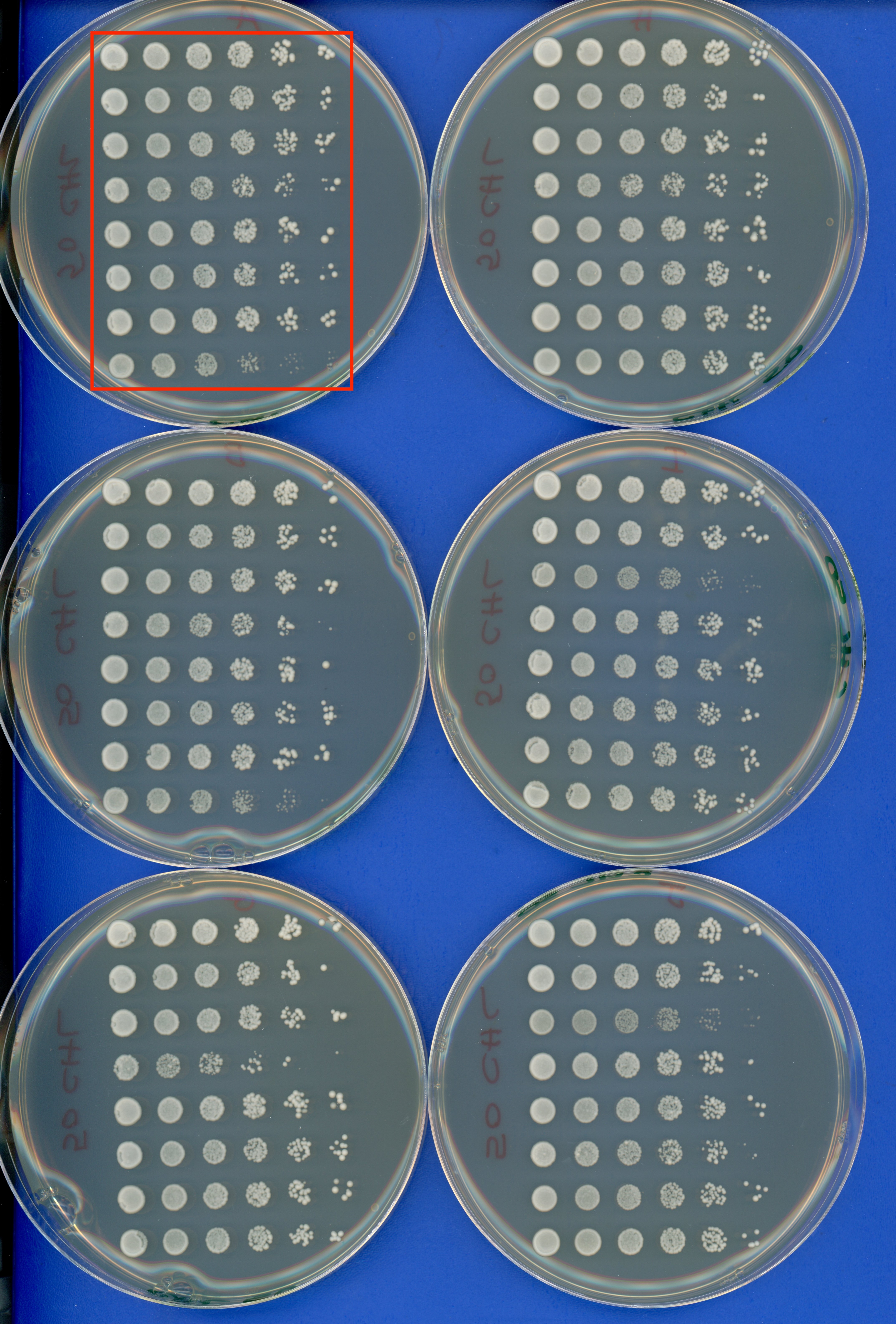

Supplement: Supplementary file 10 — Source data Fig. 6 [file 44318_2025_649_MOESM10_ESM.zip › 121174_Source_Data_Fig_6/Fig_6E/Fellas_Fig_6E_50CHL_annotated.jpg]

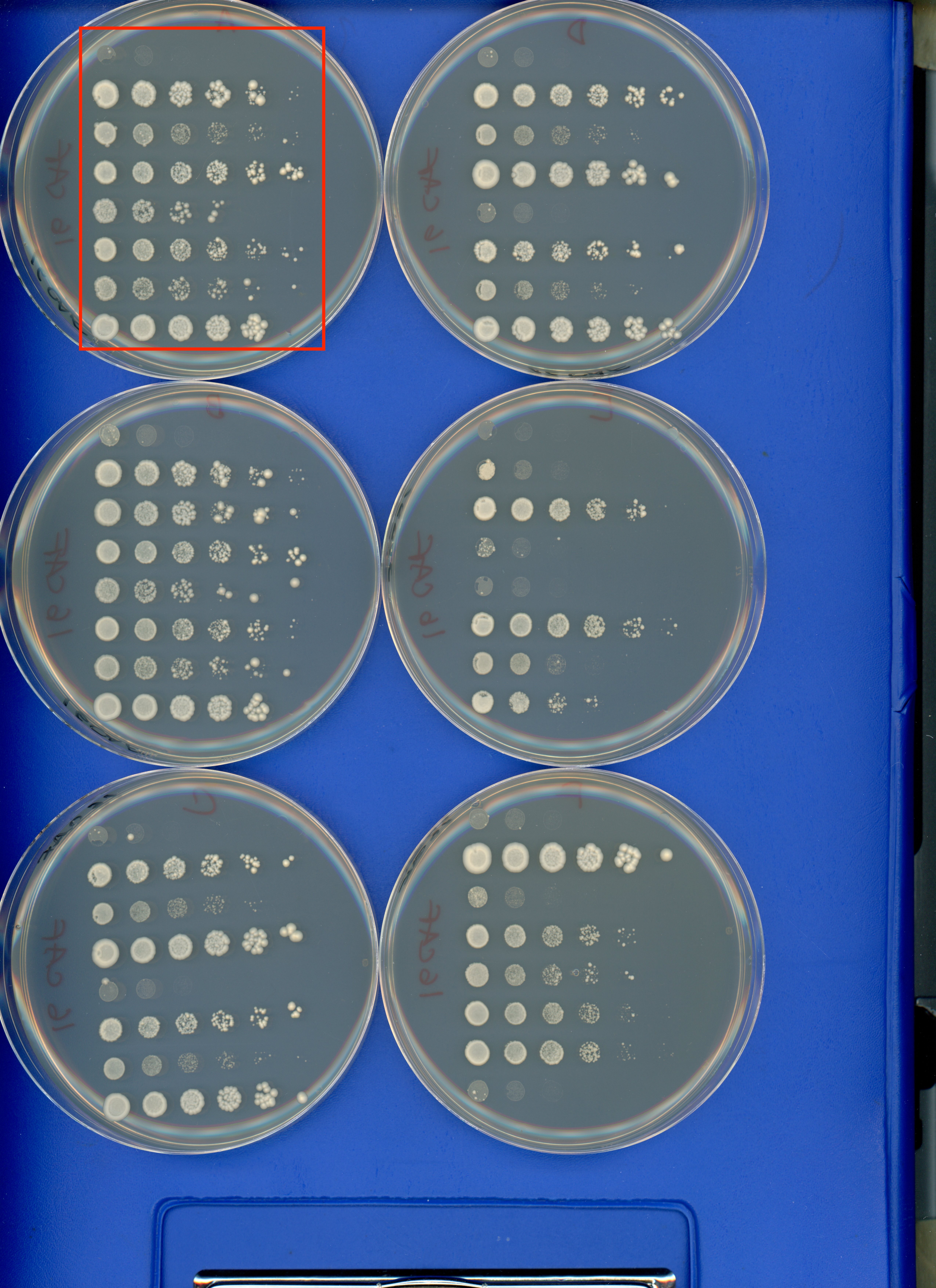

Supplement: Supplementary file 10 — Source data Fig. 6 [file 44318_2025_649_MOESM10_ESM.zip › 121174_Source_Data_Fig_6/Fig_6E/Fellas_Fig_6E_16CAF_annotated.jpg]

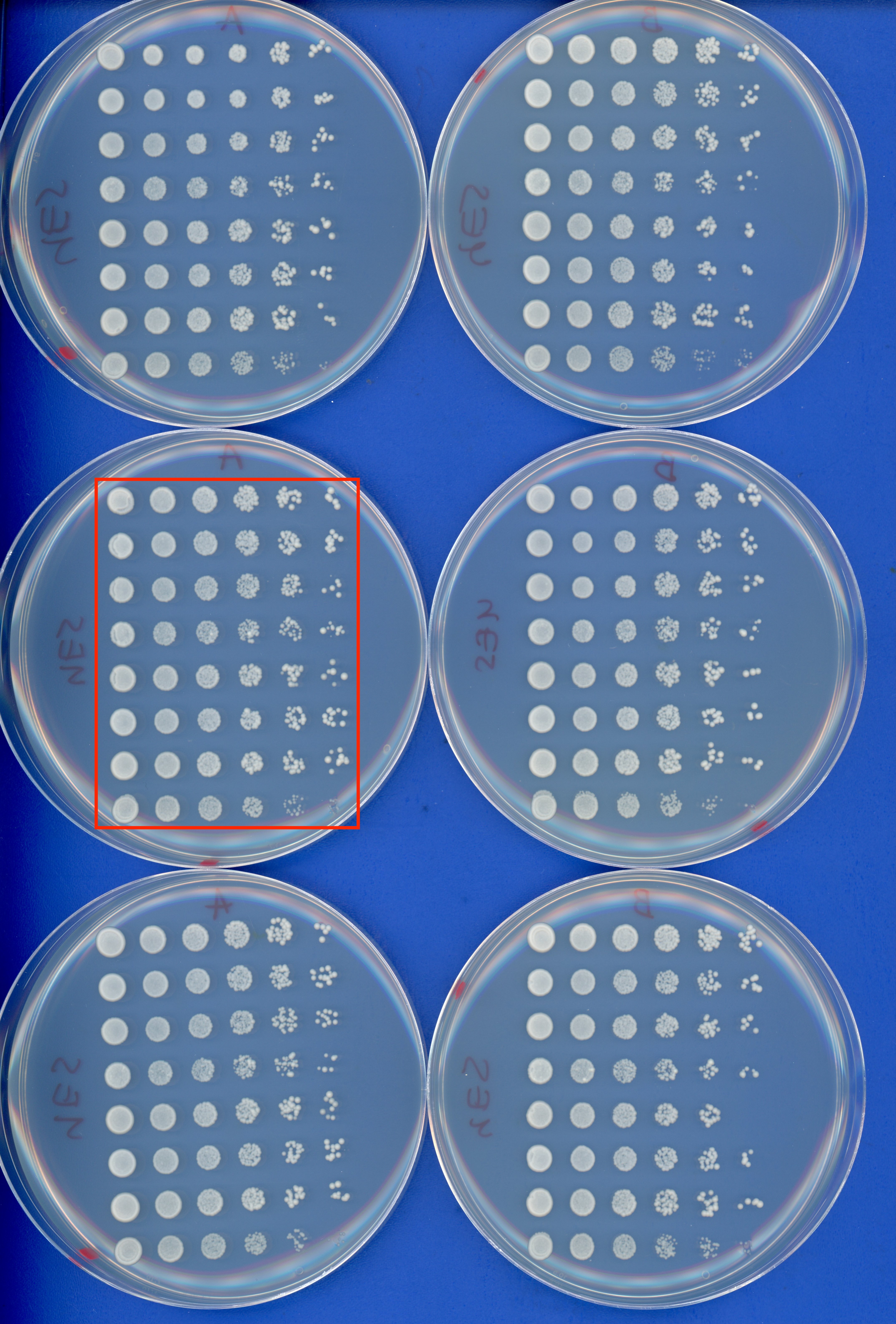

Supplement: Supplementary file 10 — Source data Fig. 6 [file 44318_2025_649_MOESM10_ESM.zip › 121174_Source_Data_Fig_6/Fig_6E/Fellas_Fig_6E_YES_annotated.jpg]

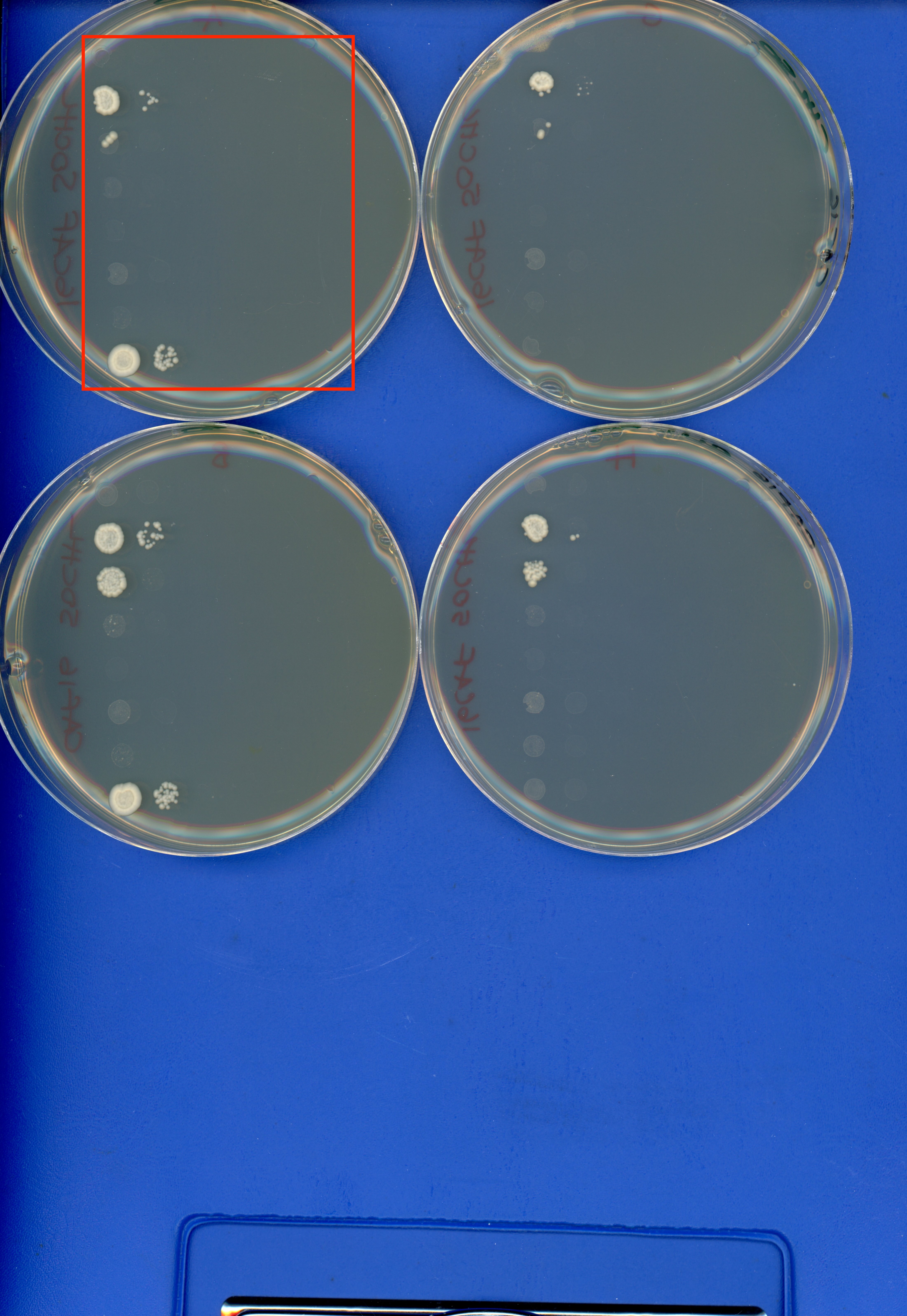

Supplement: Supplementary file 10 — Source data Fig. 6 [file 44318_2025_649_MOESM10_ESM.zip › 121174_Source_Data_Fig_6/Fig_6E/Fellas_Fig_6E_16CAF_50CHL_annotated.jpg]

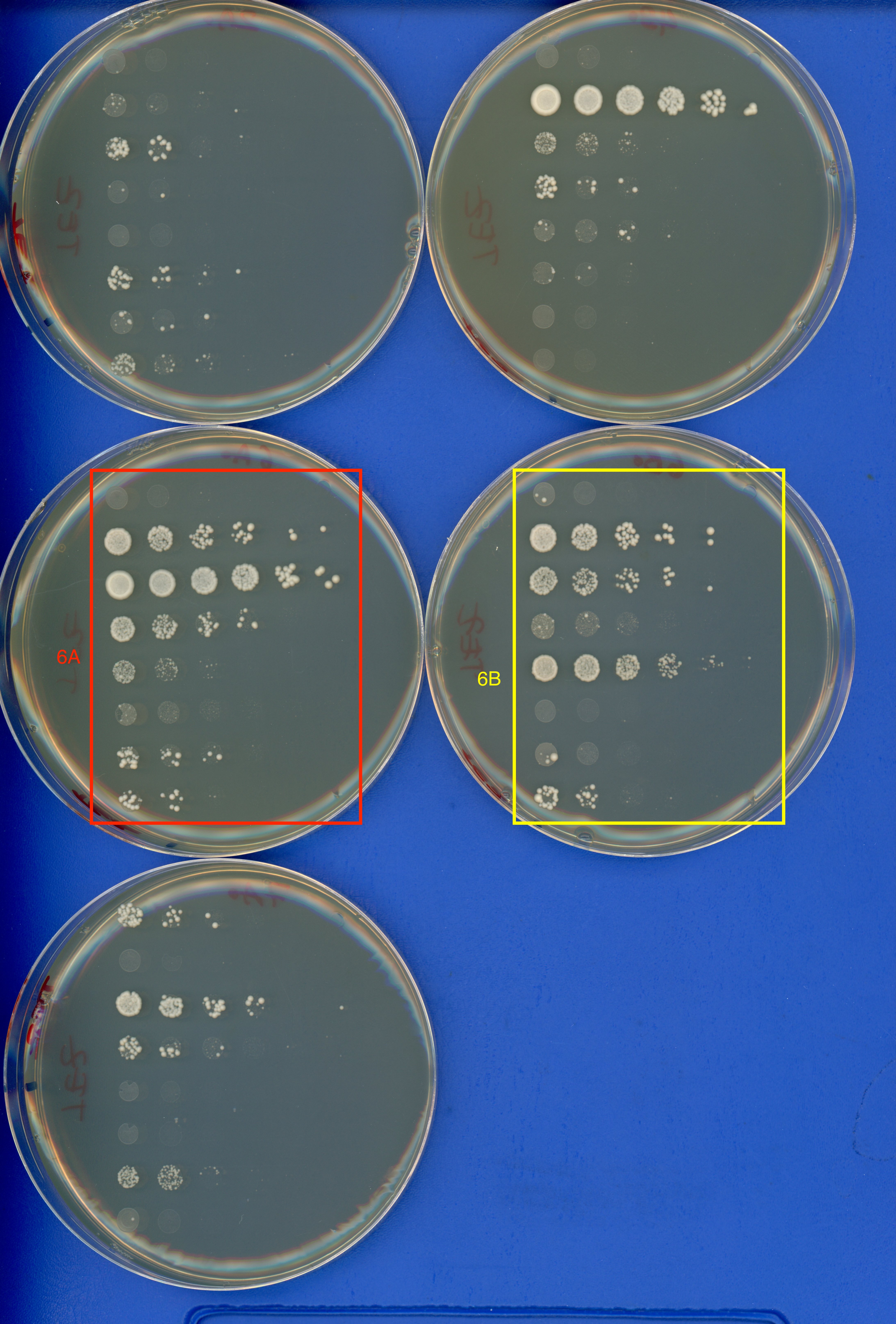

Supplement: Supplementary file 10 — Source data Fig. 6 [file 44318_2025_649_MOESM10_ESM.zip › 121174_Source_Data_Fig_6/Fig_6A_6B/Fellas_Fig_6A_6B_TEB_annotated.jpg]

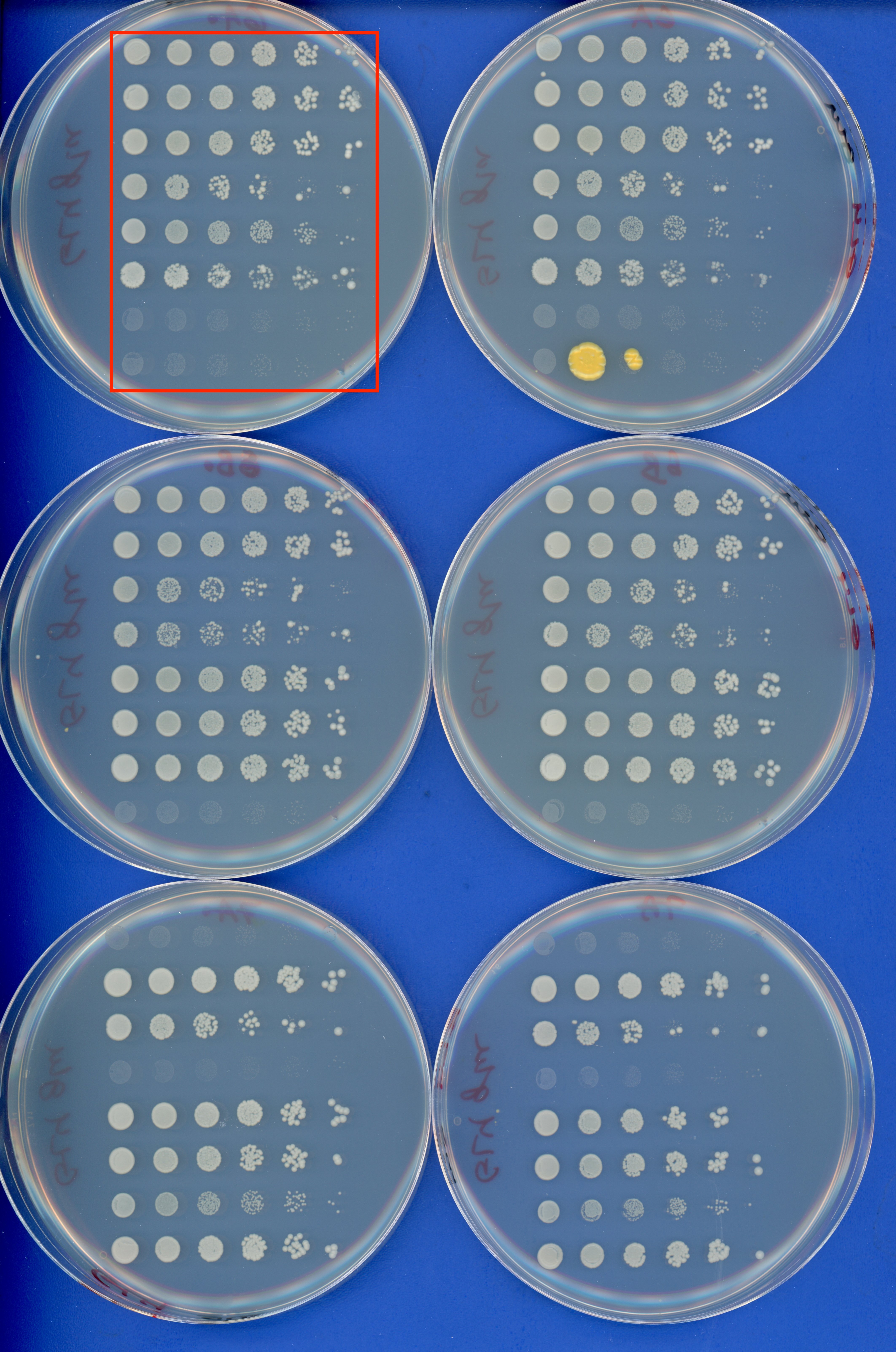

Supplement: Supplementary file 10 — Source data Fig. 6 [file 44318_2025_649_MOESM10_ESM.zip › 121174_Source_Data_Fig_6/Fig_6A_6B/Fellas_Fig_6A_GLYglu_annotated.jpg]

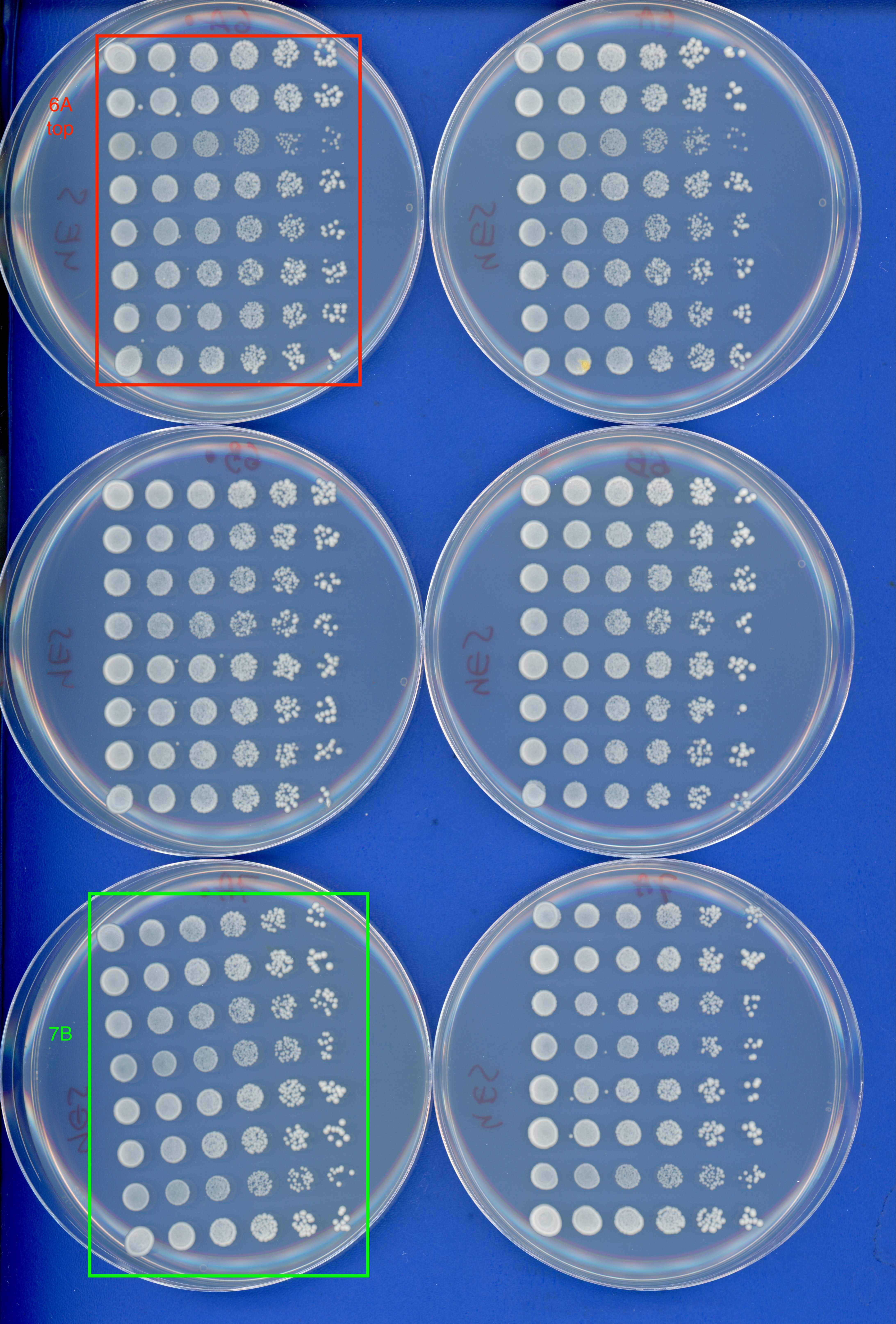

Supplement: Supplementary file 10 — Source data Fig. 6 [file 44318_2025_649_MOESM10_ESM.zip › 121174_Source_Data_Fig_6/Fig_6A_6B/Fellas_Fig_6A_7B_YES_annotated.jpg]
