# Supplementary material for: Heterochromatin epimutations impose mitochondrial dysfunction to confer antifungal resistance
Source: EMBO J. 2025 Dec 1;45(2):417–48. doi: 10.1038/s44318-025-00649-0 (PMC12811382; doi:10.1038/s44318-025-00649-0)
Supplement: Supplementary file 8 — Source data Fig. 4 [file 44318_2025_649_MOESM8_ESM.zip › 121174_Source_Data_Fig_4/Fig_4C/4C_readme.docx]

Comparisons were performed using BioVenn by contrasting the CESR or COSG induced genes with the corresponding gene lists from Fig 3B for cup1tt and ppr4D respectively.
