# Supplementary material for: Heterochromatin epimutations impose mitochondrial dysfunction to confer antifungal resistance
Source: EMBO J. 2025 Dec 1;45(2):417–48. doi: 10.1038/s44318-025-00649-0 (PMC12811382; doi:10.1038/s44318-025-00649-0)
Supplement: Supplementary file 9 — Source data Fig. 5 [file 44318_2025_649_MOESM9_ESM.zip › 121174_Source_Data_Fig_5/Fig_5B/READme.docx]

Created selection using corresponding ROIs in FIJI 220x220 pixels for each selection.

ROI files corresponding to names are also included in ROI folder. For GFP and DAPI channels max-projection tif files of images provided.

Brightness for c1(GFP_ set at 0.02-6954.40), Brightness for C2 Dapi (autoscaled).

The file names have strain number in them to identify:

B5665 GFP-pap1

B5671 GFP-pap1, hba1D

B5676 GFP-pap1, cup1tt

B5680 GFP-pap1, ppr4D
