# Supplementary figures and images for: Heterochromatin epimutations impose mitochondrial dysfunction to confer antifungal resistance (part 2 of 2)
Source: EMBO J. 2025 Dec 1;45(2):417–48. doi: 10.1038/s44318-025-00649-0 (PMC12811382; doi:10.1038/s44318-025-00649-0)

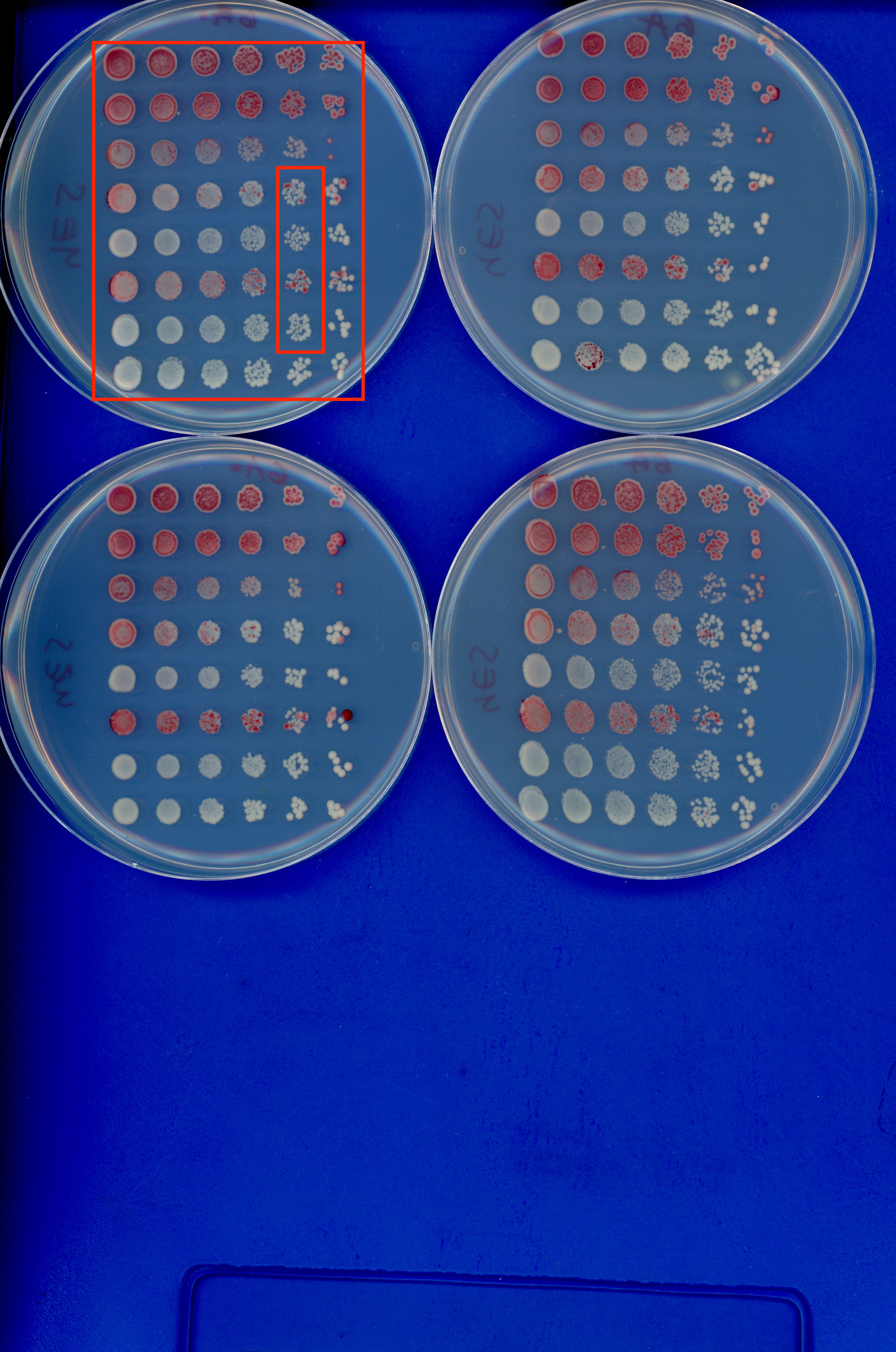

Supplement: Supplementary file 10 — Source data Fig. 6 [file 44318_2025_649_MOESM10_ESM.zip › 121174_Source_Data_Fig_6/Fig_6A_6B/Fellas_Fig_6A_TTC_annotated.jpg]

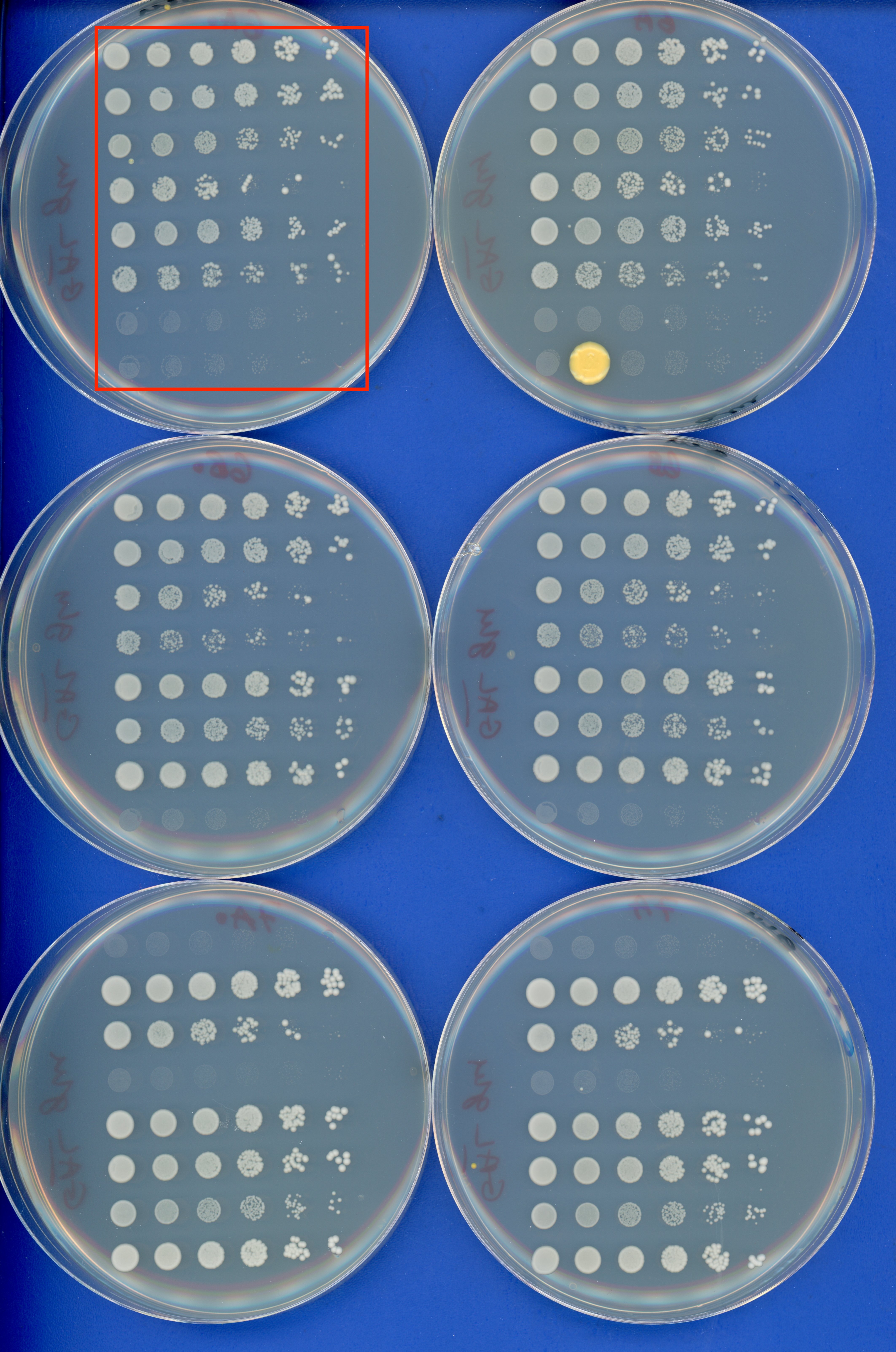

Supplement: Supplementary file 10 — Source data Fig. 6 [file 44318_2025_649_MOESM10_ESM.zip › 121174_Source_Data_Fig_6/Fig_6A_6B/Fellas_Fig_6A_GALglu_annotated.jpg]

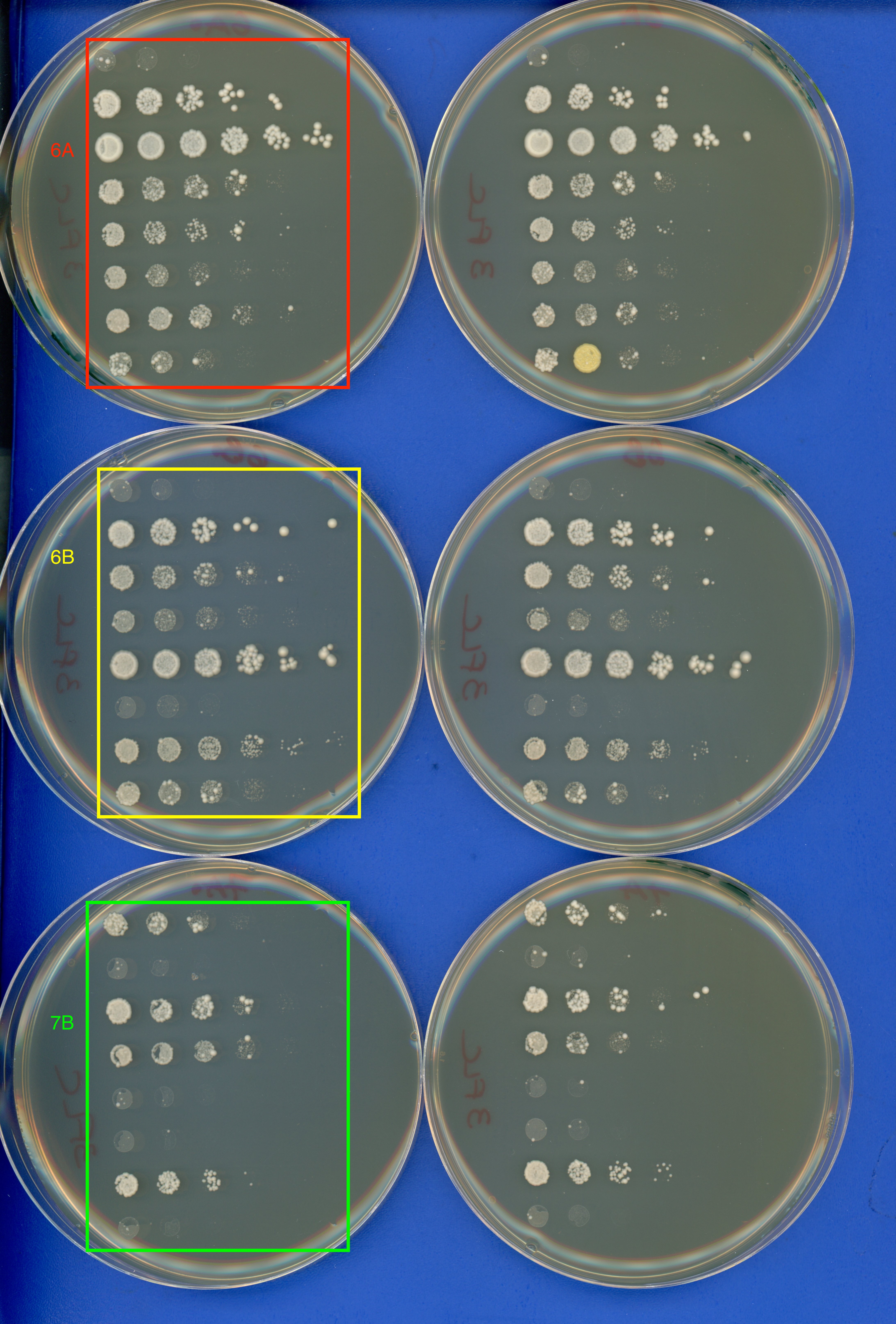

Supplement: Supplementary file 10 — Source data Fig. 6 [file 44318_2025_649_MOESM10_ESM.zip › 121174_Source_Data_Fig_6/Fig_6A_6B/Fellas_Fig_6A_6B_7B_FLC_annotated.jpg]

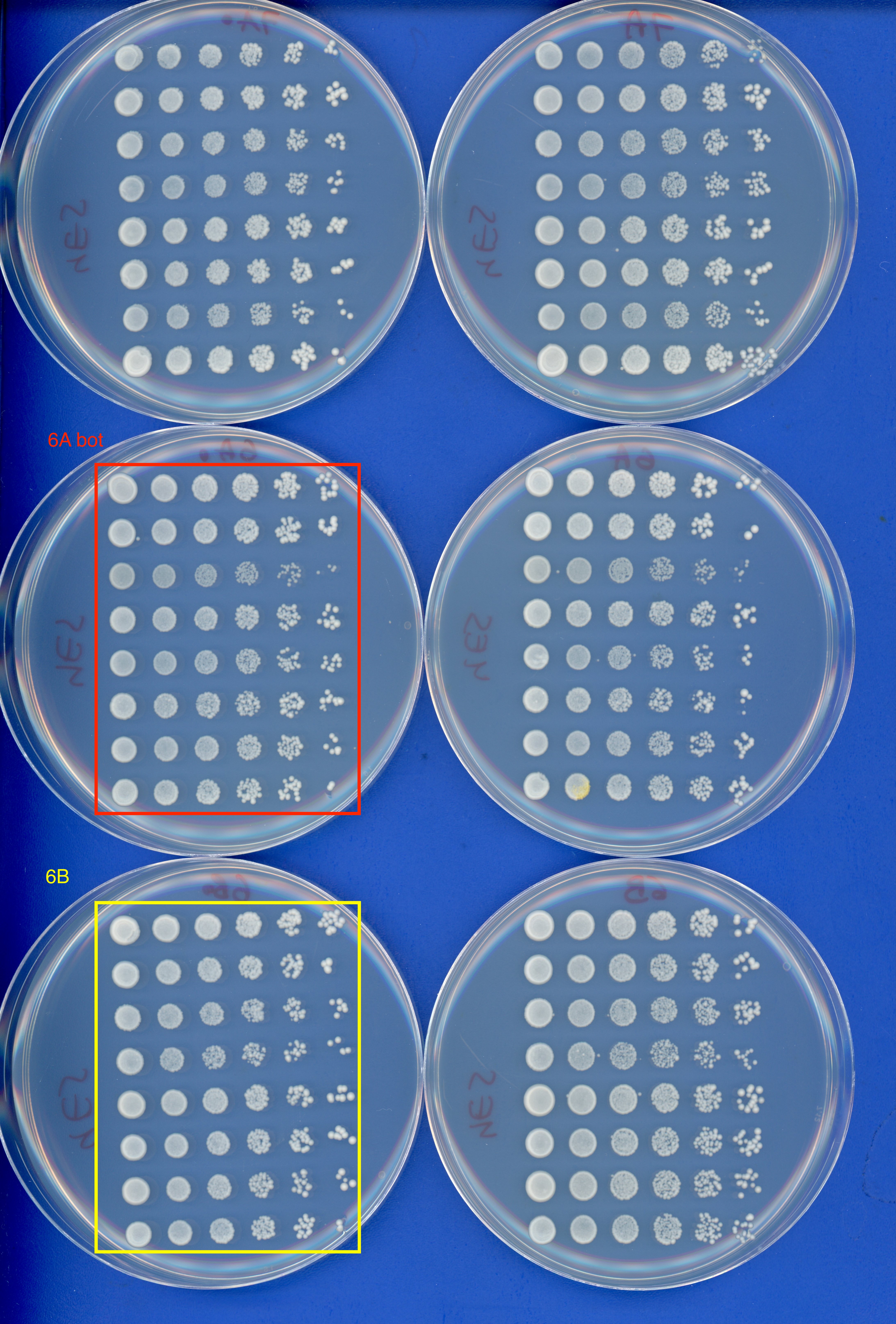

Supplement: Supplementary file 10 — Source data Fig. 6 [file 44318_2025_649_MOESM10_ESM.zip › 121174_Source_Data_Fig_6/Fig_6A_6B/Fellas_Fig_6A_6B_YES_annotated.jpg]

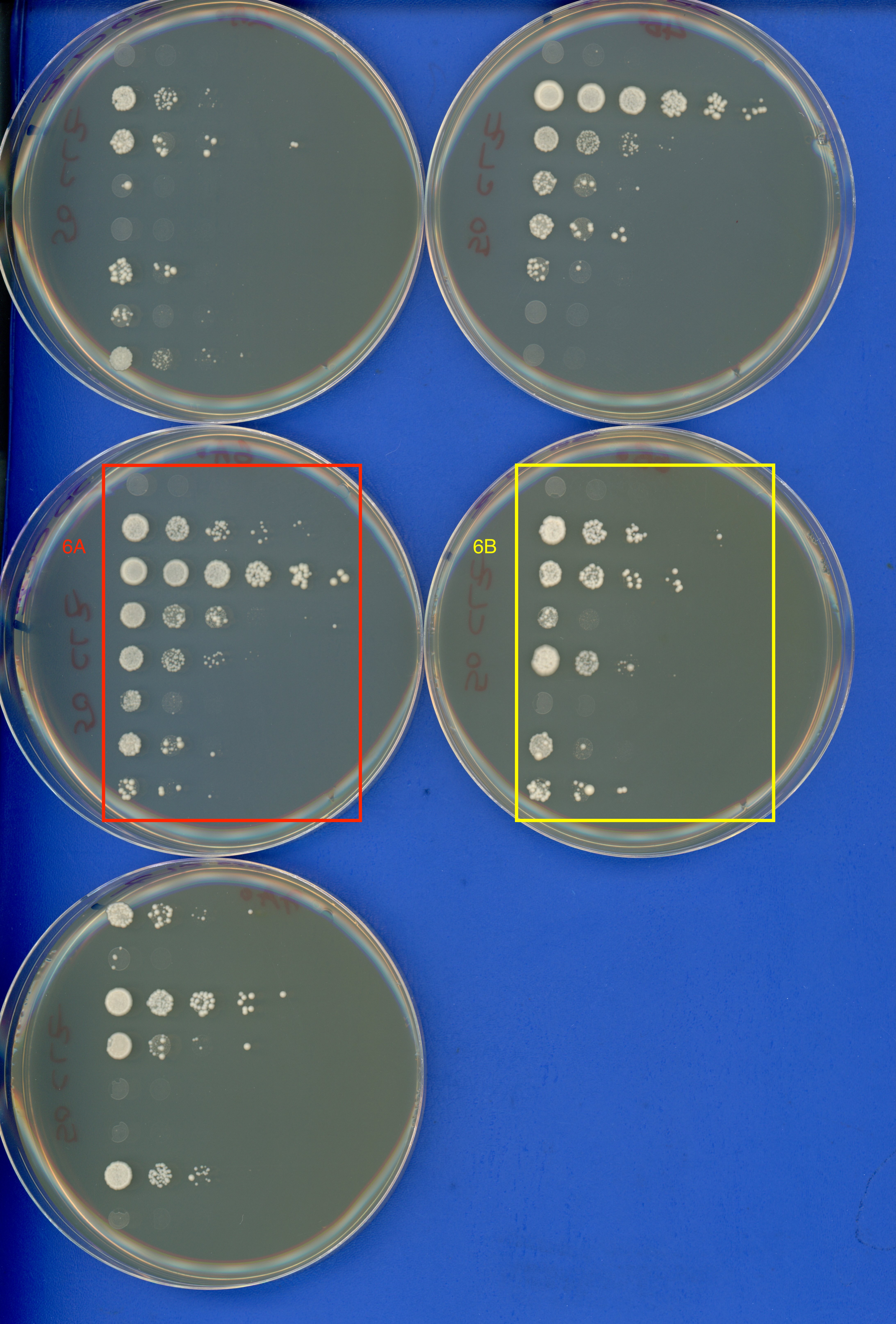

Supplement: Supplementary file 10 — Source data Fig. 6 [file 44318_2025_649_MOESM10_ESM.zip › 121174_Source_Data_Fig_6/Fig_6A_6B/Fellas_Fig_6A_6B_CLT_annotated.jpg]

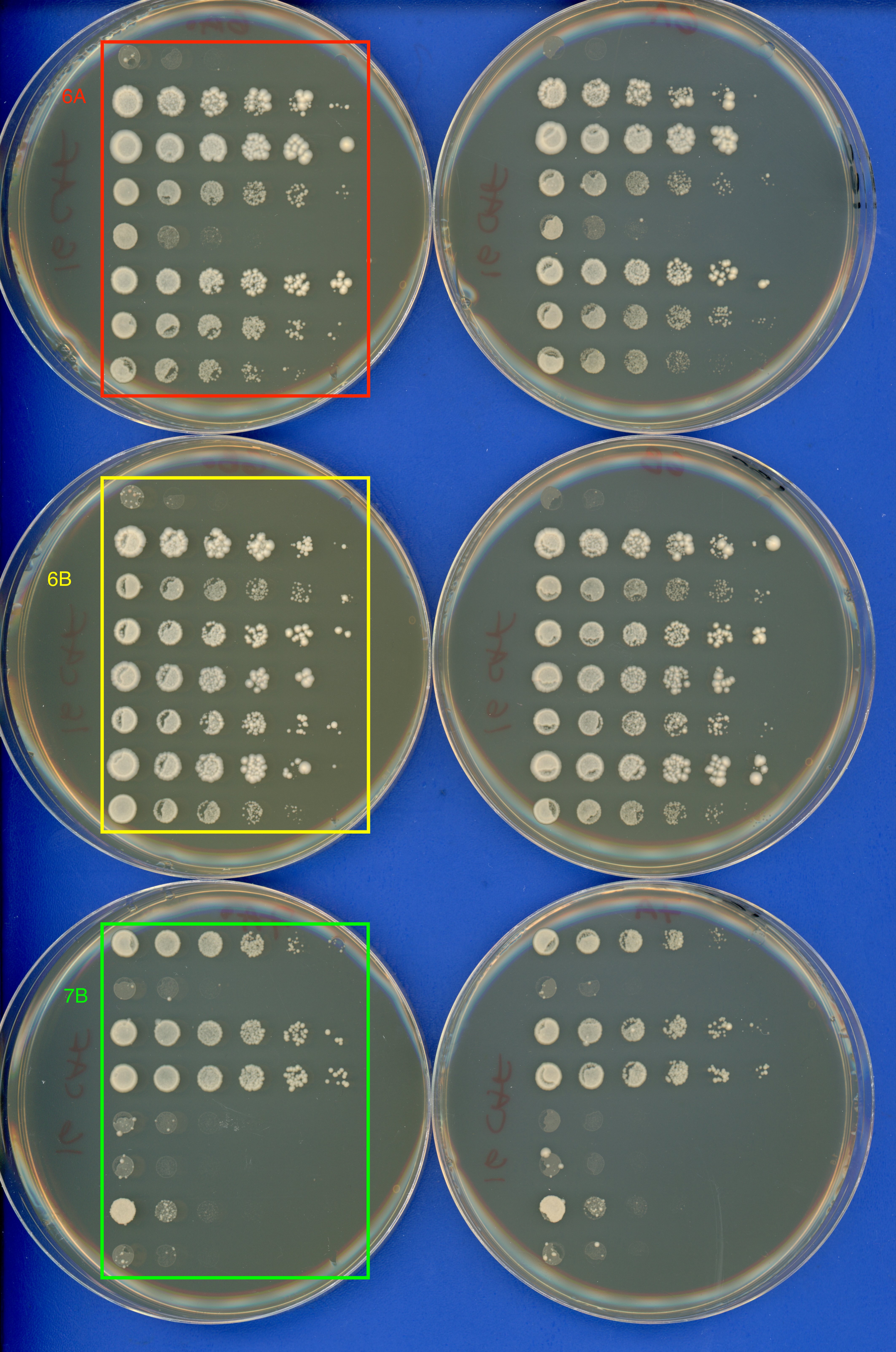

Supplement: Supplementary file 10 — Source data Fig. 6 [file 44318_2025_649_MOESM10_ESM.zip › 121174_Source_Data_Fig_6/Fig_6A_6B/Fellas_Fig_6A_6B_7B_CAF_annotated.jpg]

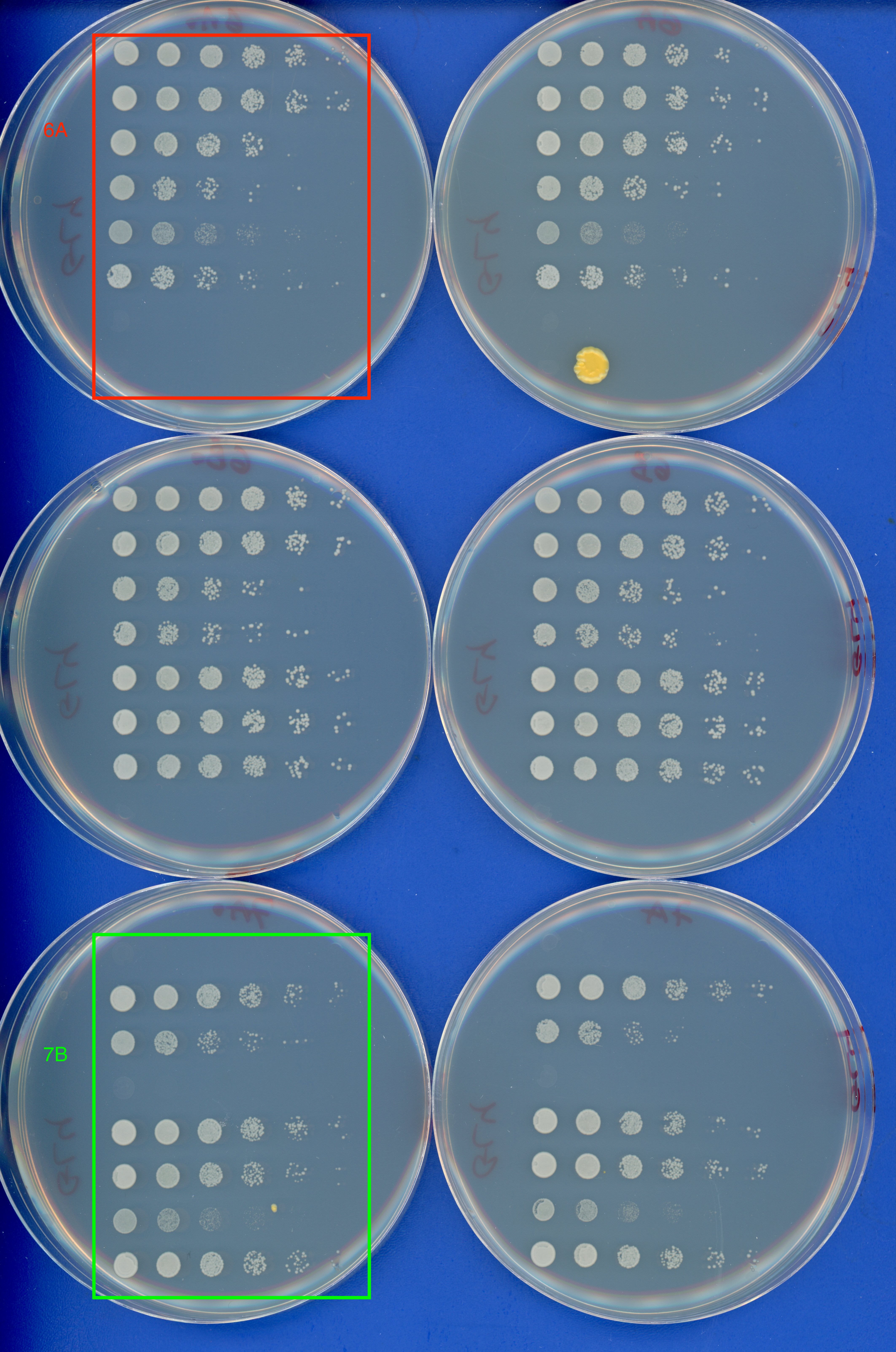

Supplement: Supplementary file 10 — Source data Fig. 6 [file 44318_2025_649_MOESM10_ESM.zip › 121174_Source_Data_Fig_6/Fig_6A_6B/Fellas_Fig_6A_7B_GLY_annotated.jpg]

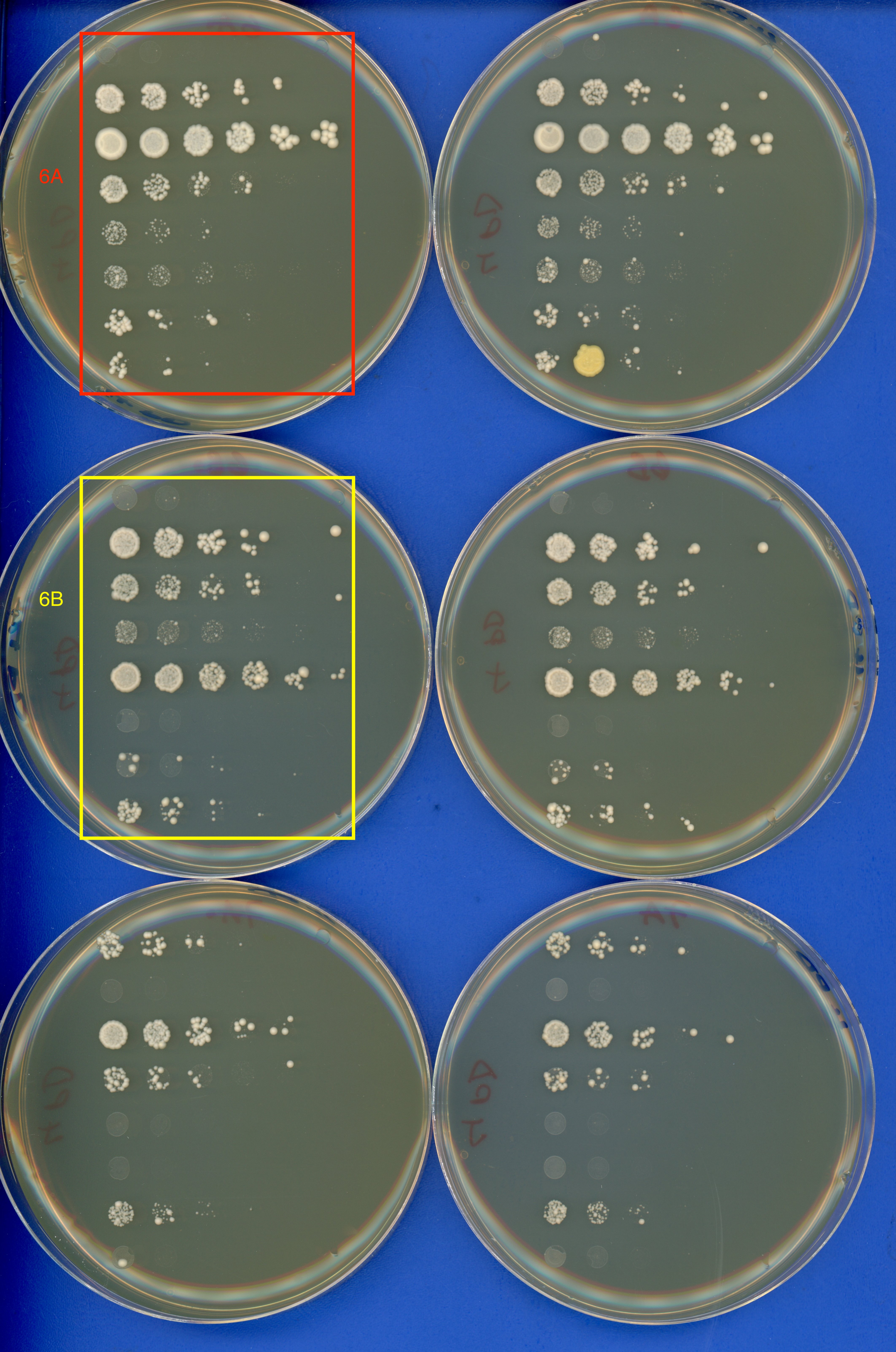

Supplement: Supplementary file 10 — Source data Fig. 6 [file 44318_2025_649_MOESM10_ESM.zip › 121174_Source_Data_Fig_6/Fig_6A_6B/Fellas_Fig_6A_6B_PRD_annotated.jpg]

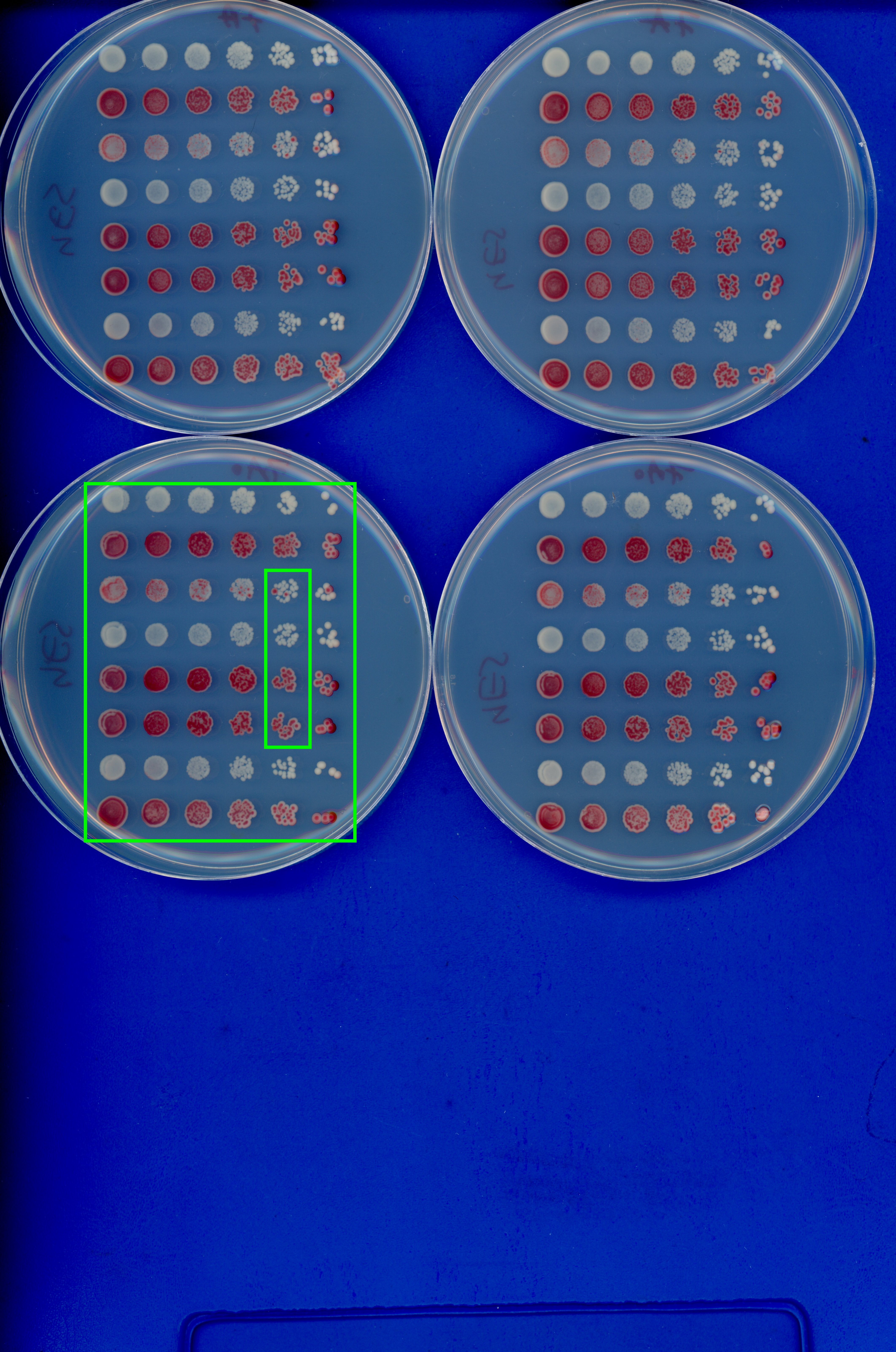

Supplement: Supplementary file 11 — Source data Fig. 7 [file 44318_2025_649_MOESM11_ESM.zip › 121174_Source_Data_Fig_7/Fig_7B/Fellas_Fig_7B_annotated.jpg]
