# Supplementary material for: Heterochromatin epimutations impose mitochondrial dysfunction to confer antifungal resistance
Source: EMBO J. 2025 Dec 1;45(2):417–48. doi: 10.1038/s44318-025-00649-0 (PMC12811382; doi:10.1038/s44318-025-00649-0)
Supplement: Supplementary file 12 — Expanded View Figures [file 44318_2025_649_MOESM12_ESM.pdf]

Expanded View Figures

Figure EV1. Deletion of genes within loci where heterochromatin islands are located in UR4, UR5 and UR6 epimutants.

(**A**) Genes encompassed in heterochromatin islands present in UR4, UR5 and UR6 epimutants. Schematics of loci containing ectopic heterochromatin islands. Protein-coding genes only are indicated. Shaded blocks indicate the regions deleted in multiple-gene deletions, with expanded view below, where some genes are indicated with abbreviated names. Left: UR4 heterochromatin island, ChrII (0–60 kb); centre: UR5 heterochromatin island, ChrI (4230–4260 kb); right: UR6 heterochromatin island, ChrII (3615–3640 kb) ChIP-seq data displayed is from (Torres-Garcia et al, 2020b). (**B**) Growth assay to assess growth of deletion strains on caffeine. Single genes or regions containing multiple genes were deleted from wild-type cells and the ability of resultant strains to grow on media containing caffeine assessed. Five-fold serial dilutions of indicated strains spotted onto non-selective plates (NS; YES media) or plates containing the indicated concentrations of caffeine. Plates photographed after 2–8 days at 32 °C. (**C**) Growth assay to assess resistance to caffeine and antifungal drugs. Strains which showed some resistance to caffeine in (**B**) were retested in growth assays to assess ability to grow on various insults. Five-fold serial dilutions of indicated strains spotted onto non-selective plates (NS; YES media) or plates containing 14 mM or 16 mM caffeine (CAF), 0.3 mM fluconazole (FLC), 50 ng/ml clotrimazole (CLT). Plates photographed after 2–8 days at 32 °C.

© The Author(s)

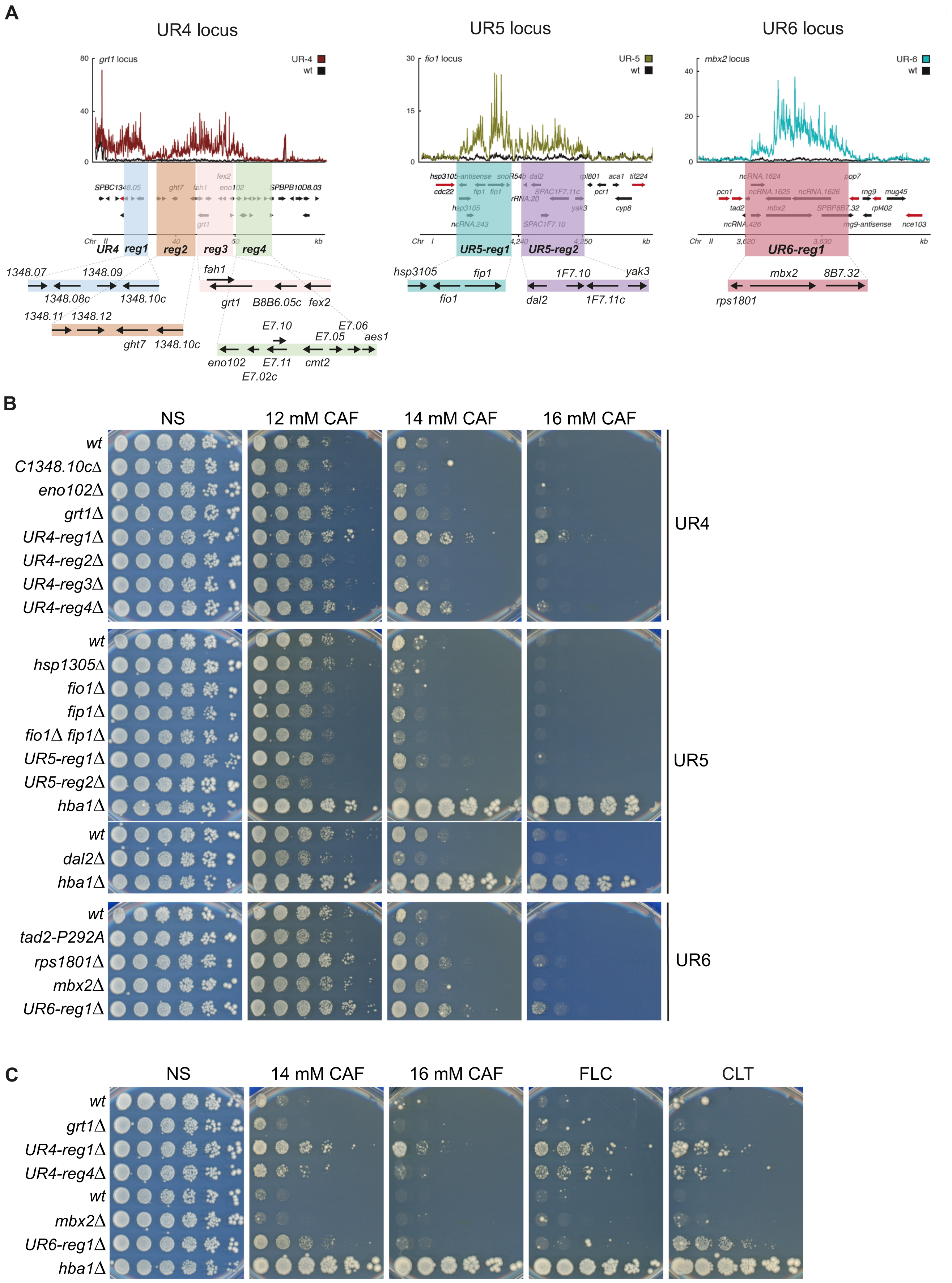
A
UR4 locus
UR5 locus
UR6 locus
reg1
reg2
reg3
reg4
UR5-reg1
UR5-reg2
UR6-reg1
B
NS
12 mM CAF
14 mM CAF
16 mM CAF
wt
C1348.10cΔ
eno102Δ
grt1Δ
UR4-reg1Δ
UR4-reg2Δ
UR4-reg3Δ
UR4-reg4Δ
UR4
hsp1305Δ
fio1Δ
fip1Δ
fio1Δ fip1Δ
UR5-reg1Δ
UR5-reg2Δ
hba1Δ
dal2Δ
UR5
tad2-P292A
rps1801Δ
mbx2Δ
UR6-reg1Δ
UR6
C
FLC
CLT

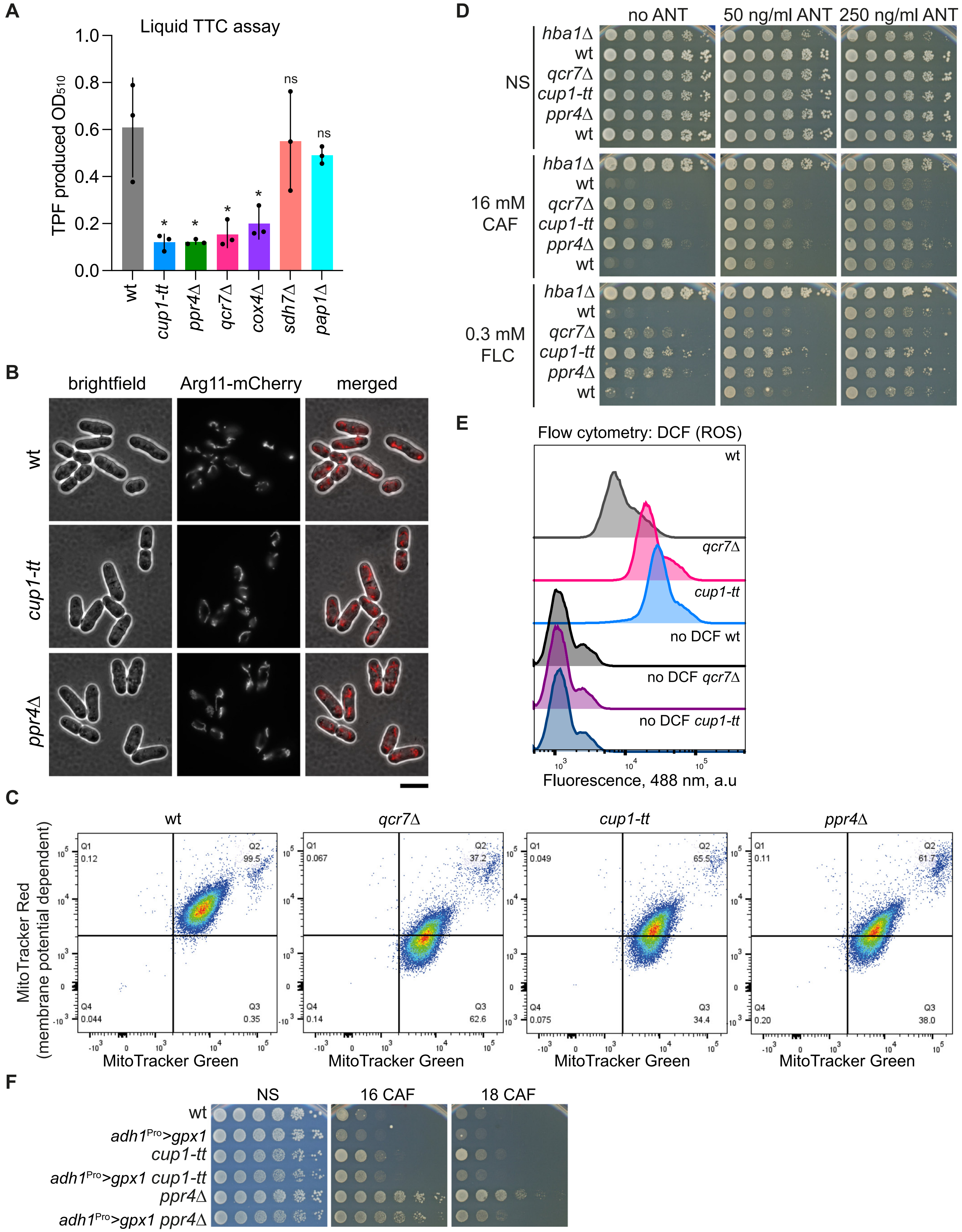
A
Liquid TTC assay
TPF produced OD510
1.0
0.8
0.6
0.4
0.2
0.0
ns
ns
*
*
*
*
wt
cup1-tt
ppr4Δ
qcr7Δ
cox4Δ
sdh7Δ
pap1Δ
B
brightfield
Arg11-mCherry
merged
wt
cup1-tt
ppr4Δ
C
wt
qcr7Δ
cup1-tt
ppr4Δ
MitoTracker Red (membrane potential dependent)
MitoTracker Green
Q1 0.12
Q2 99.5
Q4 0.044
Q3 0.35
Q1 0.067
Q2 37.2
Q4 0.14
Q3 62.6
Q1 0.049
Q2 65.5
Q4 0.075
Q3 34.4
Q1 0.11
Q2 61.7
Q4 0.20
Q3 38.0
D
no ANT
50 ng/ml ANT
250 ng/ml ANT
NS
16 mM CAF
0.3 mM FLC
hba1Δ
wt
qcr7Δ
cup1-tt
ppr4Δ
wt
E
Flow cytometry: DCF (ROS)
wt
qcr7Δ
cup1-tt
no DCF wt
no DCF qcr7Δ
no DCF cup1-tt
Fluorescence, 488 nm, a.u
F
NS
16 CAF
18 CAF
wt
adh1Pro>gpx1
cup1-tt
adh1Pro>gpx1 cup1-tt
ppr4Δ
adh1Pro>gpx1 ppr4Δ

Figure EV2. Deficiency of Cup1, Ppr4 or ETC components cause respiratory deficiency.

(**A**) Liquid Tetrazolium assay for respiratory competence. Cells were incubated in 2,3,5-Triphenyltetrazolium Chloride (TTC) for 30 min before cell lysis and extraction with DMSO to release 1,3,5-triphenylformazan (TPF). TPF was measured by absorbance at 510 nm. Data are mean and standard deviation from three biological replicates. *p* values determined by two-tailed Student's t-test: $*p < 0.05$; $**p < 0.01$; ns, not significant. (**B**) Assessment of mitochondrial morphology. Fluorescence microscopy images of live cells of indicated strains expressing the mitochondrial protein Arg11 (Rutherford et al, 2024) tagged with mCherry (Torres-Garcia et al, 2020b). Fluorescence images are scaled relative to the brightest image. Scale bar, 10 µm. (**C**) Flow cytometry of cells of the indicated strains stained with Mitotracker RedCMXRos and Mitotracker Green which are respectively dependent and independent of mitochondrial membrane potential (Uehara et al, 2021). A representative experiment is shown. Percentage of cells in each quadrant are indicated: Q1, high red, low green; Q2, high red, high green; Q3, low red, high green; Q4, low red, low green. (**D**) Growth assay to assess impact of Antimycin A on resistance to insults. Five-fold serial dilutions of indicated strains spotted onto non-selective plates (NS; YES media containing 0.135% DMSO) or plates containing the indicated concentrations of caffeine or fluconazole, along with indicated concentrations of Antimycin A. Plates photographed after 2–8 days at 32 °C. (**E**) Flow cytometry of cells stained with DCHF-DA or unstained wt, *qcr7*Δ and *cup1-tt* cells are shown. (**F**) Growth assay to assess impact of overexpression of hydrogen peroxide scavenger Gpx1 on resistance to caffeine. Where indicated, cells contained an additional copy of *gpx1* under control of the strong *adh1* promoter. Five-fold serial dilutions of indicated strains spotted onto non-selective plates (NS; YES media) or plates containing the indicated concentrations of caffeine. Plates photographed after 2–8 days at 32 °C.

© The Author(s)

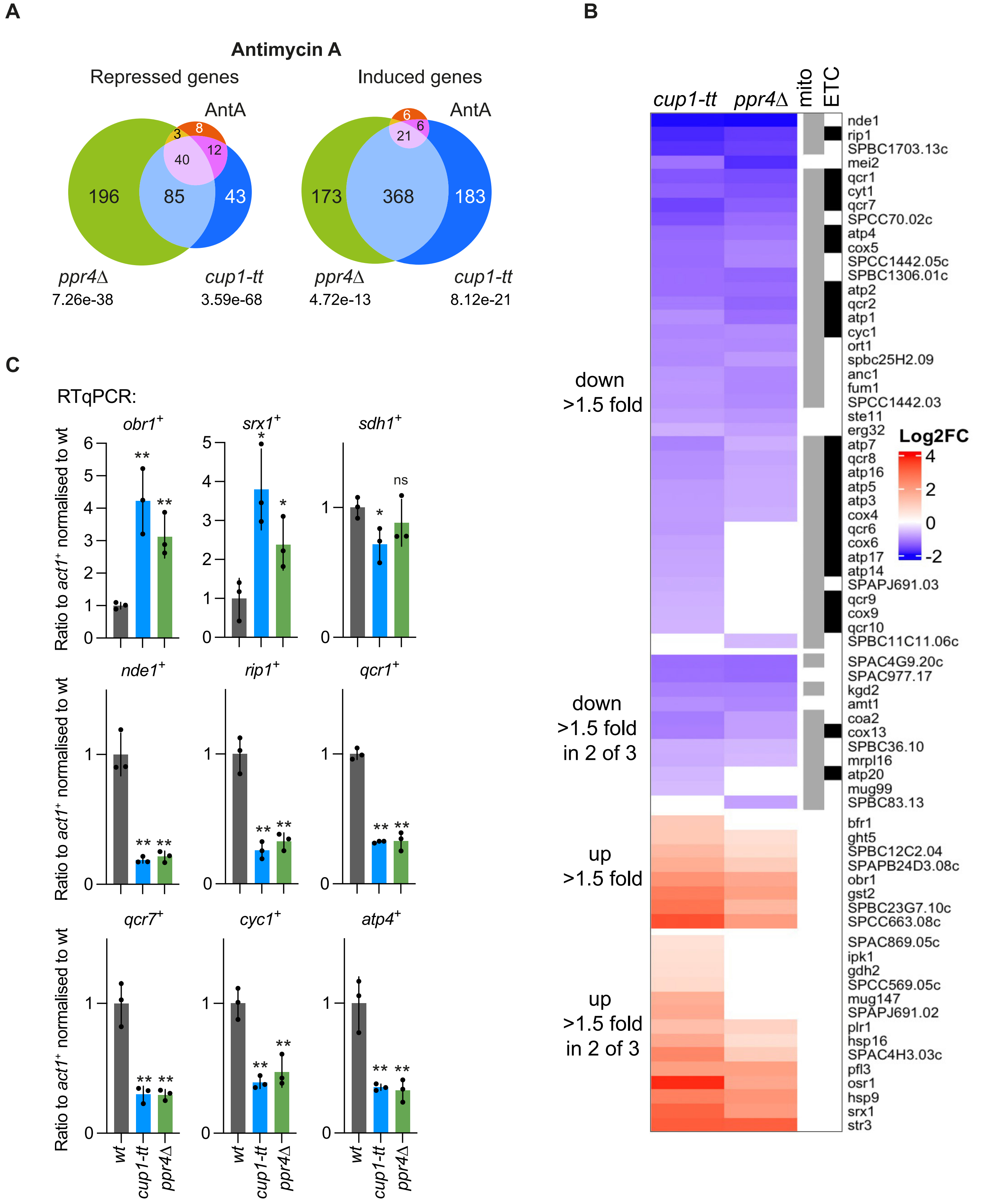
A
Antimycin A
Repressed genes
Induced genes
AntA
ppr4Δ 7.26e-38
cup1-tt 3.59e-68
ppr4Δ 4.72e-13
cup1-tt 8.12e-21
196 85 43 40 3 8 12
173 368 183 21 6 6
B
cup1-tt ppr4Δ mito ETC
down >1.5 fold
down >1.5 fold in 2 of 3
up >1.5 fold
up >1.5 fold in 2 of 3
Log2FC
nde1 rip1 SPBC1703.13c mei2 qcr1 cyt1 qcr7 SPCC70.02c atp4 cox5 SPCC1442.05c SPBC1306.01c atp2 qcr2 atp1 cyc1 ort1 spbc25H2.09 anc1 fum1 SPCC1442.03 ste11 erg32 atp7 qcr8 atp16 atp5 atp3 cox4 qcr6 cox6 atp17 atp14 SPAPJ691.03 qcr9 cox9 qcr10 SPBC11C11.06c
SPAC4G9.20c SPAC977.17 kgd2 amt1 coa2 cox13 SPBC36.10 mrpl16 atp20 mug99 SPBC83.13
bfr1 ght5 SPBC12C2.04 SPAPB24D3.08c obr1 gst2 SPBC23G7.10c SPCC663.08c
SPAC869.05c ipk1 gdh2 SPCC569.05c mug147 SPAPJ691.02 plr1 hsp16 SPAC4H3.03c pfl3 osr1 hsp9 srx1 str3
C
RTqPCR:
obr1+ srx1+ sdh1+
nde1+ rip1+ qcr1+
qcr7+ cyc1+ atp4+
Ratio to act1+ normalised to wt
wt cup1-tt ppr4Δ
ns

Figure EV3. Transcriptional profiles of cells defective for Cup1 or Ppr4 overlap with mitonuclear retrograde response.

(**A**) Comparison of *cup1-tt* and *ppr4Δ* with antimycin A treatment (Malecki et al, 2016). Left: Venn diagram of comparison between genes repressed in *cup1-tt* (dark blue) and *ppr4Δ* (green) and downregulated in response to Antimycin A, which activates the mitonuclear retrograde pathway (pink). Right: Venn diagram of comparison between genes upregulated in *cup1-tt* (dark blue) and *ppr4Δ* (green) and induced in response to Antimycin A (pink). *p*-values represent probabilities that the observed overlaps occurred by chance, as determined by hypergeometric test. (**B**) Comparison of *cup1-tt* and *ppr4Δ* with MNR conditions. Heatmap of differentially expressed genes in *cup1-tt* vs wt and *ppr4Δ* vs wt that are altered in MNR (Malecki et al, 2016). Genes (rows) are separated into 4 sections: change (up or down) 1.5-fold in all 3 conditions (AntA treatment, *rpm1Δ* and *rep1Δ*) and changes (up or down) 1.5-fold in at least 2 out of 3 of these conditions (AntA treatment, *rpm1Δ* and *rep1Δ*), as previously described in MNR (Malecki et al, 2016). Each column represents the genotype (*cup1-tt* or *ppr4Δ* compared to wt). Only differentially expressed genes with fold-change value ≥ 1.5 and FDR-adjusted *p*-value < 0.05 from transcriptomic analysis of *cup1-tt* or *ppr4Δ* compared to wt are used in the construction of this heatmap. Each row corresponds to a gene, labelled on right. Colour gradient key represents $\log_2$FC, red for upregulated and blue for downregulated transcripts (Malecki et al, 2016). Genes encoding mitochondrial proteins are indicated on the right in grey and ETC/ATP synthase subunits in black. (**C**) Transcript levels of ETC and antioxidant genes. Quantification by RT-qPCR of transcript levels of antioxidant genes (*obr1*$^+$, *srx1*$^+$) and nuclear-encoded ETC genes in the indicated strains. It has been shown that ETC Complex II genes such as *sdh1*$^+$ are not strongly repressed by mitochondrial dysfunction (Malecki et al, 2016). Data are mean and standard deviation from three biological replicates. *p* values determined by two-tailed Student's t-test: *$p < 0.05$; **$p < 0.01$; ns, not significant.

 © The Author(s)

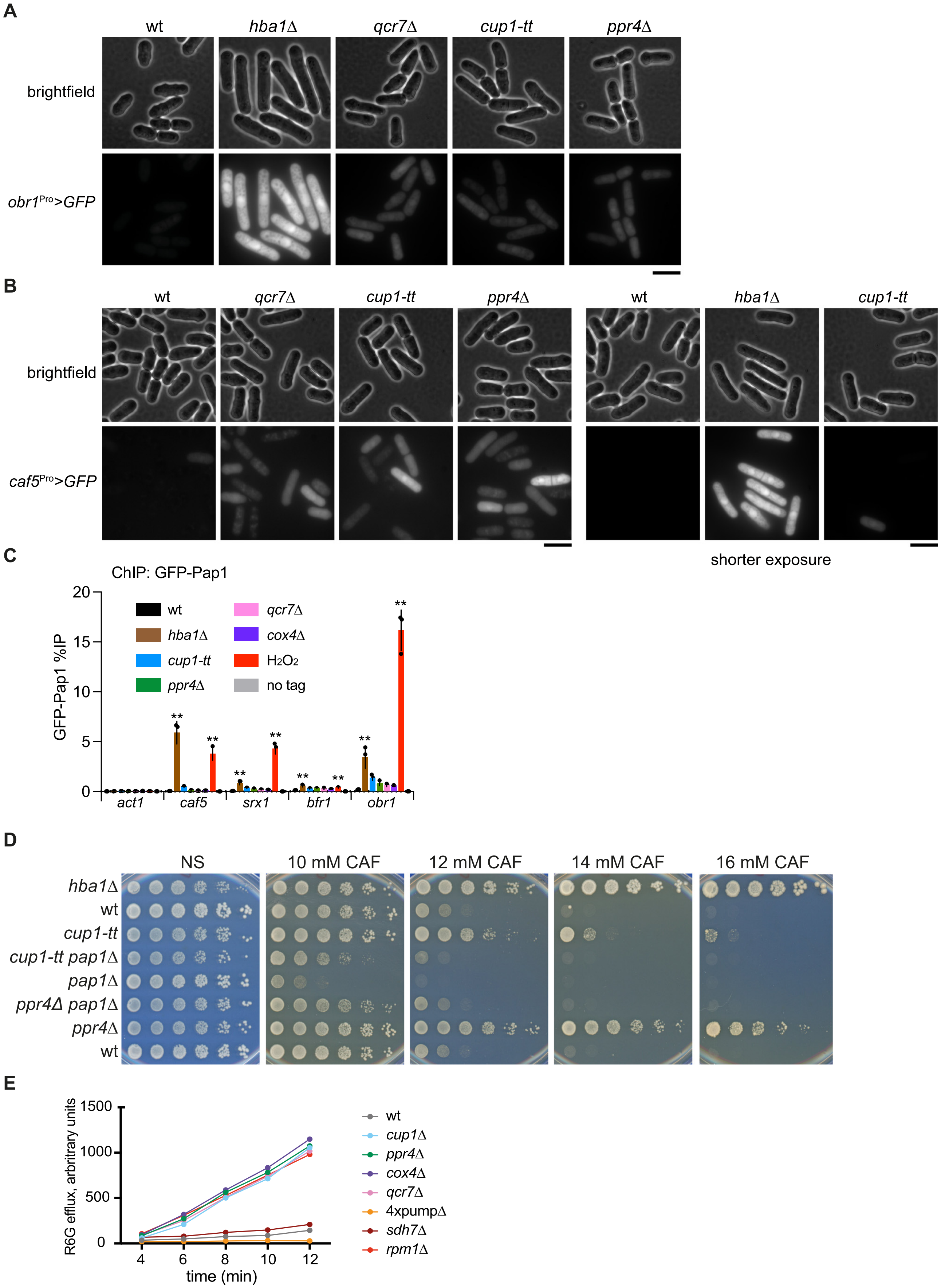
A
wt
hba1Δ
qcr7Δ
cup1-tt
ppr4Δ
brightfield
obr1Pro>GFP
B
wt
qcr7Δ
cup1-tt
ppr4Δ
wt
hba1Δ
cup1-tt
brightfield
caf5Pro>GFP
shorter exposure
C
ChIP: GFP-Pap1
GFP-Pap1 %IP
wt
hba1Δ
cup1-tt
ppr4Δ
qcr7Δ
cox4Δ
H2O2
no tag
act1
caf5
srx1
bfr1
obr1
D
NS
10 mM CAF
12 mM CAF
14 mM CAF
16 mM CAF
hba1Δ
wt
cup1-tt
cup1-tt pap1Δ
pap1Δ
ppr4Δ pap1Δ
ppr4Δ
wt
E
R6G efflux, arbitrary units
time (min)
wt
cup1Δ
ppr4Δ
cox4Δ
qcr7Δ
4xpumpΔ
sdh7Δ
rpm1Δ

Figure EV4. Cup1, Ppr4 and ETC deficient cells activate the Pap1-dependent stress response and show increased efflux.

(**A**) Pap1-dependent *obr1*-promoter reporter assay. Fluorescence microscopy images of live cells of indicated strains containing GFP under control of the Pap1-dependent *obr1* promoter. Fluorescence images are scaled relative to the brightest image (*hba1*Δ). Scale bar, 10 μm. (**B**) Pap1-dependent *caf5* promoter reporter assay. Fluorescence and brightfield images live cells of indicated strains containing GFP under control of the Pap1-dependent *caf1* promoter. Left panels: 2000 ms exposure. Right panels: 300 ms exposure. Fluorescence images are scaled relative to the brightest image in each set of images. Scale bar, 10 μm. (**C**) GFP-Pap1 ChIP-qPCR of the indicated strains and GFP-Pap1 wild-type cells treated with 0.2 mM hydrogen peroxide for 30 min. Promoter regions of the *caf5* and *bfr1* (transmembrane transporters), *srx1* (sulfiredoxin), and *obr1* (dehydrogenase) genes were analysed by qPCR to determine GFP-Pap1% immunoprecipitation. *act1* serves as negative control locus. Data are mean and standard deviation from three biological replicates. p values determined by two-tailed Student's t-test: *$p < 0.05$; **$p < 0.01$; ns, not significant. Only p values for wt vs *hba1*Δ and H_2O_2 treatment are indicated. p values for other mutants are shown in Fig. 5C. (**D**) Growth assay to assess impact of loss of Pap1 on *cup1-tt* and *ppr4*Δ mutants. Five-fold serial dilutions of indicated strains spotted onto non-selective plates (NS; YES media) or plates containing the indicated concentrations of caffeine. Plates photographed after 2–8 days at 32 °C. (**E**) Efflux of Rhodamine 6G (R6G) from cells. Cells of the indicated strains were preloaded with R6G and enabled to perform efflux when supplied with glucose (in YES). R6G released to the media over the indicated time-period was measured. Representative example shown. *4xpump*Δ strain four transmembrane transporters are absent (Bfr1, Pmp1, Caf5, Mfs1) along with Pap1 and Prt1.

 © The Author(s)

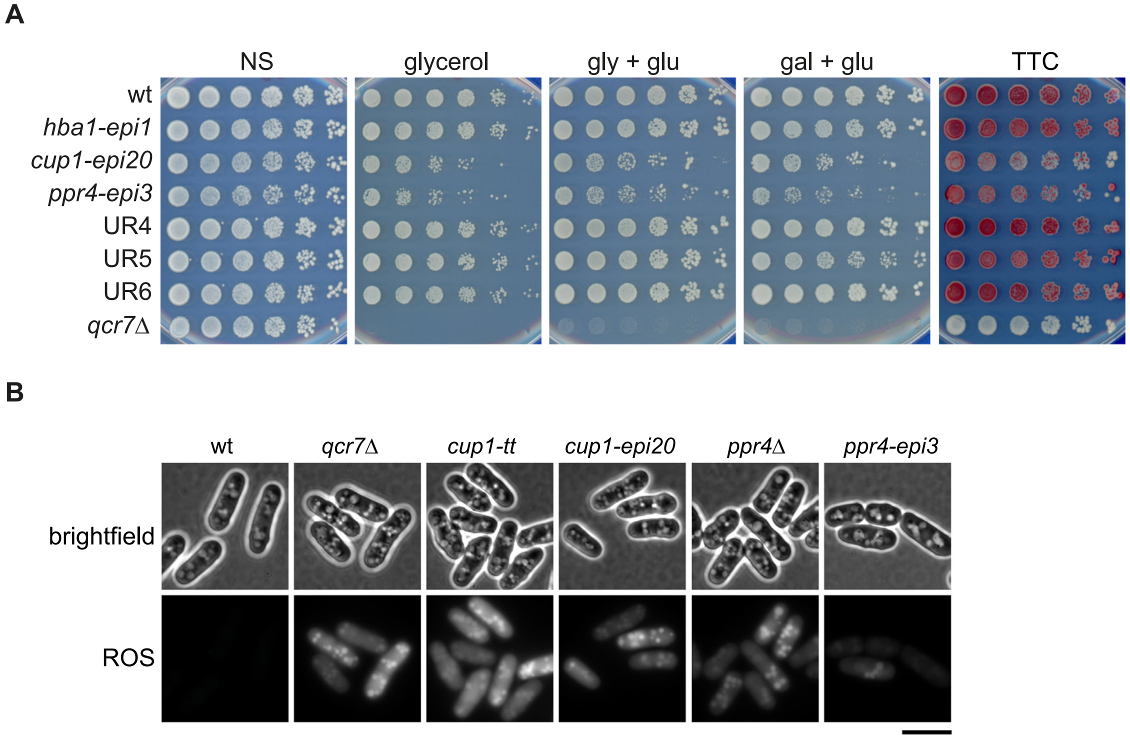

Figure EV5. Assessment of mitochondrial competence and ROS levels in epimutants.

(**A**) Growth assay to assess respiratory competence. Five-fold serial dilutions of indicated strains spotted onto non-selective plates (NS; YES media), or YES plates in which glucose was replaced with 3% glycerol or 3% glycerol + 0.1% glucose (gly + glu) or 2% galactose + 0.1% glucose (gal + glu). Plates photographed after 2–8 days at 32 °C. TTC: after 3 days' growth colonies on YES plate were overlaid with TTC-containing agarose and incubated for ~24 h to assess respiratory competence. (**B**) DCHF-DA staining to assess levels of reactive oxygen species. Cells of the indicated strains were incubated in the ROS indicator DCFH-DA, which is converted to fluorescent DCF in the presence of ROS, and imaged under brightfield and 488 nm illumination. Scale bar, 10 μm.

© The Author(s)

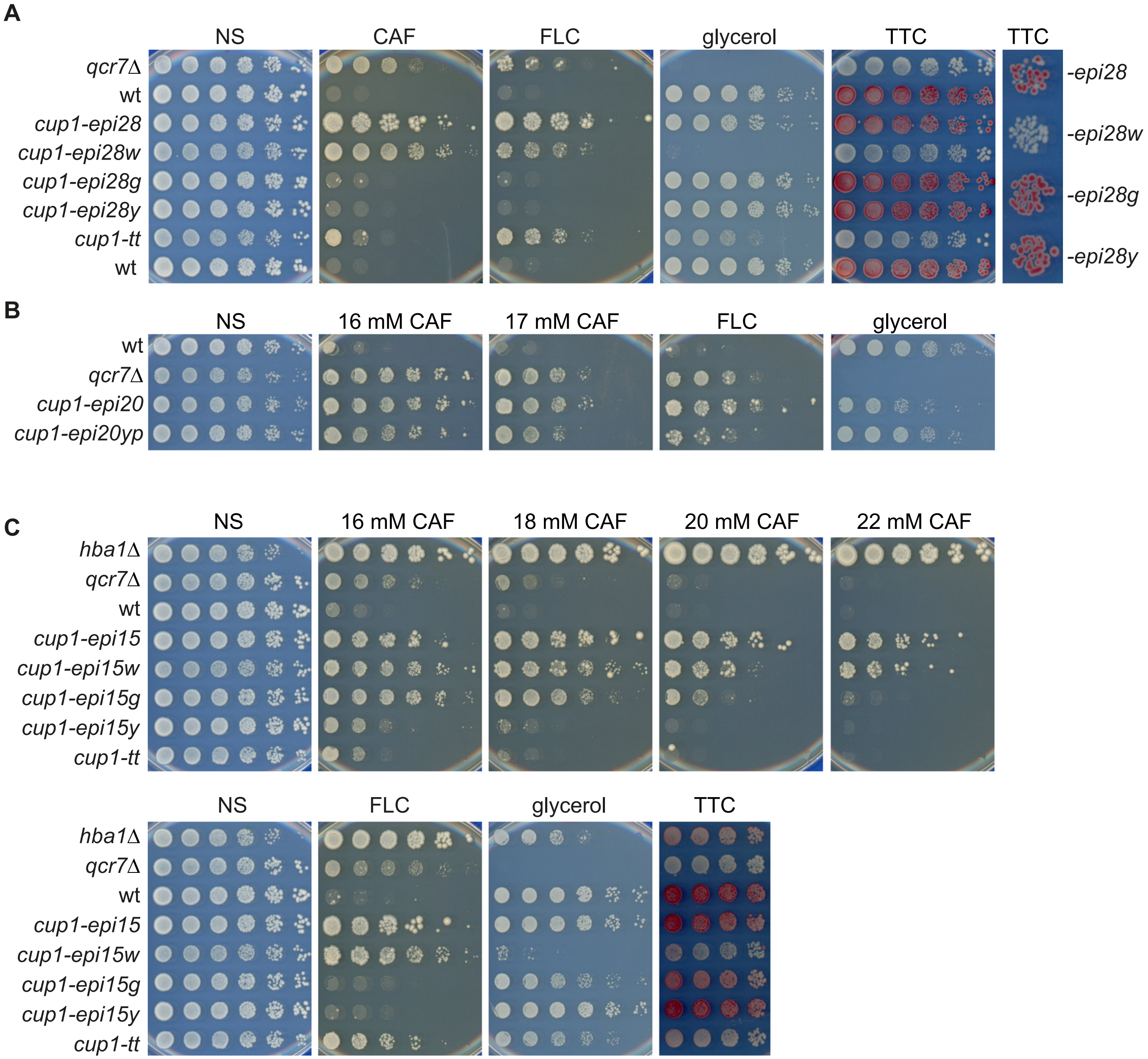

Figure EV6. Phenotypes of *cup1* epimutant subpopulations.

(**A–C**) Growth assays to assess resistance to insults and respiratory competence of isolates derived from *cup1* epimutant mixed populations: Panels show five-fold serial dilutions of indicated isolates spotted onto non-selective plates (NS; YES media) or plates containing indicated concentrations of caffeine, 0.3 mM fluconazole (FLC), YES containing 3% glycerol rather than glucose, and TTC overlay to assay respiratory competence. (**A**) *cup1-epi28* and derivatives; (**B**) *cup1-epi20* and *cup1-epi20yp* derivative; (**C**) *cup1-epi15* and derivatives. The NS panel is duplicated to allow comparison within each row.

 © The Author(s)
